# New synthesis of a late-stage tetracyclic key intermediate of lumateperone

**DOI:** 10.3762/bjoc.18.66

**Published:** 2022-06-10

**Authors:** Mátyás Milen, Bálint Nyulasi, Tamás Nagy, Gyula Simig, Balázs Volk

**Affiliations:** 1 Egis Pharmaceuticals Plc., Directorate of Drug Substance Development, 1475 Budapest, P.O. Box 100, Hungary

**Keywords:** drug substance, indole synthesis, key intermediate, protecting group, telescoping

## Abstract

New approaches have been tested for the synthesis of lumateperone intermediates. As a result of these efforts, a novel synthesis of the late-stage tetracyclic key intermediate of lumateperone starting from the commercially available quinoxaline is described. The tetracyclic skeleton was constructed by the reaction of 1-trifluoroacetyl-4-aminoquinoxaline with ethyl 4-oxopiperidine-1-carboxylate in a Fischer indole synthesis. The inexpensive starting material*,* the efficient synthetic steps, and the avoidance of the borane-based reduction step provide a reasonable potential for scalability.

## Introduction

**Figure 1:**
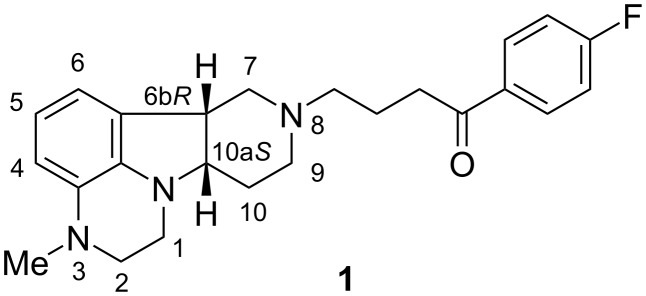
Structure of lumateperone.

Lumateperone (**1**) is a recently launched drug marketed for the treatment of schizophrenia and developed for further neuropsychiatric and neurological disorders [1–6] (Figure 1).

**Scheme 1:**
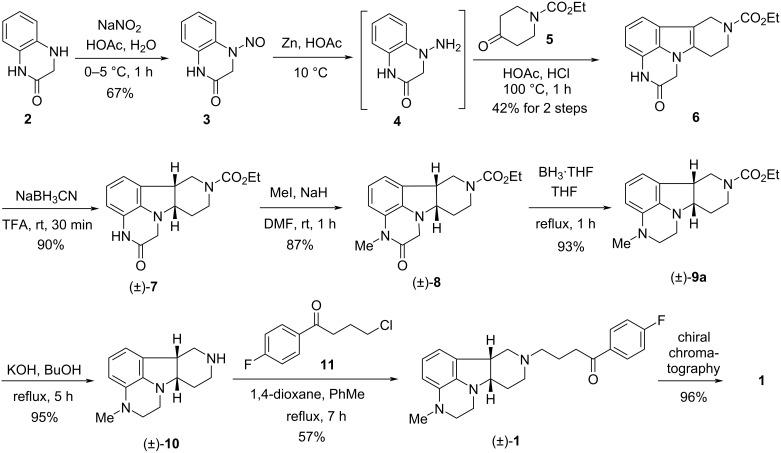
First synthetic route leading to lumateperone (**1**).

The first synthesis of lumateperone (**1**, Caplyta^®^), developed by Intra-Cellular Therapies, was disclosed in the basic patent and also discussed in a later scientific publication [7–8] (Scheme 1). According to this procedure, treatment of the commercially available, but quite expensive 3,4-dihydroquinoxalin-2(1*H*)-one (**2**) with sodium nitrite gave the *N*-nitroso derivative **3**. Reduction of the latter with zinc to the hydrazine derivative **4**, followed by a Fischer indole synthesis with ethyl 4-oxopiperidine-1-carboxylate (**5**) provided tetracyclic compound **6**. Its reduction with sodium cyanoborohydride in trifluoroacetic acid (TFA) to *cis-*indoline derivative (±)-**7**, followed by *N*-methylation [(±)-**8**] and reduction of the oxo group with borane·THF complex in THF led to compound (±)*-***9a**. Subsequent desethoxycarbonylation afforded compound (±)-**10**, which was *N*-alkylated with 4-chloro-1-(4-fluorophenyl)butan-1-one (**11**) to give the racemic form of lumateperone [(±)-**1**]. Lumateperone (**1**) was finally obtained by chiral chromatography.

**Scheme 2:**
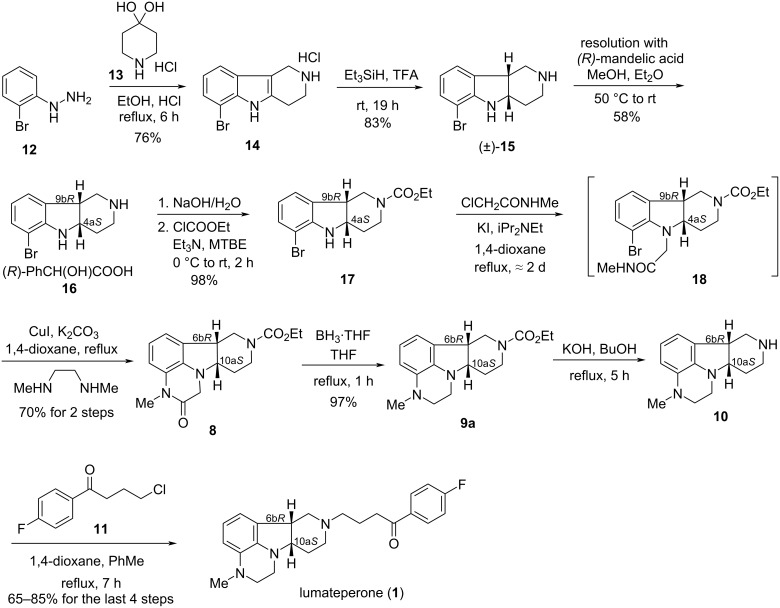
Alternate synthesis of lumateperone.

Later, alternate syntheses of lumateperone were described, also by the researchers of the originator company [8–10] (Scheme 2). The reaction of (2-bromophenyl)hydrazine (**12**) with 4-piperidone monohydrate hydrochloride (**13**) gave pyrido[4,3-*b*]indole congener **14**, which was reduced with triethylsilane in TFA to *cis*-racemate **15**. The key step of the syntheses is the resolution of this intermediate with (*R*)-mandelic acid. The enantiomerically pure (*R*)-mandelate salt **16** thus obtained was reacted with ethyl chloroformate to give **17**. It was then transformed to tetracyclic derivative **8** in various ways (we only show here the path that seems the most advantageous). According to this, compound **17** was *N*-alkylated at the indoline nitrogen atom with *N*-methylchloroacetamide to give **18**, then cyclized in one pot to derivative **8**. Alternate methods for the **17**→**8** transformation require more reaction steps [8–10], e.g., because of the use of chloroacetamide instead of *N*-methylchloroacetamide, necessitating an additional *N*-methylation. The tetracycle **8** was finally subjected to the same reaction sequence as the corresponding racemate (see also Scheme 1), lumateperone (**1**) was thus prepared via intermediates **9a** and **10**.

**Scheme 3:**
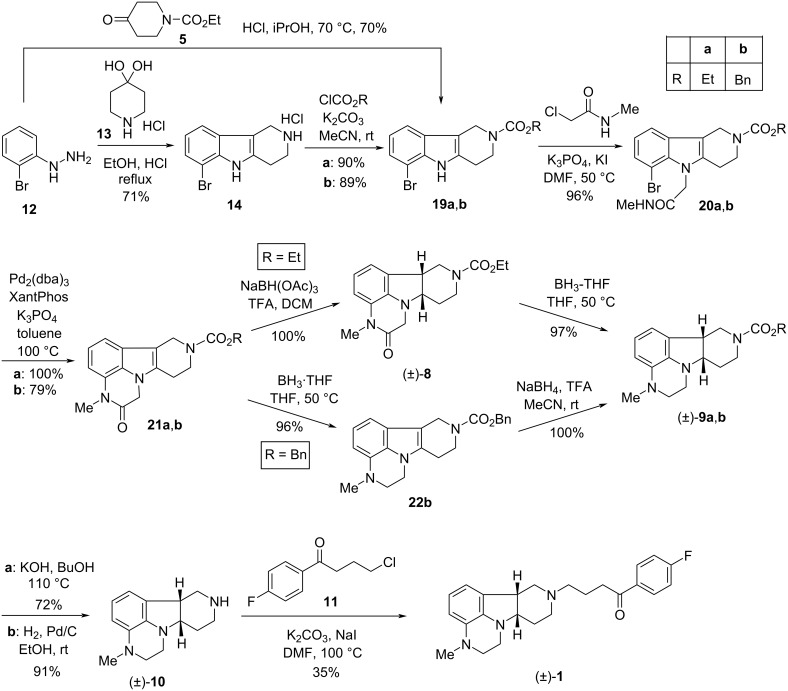
Alternate synthetic approaches leading to racemic lumateperone ((±)-**1**)).

Further synthetic variants resulting in racemic precursors (±)-**9a**,**b**, which were transformed to racemic lumateperone [(±)-**1**] via (±)-**10**, were also described [11–12] (Scheme 3). The main features of these syntheses are as follows: (i) in addition to the ethoxycarbonyl group (**19a**), a benzyloxycarbonyl group (**19b**) was also used for *N*-protection; (ii) compounds **19a**,**b** were *N*-alkylated at the indole nitrogen atom with *N*-methylchloroacetamide to derivatives **20a**,**b**; (iii) the **20**→**21** cyclization was realized by a Xantphos*/*Pd*-*catalyzed C–N bond-forming reaction; (iv) synthetic steps of the **21a**,**b***→*(±)-**9a**,**b** transformation were swapped when starting from **21a** compared to the reaction starting from **21b**. In the former case, the reduction of the double bond resulting in (±)-**8a** was followed by the reduction of the lactam moiety, while in the case of **21b** the reduction of the lactam carbonyl (**21b***→***22b**) preceded the reduction of the C–C double bond of **22b**. Both the hydrolytic desethoxycarbonylation of (±)-**9a** as well as the removal of the benzyloxycarbonyl group of (±)-**9b** by catalytic hydrogenation afforded (±)-**10** which was *N*-alkylated with 4-chloro-1-(4-fluorophenyl)butan-1-one (**11**) to give the racemic form of lumateperone [(±)-**1**]. It has to be mentioned that the resolution of none of the racemic intermediates of this synthetic route was disclosed [11–12].

## Results and Discussion

From the technological point of view, the obvious common disadvantage of the methods described above for the synthesis of lumateperone is the use of borane·THF complex for the reduction of the lactam carbonyl group, which may cause difficulties in industrial scale production (Schemes 1–3, step **8**→**9a** and Scheme 3, step **21b**→**22b**). Considering that we have developed a method and filed a patent application [13] for the resolution of compound (±)-**10**, a direct intermediate of lumateperone, easily available from (±)-**9a**, we aimed to elaborate a new, practical synthesis of the latter.

**Scheme 4:**
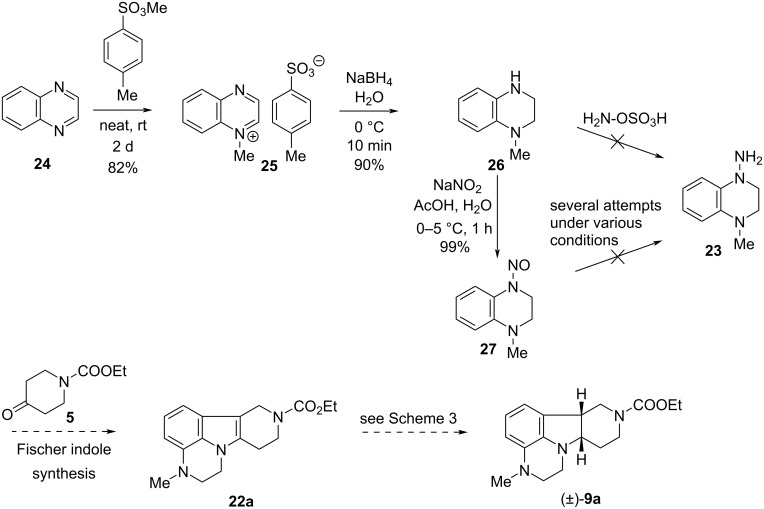
Planned new synthesis of key intermediate (±)-**9a**.

First, we envisaged a new synthetic route to the racemic key intermediate (±)-**9a**, significantly shorter than those described, which would have been based on the 1-methyl-4-amino-1,2,3,4-tetrahydroquinoxaline (**23**) intermediate (Scheme 4). We planned to convert the latter into (±)-**9a** via compound **22a** by known methods.

The synthesis of compound **23** was attempted as follows. *N*-Methylation of quinoxaline (**24**) with methyl *p*-toluenesulfonate followed by reduction of the *N*-methylated quaternary ammonium salt **25** with sodium borohydride gave tetrahydroquinoxaline **26**. Since *N*-amination of the latter with hydroxylamine-*O*-sulfonic acid [14] had been unsuccessful, we tried to achieve our target via the *N*-nitroso derivative **27**, which was obtained from compound **26** by a conventional procedure [7–8]. Several methods have then been tried to convert **27** to the *N*-amino derivative **23** (Zn dust, AcOH [8]; Zn dust, NH_4_Cl [15]; H_2_, Pd/C [16]; Na_2_S_2_O_4_ [17–18]; Mg, TiCl_4_ [19]), but to our regret, in all cases the *N*-nitroso group was removed and compound **26** was recovered instead of the expected product **23**.

**Scheme 5:**
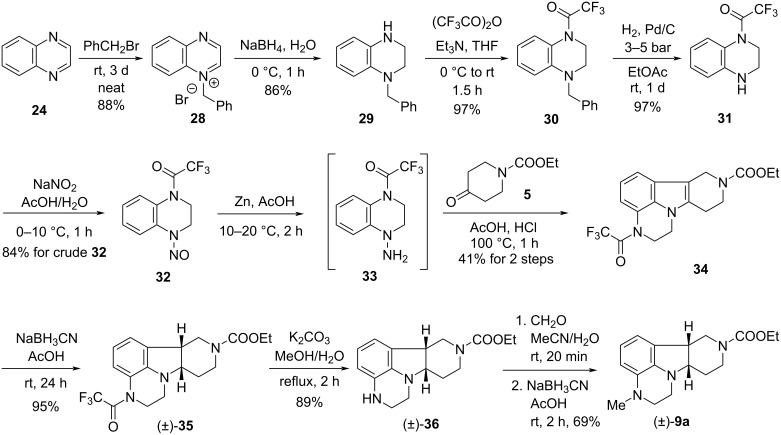
New synthesis of key intermediate (±)-**9a**.

So we were forced to go on a longer new way to key intermediate (±)-**9a**. Quinoxaline (**24**), the price of which is ca. 3% of that of 3,4-dihydroquinoxalin-2(1*H*)-one (**2**), remained our starting compound [20–23] (Scheme 5). *N*-Benzylation of **24** was accomplished in neat conditions within 3 days of reaction time using a literature procedure [5]. Attempts in acetonitrile as solvent were unsuccessful: after 1 day at room temperature, product **28** was isolated in only 20% yield, while at reflux temperature, a decomposition has been observed. Reduction of the quaternary ammonium salt **28** with sodium borohydride gave tetrahydroquinoxaline **29**. Its reaction with trifluoroacetic anhydride (TFAA) to give **30** and removal of the benzyl group by catalytic hydrogenation afforded *N*-trifluoroacetyl-1,2,3,4-tetrahydroquinoxaline (**31**). Compound **31** was then transformed into *N*-nitroso derivative **32** by treatment with sodium nitrite in aqueous acetic acid. The next two steps, reduction of the nitroso group to hydrazine derivative **33** and its coupling with piperidinone **5** in a Fischer indole synthesis, were telescoped to form tetracyclic product **34**. The latter was reduced with sodium cyanoborohydride in acetic acid to *cis*-indoline derivative (±)-**35**. Removal of the trifluoroacetyl group to (±)-**36** followed by *N*-methylation with formaldehyde and sodium cyanoborohydride gave target compound (±)-**9a**.

**Scheme 6:**
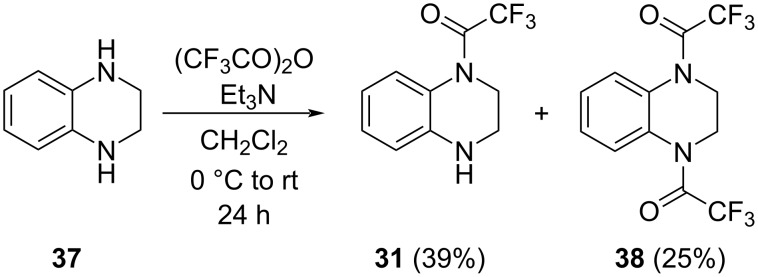
Trifluoroacetylation of tetrahydroquinoxaline (**37**).

It has to be mentioned that the preparation of compound **31** has been recently described in a patent application [24] by a direct trifluoroacetylation of tetrahydroquinoxaline (**37**) with 0.92 equiv of TFAA, followed by chromatographic product separation. We carried out the same reaction (Scheme 6) and in addition to the expected compound **31** (39%), a significant amount of bis(trifluoroacetyl) derivative **38** (25%) and starting material **37** (25%) were isolated as well (see Supporting Information File 1 for details). It has become clear that the formation of substantial amounts of the diacylated product **38** could not be avoided, thus turning the simple scaled up production of compound **31** unfeasible.

The main indicators of the synthetic approaches known from the literature and those of the new synthesis disclosed here are summarized in Table 1.

**Table 1:** Summary of known synthetic methods leading to tetracyclic compound **9a**.

No. of scheme	Starting material	Target product	Overall yield (%)	No. of isolated intermediates	Advantages	Disadvantages

Scheme 1	3,4-dihydroquinoxalin- 2(1*H*)-one (**2**)	(±)-**9a**	20	5	short synthesis	expensive starting material, BH_3_·THF reagent is sensitive to air and flammable, reduction in highly corrosive TFA solvent, low overall yield

Scheme 2	(2-bromophenyl)- hydrazine (**12**)	**9a**	24	6	synthesis of enantiopure product	expensive starting material, BH_3_·THF is sensitive to air and flammable, reduction in highly corrosive TFA solvent, low overall yield

Scheme 3a	(2-bromophenyl)- hydrazine (**12**)	(±)-**9a**	65/60	5/6	short synthesis, high overall yield	expensive starting material, BH_3_·THF is sensitive to air and flammable, reduction in highly corrosive TFA solvent

Scheme 3b	(2-bromophenyl)- hydrazine (**12**)	(±)-**9a**	46	6		

Scheme 5	quinoxaline (**24**)	(±)-**9a**	14	9	inexpensive starting material, avoidance of BH_3_·THF reagent and TFA solvent	use of protecting groups, higher number of steps, lower overall yield

## Conclusion

In summary, after the experimental study of various approaches, a new and efficient synthesis has been developed at laboratory scale for the synthesis of racemic key intermediate (±)-**9a** of the new drug lumateperone. The novel approach is based on the cheaply available starting material quinoxaline and leads to the tetracyclic compound via chemical steps characterized with good to excellent yields. The method described here does not require a reduction step carried out with borane·THF complex that is used in the syntheses described in the literature but would make it difficult to scale up the process. The significance of compound (±)-**9a** lies in the fact that it can be converted in a known one-step reaction to compound (±)-**10**. The resolution of the latter and the conversion of the suitable enantiomer to the drug lumateperone is disclosed in our patent application.

## Supporting Information

File 1 General information, synthetic procedures, and spectral data.
