# Supplementary material for: New synthesis of a late-stage tetracyclic key intermediate of lumateperone
Source: Beilstein J Org Chem. 2022 Jun 10;18:653–9. doi: 10.3762/bjoc.18.66 (PMC9194496; doi:10.3762/bjoc.18.66)
Supplement: File 1 — General information, synthetic procedures, and spectral data. [file Beilstein_J_Org_Chem-18-653-s001.pdf]

BEILSTEIN JOURNAL OF ORGANIC CHEMISTRY

Supporting Information

for

New synthesis of a late-stage tetracyclic key intermediate of lumateperone

Mátyás Milen, Bálint Nyulasi, Tamás Nagy, Gyula Simig and Balázs Volk

Beilstein J. Org. Chem. **2022,** *18,* 653–659. doi:10.3762/bjoc.18.66

General information, synthetic procedures, and spectral data

License and Terms: This is a supporting information file under the terms of the Creative Commons Attribution License (https://creativecommons.org/licenses/by/4.0). Please note that the reuse, redistribution and reproduction in particular requires that the author(s) and source are credited and that individual graphics may be subject to special legal provisions.
The license is subject to the *Beilstein Journal of Organic Chemistry* terms and conditions: (https://www.beilstein-journals.org/bjoc/terms)

Table of contents

1. General information..S2

2. Synthesis S3

3. References..S10

4. NMR and IR spectra S11

General information

All melting points were determined on a Büchi–Boëtius microapparatus and are uncorrected. IR spectra were obtained on a Bruker Alpha FT-IR spectrometer in KBr pellets. ^{1}H NMR and ^{13}C NMR spectra were recorded in DMSO-d_6 or $CDCl_3$ solution in 5 mm tubes at room temperature, on a Bruker Avance III HD 600 (600 and 150 MHz for ^{1}H and ^{13}C NMR spectra, respectively) or a Bruker Avance III 400 (400 and 100 MHz for ^{1}H and ^{13}C NMR spectra, respectively) spectrometer with the deuterium signal of the solvent as the lock and TMS as the internal standard. Chemical shifts (δ) and coupling constants (*J*) are given in ppm and in Hz, respectively. The following abbreviations are used to designate multiplicities: s = singlet, d = doublet, t = triplet, q = quartet, m = multiplet, br = broad. Mass spectra were recorded on a Bruker O-TOF MAXIS Impact mass spectrometer coupled to a Dionex Ultimate 3000 RS HPLC system with a diode array detector. High-resolution mass spectra (HRMS) were recorded on a Bruker O-TOF MAXIS Impact mass spectrometer coupled with a Waters I-Class UPLC system with a diode array detector. Reactions were monitored by thin-layer chromatography (TLC) carried out on silica gel plates (60 F_{254}) using UV light as visualizing agent. Purifications by flash column chromatography were carried out using Merck 107736 silica gel 60 H with a hexane/EtOAc or CH_2Cl_2/MeOH solvent system. All reagents were purchased from commercial sources and were used without further purification. Analytical samples of new compounds were obtained by recrystallization from the solvents or solvent mixtures given after the melting points in parentheses. Compounds (±)-**9**, **25**, **26**, **28**, **29**, **31**, (±)-**36**, and **38** are known from the literature, but insufficiently or not at all [(±)-**36**] characterized [1,2], therefore they are described below. New compounds **27**, **30**, **34**, and (±)-**35** are also characterized below in detail.

Synthesis

1-Methylquinoxalin-1-ium *p*-toluenesulfonate (25). This compound was prepared by a published method [3]. A mixture of quinoxaline (**24**, 10.0 g, 76.8 mmol) and methyl *p*-toluenesulfonate (27.17 g, 22.0 mL, 145.9 mmol, 1.9 equiv) was kept at room temperature for 2 d. The resulting solid was suspended in DEE (30 mL), filtered, and washed with DEE (10 mL). Yield: 19.93 g (82%). Colorless crystals. Mp 156–158 °C (EtOH–Et_2O, lit. mp 150–152 °C [4]). ^{1}H NMR ($CDCl_3$, 400 MHz): δ 9.69–9.65 (m, 1H), 9.61–9.56 (m, 1H), 8.64–8.58 (m, 1H), 8.54–8.48 (m, 1H), 8.36–8.26 (m, 2H), 7.46–7.41 (m, 2H), 7.10–7.04 (m, 2H), 4.73 (s, 3H), 2.27 (s, 3H) ppm. ^{13}C NMR ($CDCl_3$, 100 MHz): δ 148.2, 145.9, 145.0, 141.9, 137.7, 135.8, 133.8, 131.2, 131.0, 128.2, 125.6, 119.8, 45.8, 20.9 ppm.

1-Methyl-1,2,3,4-tetrahydroquinoxaline (26). This compound was prepared by a published method [3]. $NaBH_4$ (1.80 g, 47.6 mmol) was dissolved in H_2O (51 mL) and cooled to 0 °C. 1-Methylquinoxalin-1-ium *p*-toluenesulfonate (**25**, 5.00 g, 15.8 mmol) was dissolved in H_2O (10 mL) and added dropwise to the $NaBH_4$ solution over a period of 0.5 h. The mixture was stirred for an additional 10 min at 0 °C, then extracted with CH_2Cl_2 (3 × 30 mL). The combined organic layers were dried over $MgSO_4$, filtered, and the solvent was removed by rotary evaporation. The crude material was purified by Kugelrohr distillation at 145 °C (2 mbar), lit. bp 163–169 °C (20 Hg mm) [4]. Yield: 2.10 g (90%). Pale yellow oil. ^{1}H NMR ($CDCl_3$, 600 MHz): δ 6.68–6.65 (m, 1H), 6.65–6.60 (m, 2H), 6.48–6.45 (m, 1H), 3.48–3.46 (m, 2H), 3.27–3.25 (m, 2H), 2.68 (s, 3H) ppm. ^{13}C NMR ($CDCl_3$, 150 MHz): δ 136.2, 134.2, 118.7, 118.2, 113.5, 111.7, 49.9, 41.3, 39.1 ppm.

1-Methyl-4-nitroso-1,2,3,4-tetrahydroquinoxaline (27). The solution of **26** (9.40 g, 63.42 mmol) in AcOH (70.5 mL) and H_2O (47 mL) was cooled to 0–5 °C and treated dropwise with a solution of $NaNO_2$ (4.60 g, 66.67 mmol, 1.05 equiv) in H_2O (47 mL) at the same temperature. After stirring for 1 h at 0–5 °C, the mixture was basified with 15% NH_3 solution

to pH ≈ 8 and extracted with CH_2Cl_2 (3 × 50 mL), dried over Na_2SO_4, filtered, and evaporated under reduced pressure. Yield: 11.20 g (99%). Yellow oil. IR (film, cm^{-1}): 1605, 1503, 1425, 1306, 1170, 1131, 746. ^1H NMR ($CDCl_3$, 600 MHz): δ 8.04–8.02 (m, 1H), 7.24–7.20 (m, 1H), 6.89–6.85 (m, 1H), 6.80–6.78 (m, 1H), 4.06 (t, J = 5.6 Hz, 2H), 3.22 (t, J = 5.6 Hz, 2H), 2.93 (s, 3H) ppm. ^{13}C NMR ($CDCl_3$, 150 MHz): δ 137.2, 127.5, 126.2, 119.0, 115.7, 113.3, 48.2, 10.9, 39.1 ppm. HRMS calcd. for $C_9H_{12}N_3O$ $[M+H]^+$ 178.0975; found 178.0967.

1-Benzylquinoxalin-1-ium bromide (28). This compound was prepared by a published method [5]. The mixture of quinoxaline (**24**, 42.6 g, 327 mmol) and benzyl bromide (106.2 g, 73.8 mL, 621 mmol, 1.9 equiv) was left standing for 3 days. The resulting solid was filtered and washed extensively with DEE and dried. Yield: 87.1 g (88%). Yellow crystals. Mp 143–146 °C. IR (KBr, cm^{-1}): 3424, 1604, 1511, 1358, 1154, 772, 699. ^1H NMR ($CDCl_3$, 400 MHz): δ 10.73 (d, J = 2.9 Hz, 1H), 9.63 (d, J = 2.8 Hz, 1H), 8.76 (d, J = 8.8 Hz, 1H), 8.44 (dd, J_1 = 1.6 Hz, J_2 = 8.4 Hz, 1H), 8.20–8.16 (m, 1H), 8.13–8.08 (m, 1H), 7.61–7.58 (m, 2H), 7.33–7.31 (m, 3H), 6.91 (s, 2H) ppm. ^{13}C NMR ($CDCl_3$, 100 MHz): δ 147.6, 146.1, 140.3, 136.3, 133.5, 132.1, 131.5, 130.1, 129.8, 129.6, 128.5, 119.7, 61.4 ppm.

1-Benzyl-1,2,3,4-tetrahydroquinoxaline (29). An analogous method for the *N*-methyl derivative was described by Beach and coworkers [3]. 1-Benzylquinoxalin-1-ium bromide (**28**, 30.0 g, 99.6 mmol) was dissolved in H_2O (300 mL). To a solution of $NaBH_4$ (11.40 g, 301.3 mmol, 3.0 equiv) in H_2O (150 mL) was added the aqueous solution of **28** over a period of 30 min at 0 °C. The mixture was stirred for further 30 min at 0 °C and then extracted with DCM (3 × 60 mL). The combined organic layers were washed with H_2O (2 × 25 mL), dried over $MgSO_4$, filtered, and the filtrate was concentrated under reduced pressure to afford the title compound **29**. Yield: 22.34 g (86%). Pale yellow crystals. Mp 52–53 °C (hexane, lit. mp 50.5–52.5 °C [6]). ^1H NMR ($CDCl_3$, 400 MHz): δ 7.33–7.29 (m, 4H), 7.26–7.22 (m, 1H), 6.63–6.57 (m, 1H), 6.56–6.52 (m, 3H), 4.44 (s, 2H), 3.73 (br s, 1H), 3.44–3.40 (m, 4H) ppm. ^{13}C NMR

($CDCl_3$, 100 MHz): δ 138.7, 135.4, 133.8, 128.5, 127.0, 126.8, 119.2, 117.6, 114.1, 111.8, 55.2, 48.4, 41.0 ppm [7].

1-Benzyl-4-(trifluoroacetyl)-1,2,3,4-tetrahydroquinoxaline (30). To a stirred solution of amine **29** (8.60 g, 38.34 mmol) and TEA (8.0 mL, 5.81 g, 57.42 mmol, 1.5 equiv) in THF (43 mL) was carefully added TFAA (8.10 mL, 12.06 g, 57.42 mmol, 1.5 equiv) at 0 °C. The reaction mixture was stirred for 30 min at the same temperature and for further 60 min at 25 °C. The mixture was then evaporated under reduced pressure. The residue was dissolved in DCM (50 mL), washed with H_2O (2 × 20 mL), dried over Na_2SO_4, filtered, and evaporated to give the title compound **30**. Yield: 11.95 g (97%). Colorless crystals. Mp 91–92 °C (^{i}PrOH). IR (KBr, cm^{-1}): 3440, 2873, 1688, 1604, 1511, 1350, 1204, 1189, 1146, 751. ^{1}H NMR (DMSO-d_6, 600 MHz): 7.51–7.47 (m, 1H), 7.35–7.31 (m, 2H), 7.28–7.21 (m, 3H), 7.02–6.98 (m, 1H), 6.73–6.68 (m, 1H), 6.62–6.58 (m, 1H), 4.58 (s, 2H), 3.98 (s, 2H), 3.60–3.53 (m, 2H) ppm. ^{13}C NMR (DMSO-d_6, 150 MHz): δ 153.6 (q, J = 35.1 Hz), 139.2, 138.0, 128.8, 127.6, 127.1, 126.8, 124.6, 121.9, 116.6 (q, J = 289.2 Hz), 115.1, 112.2, 53.5, 49.4, 43.2 ppm. At room temperature, the compound consists of a ca. 9:1 mixture of two rotamers. Only the signals of the major species are given here. HRMS calcd. for $C_{17}H_{16}F_3N_2O$ [M+H]$^+$ 321.1215; found 321.1212.

1-(Trifluoroacetyl)-1,2,3,4-tetrahydroquinoxaline (31). _Method A:_ To a stirred solution of **30** (2.00 g, 6.24 mmol) in EtOAc (80 mL) were added a saturated HCl solution in EtOAc (1.0 mL) and palladium on carbon (10% Pd, 0.20 g). The reaction mixture was stirred under hydrogen atmosphere (3–5 bar) in an autoclave for 1 d. The catalyst was filtered off and the filtrate was evaporated to dryness. The waxy solid was treated with hexane (5 mL), the crystals were filtered, washed with MTBE (2 × 3 mL) and dried to give title compound **30**. Yield: 1.40 g (97%). Colorless crystals. Mp 162–164 °C (MeCN). IR (KBr, cm^{-1}): 3389, 1674, 1605, 1515, 1283, 1188, 1142, 756. ^{1}H NMR (DMSO-d_6, 600 MHz): 7.49–7.46 (m, 1H), 7.04–6.94 (m, 1H), 6.66–6.63 (m, 1H), 6.53–6.49 (m, 1H), 6.35 (br s, 1H), 3.80 (br s, 2H), 3.42 (br s, 2H) ppm. ^{13}C

NMR (DMSO-d_6, 150 MHz): δ 153.6 (q, J = 34.8 Hz), 139.2, 127.2, 124.4, 120.8, 116.6 (q, J = 288.9 Hz), 114.7, 114.3, 43.0, 41.6 ppm. At room temperature, the compound consists of a ca. 9:1 mixture of two rotamers. Only the signals of the major species are given here. HRMS calcd. for $C_{10}H_{10}F_3N_2O$ $[M+H]^+$ 231.0745; found 231.0742. *Method B:* According to a published method [8], trifluoroacetic anhydride (1.30 mL, 1.94 g, 9.2 mmol) was added dropwise to a solution of 1,2,3,4-tetrahydroquinoxaline (**37**, 1.34 g, 10.0 mmol) and Et_3N (1.53 mL, 1.11 g, 11.0 mmol) in CH_2Cl_2 (10 mL) at 0 °C. After 30 min, the reaction temperature was allowed to warm to 23 °C and the mixture was stirred overnight and evaporated. The residue was separated by flash column chromatography (SiO_2, hexane→hexane–EtOAc 50:50) to afford three compounds: 0.34 g (25%) of the starting material, 0.90 g (39%) of title compound **31** and 0.58 g (25%) of 1,4-bis-(trifluoroacetyl)-1,2,3,4-tetrahydroquinoxaline (**38**). For analytical data of compound **38**, see below.

Ethyl 3-(trifluoroacetyl)-2,3,9,10-tetrahydro-1*H*-pyrido[3',4':4,5]pyrrolo[1,2,3-*de*]quinoxaline-8(7*H*)-carboxylate (34). The solution of **31** (5.10 g, 22.16 mmol) in AcOH (100 mL) and H_2O (50 mL) was cooled to 5–10 °C and treated dropwise with a solution of sodium nitrite (1.80 g, 26.09 mmol, 1.18 equiv) in H_2O (30 mL) at the same temperature. After stirring for 1 h at 0–5 °C, the product was filtered, washed with H_2O (2 × 5 mL) and dried. Yield: 4.80 g (84%). This nitroso intermediate (**32**) was directly used in the next reaction according to the procedure described in the literature for a similar reaction [1]. To a stirred solution of **32** (4.80 g, 18.52 mmol) in AcOH (80 mL) at 10–12 °C was added freshly activated zinc powder [9] (6.40 g, 97.89 mmol, 5.5 equiv) portionwise. The reaction mixture was stirred at 10–20 °C for 2 h. Ethyl 4-oxopiperidine-1-carboxylate (**5**, 2.80 mL, 3.18 g, 18.52 mmol) was added and the mixture was filtered after 5 min of stirring. The insoluble material was washed with acetic acid (4 × 16 mL). To the combined filtrates, a saturated HCl solution in DEE (0.80 mL) was added and heated to 100 °C for 1 h. The dark reaction mixture was cooled and

evaporated under reduced pressure. Flash column chromatography (SiO_2, CH_2Cl_2 → CH_2Cl_2/MeOH 92:8) afforded the desired product. Yield: 2.89 g (41%). Colorless crystals. Mp 126–128 °C (MeCN). IR (KBr, cm^{-1}): 2970, 1699, 1578, 1386, 1199, 1056. ^{1}H NMR ($CDCl_3$, 400 MHz): 7.83 (br s, 1H), 7.34–7.26 (m, 1H), 7.12–7.08 (m, 1H), 4.71 (s, 2H), 4.23–4.18 (m, 6H), 3.89 (s, 2H), 2.82 (s, 2H) 1.31 (t, J = 7.2 Hz, 3H) ppm. ^{13}C NMR ($CDCl_3$, 100 MHz): δ 156.0, 154.8 (q, J = 35.0 Hz), 132.4, 127.3, 124.4, 122.7, 119.6, 116.3 (q, J = 288.3 Hz), 115.8, 114.0, 107.7, 61.6, 44.6, 41.9, 41.3, 40.9, 21.6, 14.7 ppm. At room temperature, the compound consists of a ca. 2:1 mixture of two rotamers. Only the signals of the major species are given here. HRMS calcd. for $C_{18}H_{19}F_3N_3O_3$ $[M+H]^+$ 382.1379; found 382.1372.

***cis*-Ethyl 3-(trifluoroacetyl)-2,3,6b,9,10,10a-hexahydro-1*H*-pyrido[3',4':4,5]pyrrolo[1,2,3-*de*]quinoxaline-8(7*H*)-carboxylate [(±)-35].** A stirred solution of **34** (1.00 g, 2.62 mmol) in AcOH (58 mL) was cooled to 17 °C. The solution was treated portionwise with $NaBH_3CN$ (492 mg, 7.83 mmol, 3.0 equiv) and stirred at room temperature for 24 h. The reaction mixture was diluted with H_2O (87 mL) and basified with 25% NH_4OH (ca. 70 mL) up to pH 8. The precipitated product was filtered, washed with H_2O (2 × 5 mL) and dried. Yield: 960 mg (95%). Colorless crystals. Mp 149–152 °C (^{i}Pr_2O, decomp.). IR (KBr, cm^{-1}): 1685, 1202, 1186, 1142, 1121. ^{1}H NMR (DMSO-d_6, 600 MHz): 7.86–7.84 (m, 1H), 7.01–7.00 (m, 1H), 6.66–6.63 (m, 1H), 4.08–4.06 (m, 1H), 4.04–4.01 (m, 2H), 4.00–3.97 (m, 1H), 3.87–3.77 (m, 1H), 3.67–3.64 (m, 1H), 3.54–3.46 (m, 2H), 3.28–3.24 (m, 1H), 3.22–3.17 (m, 2H), 3.07–3.04 (m, 1H), 1.86–1.79 (m, 2H), 1.17 (t, J = 6.7 Hz, 3H) ppm. ^{13}C NMR (DMSO-d_6, 150 MHz): 154.9, 154.0 (q, J = 34.8 Hz), 142.0, 130.5, 121.9, 121.6, 120.5, 118.0, 116.4 (q, J = 288.5 Hz), 62.8, 60.8, 44.9, 43.9, 42.7, 40.1, 39.3, 23.6/23.5, 14.8 ppm. At room temperature, the compound consists of a ca. 1:1 mixture of two rotamers. HRMS calcd. for $C_{18}H_{21}F_3N_3O_3$ $[M+H]^+$ 384.1535; found 384.1529.

***cis*-Ethyl 2,3,6b,9,10,10a-hexahydro-1*H*-pyrido[3',4':4,5]pyrrolo[1,2,3-*de*]quinoxaline-8(7*H*)-carboxylate [(±)-36]** [1,2]. To a solution of (±)-**35** (500 mg, 1.30 mmol) in MeOH (20 mL) and H_2O (0.8 mL) was added K_2CO_3 (0.36 g, 2.61 mmol, 2.0 equiv). The reaction mixture was stirred at reflux temperature for 2 h and evaporated. The residue was dissolved in the mixture of EtOAc (20 mL) and H_2O (20 mL). After separation, the aqueous phase was extracted with EtOAc (2 × 20 mL). The combined organic layers were washed with brine (20 mL), dried over $MgSO_4$, filtered, and evaporated under reduced pressure to provide the product. Yield: 334 mg (89%). Off-white crystals. Mp 122–125 °C (hexane). IR (KBr, cm^{-1}): 3342, 1678, 1483, 1440, 1122, 730. ^1H NMR ($CDCl_3$, 600 MHz): 6.61–6.59 (m, 1H), 6.58–6.56 (m, 1H), 6.41–6.39 (m, 1H), 4.20–4.11 (m, 2H), 4.16/4.05–3.98 (m, 1H), 3.94–3.88/3.88–3.83 (m, 1H), 3.72–3.67 (m, 1H), 3.60 (br s, 1H), 3.51–3.47 (m, 1H), 3.36–3.34 (m, 1H), 3.33–3.31 (m, 1H), 3.22–3.15 (m, 1H), 3.14–3.07 (m, 1H), 2.92–2.86/2.86–2.78 (m, 1H), 2.72–2.67 (m, 1H), 1.95–1.90 (m, 1H), 1.89–1.82 (m, 1H), 1.28 (t, J = 7.1 Hz, 3H) ppm. ^{13}C NMR ($CDCl_3$, 150 MHz): δ 155.5, 138.0, 132.5, 129.7 (br), 120.3, 113.6 (br), 111.9, 65.3, 61.2, 45.6, 44.2, 42.3, 41.0/40.8, 39.7, 24.7/24.4, 14.7 ppm. At room temperature, the compound consists of a ca. 1:1 mixture of two rotamers.

1,4-Bis(trifluoroacetyl)-1,2,3,4-tetrahydroquinoxaline (38). This compound was obtained as a byproduct in the synthesis of compound **31** (see above). Colorless crystals. Mp 121–122.5 °C (EtOH, lit. mp 118–118.5 °C [10]). IR (KBr, cm^{-1}): 1689, 1505, 1206, 1185, 1140, 529. ^1H NMR ($CDCl_3$, 600 MHz): 8.00–7.60 (m, 1H), 7.60–7.30 (m, 3H), 4.18–4.02 (m, 4H) ppm. ^{13}C NMR ($CDCl_3$, 150 MHz): δ 156.0, 133.6/128.6, 128.6/127.2, 124.7, 116.0 (q, J = 288.4 Hz), 45.7 ppm. At room temperature, the compound consists of a ca. 1:1 mixture of two rotamers.

***cis*-Ethyl 3-methyl-2,3,6b,9,10,10a-hexahydro-1*H*-pyrido[3',4':4,5]pyrrolo[1,2,3-*de*]quinoxaline-8(7*H*)-carboxylate [(±)-9].** To a solution of (±)-**36** (320 mg, 1.11 mmol) in MeCN (26 mL) was added formaldehyde (0.83 mL of a 37% aqueous solution, 11.12 mmol,

10 equiv). After 20 min, to the dark orange reaction mixture were added $NaBH_3CN$ (176 mg, 2.80 mmol, 2.5 equiv) and AcOH (160 µL, 168 mg, 2.80 mmol, 2.5 equiv) and the mixture was stirred at room temperature for 2 h. The solvents were evaporated, and the residue was taken up in DCM (20 mL) and extracted with saturated $NaHCO_3$ (2 × 15 mL), dried over $MgSO_4$, filtered, and concentrated under reduced pressure to give the product. Yield: 232 mg (69%). Colorless oil. ^{1}H NMR ($CDCl_3$, 600 MHz): 6.68–6.65 (m, 1H), 6.56–6.54 (m, 1H), 6.42–6.41 (m, 1H), 4.17–4.13 (m, 2H), 4.04–3.98 (m, 1H), 3.93–3.81 (m, 1H), 3.62–3.58 (m, 1H), 3.35–3.32 (m, 2H), 3.30–3.26 (m, 1H), 3.19–3.11 (m, 2H), 2.87 (s, 3H), 2.86–2.84 (m, 1H), 2.82–2.78 (m, 1H), 1.92–1.82 (m, 2H) 1.28 (t, 3H, J = 7.2 Hz) ppm. ^{13}C NMR ($CDCl_3$, 150 MHz): δ 155.5, 138.0, 135.0, 128.6 (br), 120.4, 112.9 (br), 109.1, 64.9, 61.2, 50.5, 45.6, 44.2, 41.3/41.0, 39.7, 37.5, 24.6/24.3, 14.7 ppm. At room temperature, the compound consists of a ca. 1:1 mixture of two rotamers.

References

1. Robichaud, A. J.; Lee, T.; Deng, W.; Mitchell, I. S.; Yang, M. G.; Haydar, S.; Chen, W.; MC Clung, Ch. D.; Calvello, E. J. B.; Zawrotny, D. M. Preparation of substituted heterocycle fused gamma-carbolines. WO Patent 2000077002, December 21, 2000.

2. Li, P.; Zhang, Q.; Robichaud, A. J.; Lee, T.; Tomesch, J.; Yao, W.; Beard, J. D.; Snyder, G. L.; Zhu, H.; Peng, Y.; Hendrick, J. P.; Vanover, K. E.; Davis, R. E.; Mates, S.; Wennogle, L. P. *J. Med. Chem.* **2014**, *57*, 2670–2682. doi: 10.1021/jm401958n

3. Smith, R. F.; Rebel, W. J.; Beach, T. N. *J. Org. Chem*. **1959**, *24*, 205–207. doi: 10.1021/jo01084a014

4. Process for preparation of 1-(4-fluorophenyl)-4-((6b*R*,10a*S*)-3-methyl-2,3,6b,9,10,10a-hexahydro-1*H*-pyrido[3',4':4,5]pyrrolo[1,2,3-*de*]quinoxalin-8(7*H*)-yl)butan-1-one. IPCOM000258304D; *Chem. Abstr.* **2019**, *171*, 163843.

5. Leblanc, N.; Sproules, S.; Powell, A. K. *New J. Chem.* **2017**, *41*, 2949–2954. doi: 10.1039/C7NJ00531H

6. Cavagnol, J. C.; Wiselogle, F. Y. *J. Am. Chem. Soc*. **1947**, *69*, 795–799. doi: 10.1021/ja01196a015

7. Ogata, O.; Nara, H.; Matsumara, K.; Kayaki, Y. *Org. Lett*. **2019**, *10*, 9954–9959. doi: 10.1021/acs.orglett.9b03878

8. Murphy, B. P.; Zhang, G.; Zhao, J. Stable violet-blue to blue imidazolium compounds. WO Patent 2018052725, March 22, 2018.

9. Furniss, B. S.; Hannaford, A. J.; Smith, P. W. G.; Tatchell, A. R. *Vogel’s Textbook of Practical Organic Chemistry*, 5th ed.; Longman, Harlow, UK, 1989; pp 467.

10. De Selms, R. C.; Greaves, R. J.; Schleigh, W. R. *J. Heterocycl. Chem*. **1974**, *11*, 595–597. doi: 10.1002/jhet.5570110425

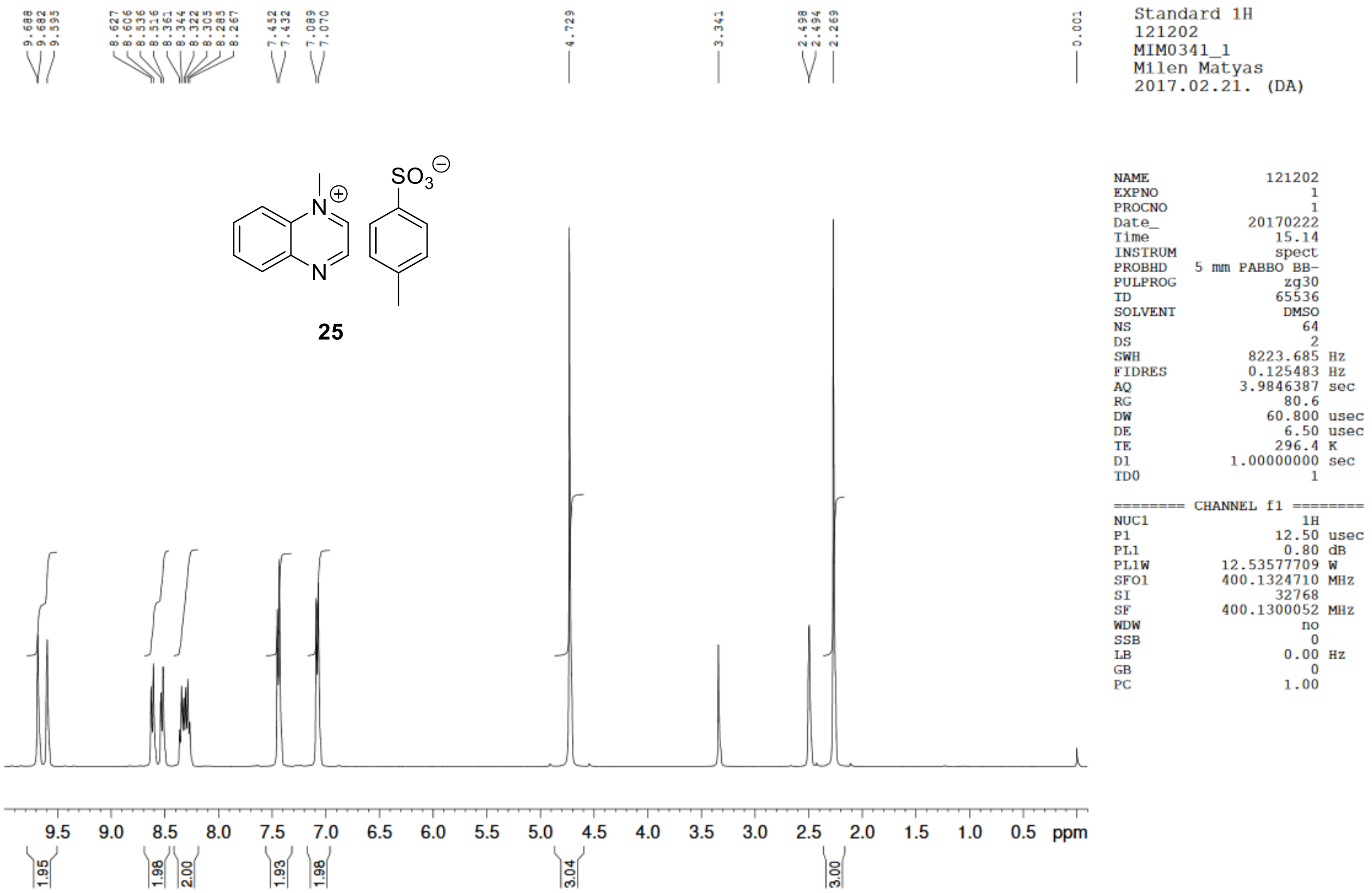
9.688
9.682
9.595
8.627
8.606
8.536
8.516
8.361
8.344
8.322
8.305
8.285
8.267
7.452
7.432
7.089
7.070
4.729
3.341
2.498
2.494
2.269
0.001
Standard 1H
121202
MIM0341_1
Milen Matyas
2017.02.21. (DA)
25
NAME 121202
EXPNO 1
PROCNO 1
Date_ 20170222
Time 15.14
INSTRUM spect
PROBHD 5 mm PABBO BB-
PULPROG zg30
TD 65536
SOLVENT DMSO
NS 64
DS 2
SWH 8223.685 Hz
FIDRES 0.125483 Hz
AQ 3.9846387 sec
RG 80.6
DW 60.800 usec
DE 6.50 usec
TE 296.4 K
D1 1.00000000 sec
TD0 1
======== CHANNEL f1 ========
NUC1 1H
P1 12.50 usec
PL1 0.80 dB
PL1W 12.53577709 W
SFO1 400.1324710 MHz
SI 32768
SF 400.1300052 MHz
WDW no
SSB 0
LB 0.00 Hz
GB 0
PC 1.00
9.5 9.0 8.5 8.0 7.5 7.0 6.5 6.0 5.5 5.0 4.5 4.0 3.5 3.0 2.5 2.0 1.5 1.0 0.5 ppm
1.95
1.98
2.00
1.93
1.98
3.04
3.00

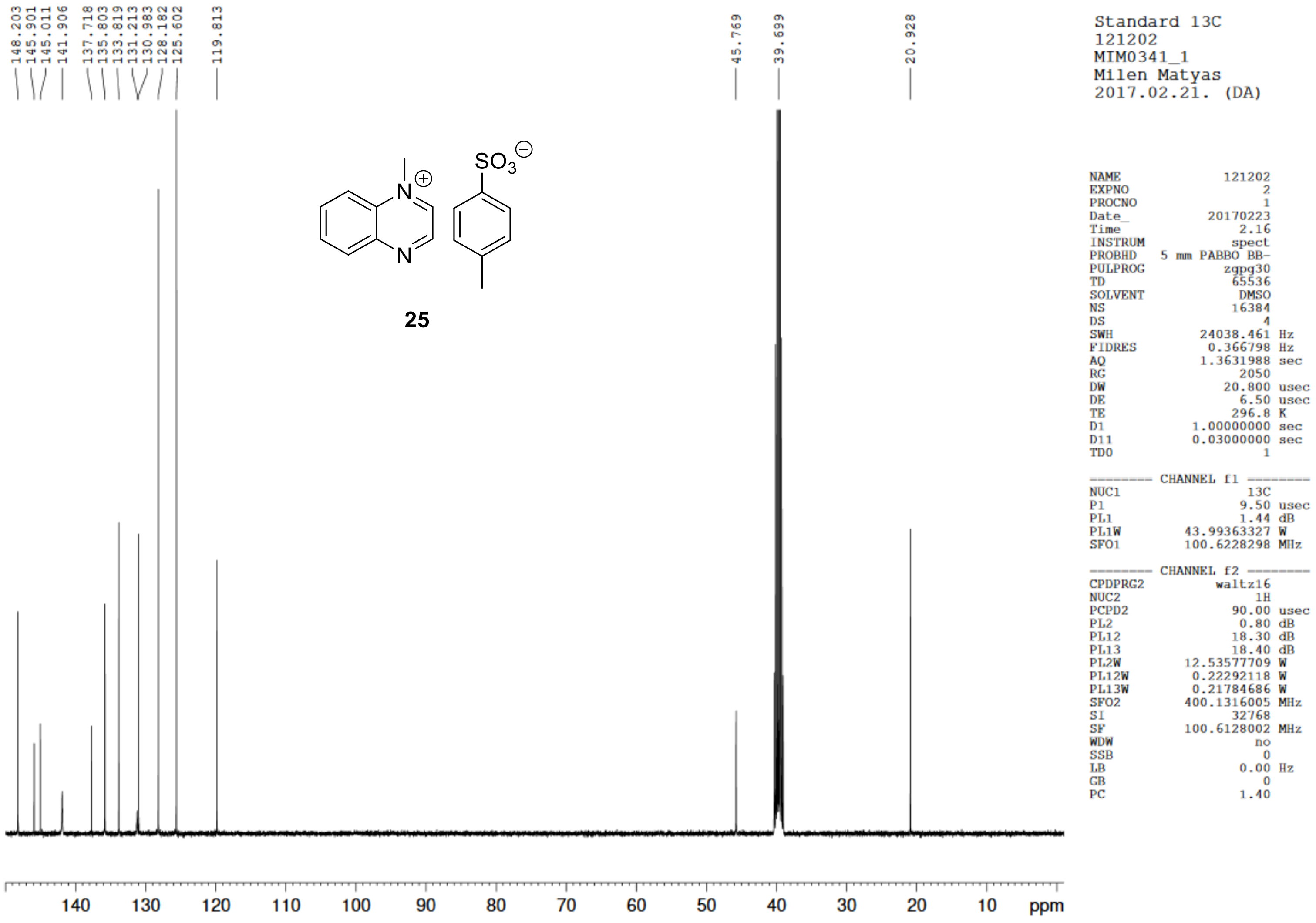
148.203
145.901
145.011
141.906
137.718
135.803
133.819
131.213
130.983
128.182
125.602
119.813
45.769
39.699
20.928
25
140 130 120 110 100 90 80 70 60 50 40 30 20 10 ppm
Standard 13C
121202
MIM0341_1
Milen Matyas
2017.02.21. (DA)
NAME 121202
EXPNO 2
PROCNO 1
Date_ 20170223
Time 2.16
INSTRUM spect
PROBHD 5 mm PABBO BB-
PULPROG zgpg30
TD 65536
SOLVENT DMSO
NS 16384
DS 4
SWH 24038.461 Hz
FIDRES 0.366798 Hz
AQ 1.3631988 sec
RG 2050
DW 20.800 usec
DE 6.50 usec
TE 296.8 K
D1 1.00000000 sec
D11 0.03000000 sec
TD0 1
======== CHANNEL f1 ========
NUC1 13C
P1 9.50 usec
PL1 1.44 dB
PL1W 43.99363327 W
SFO1 100.6228298 MHz
======== CHANNEL f2 ========
CPDPRG2 waltz16
NUC2 1H
PCPD2 90.00 usec
PL2 0.80 dB
PL12 18.30 dB
PL13 18.40 dB
PL2W 12.53577709 W
PL12W 0.22292118 W
PL13W 0.21784686 W
SFO2 400.1316005 MHz
SI 32768
SF 100.6128002 MHz
WDW no
SSB 0
LB 0.00 Hz
GB 0
PC 1.40

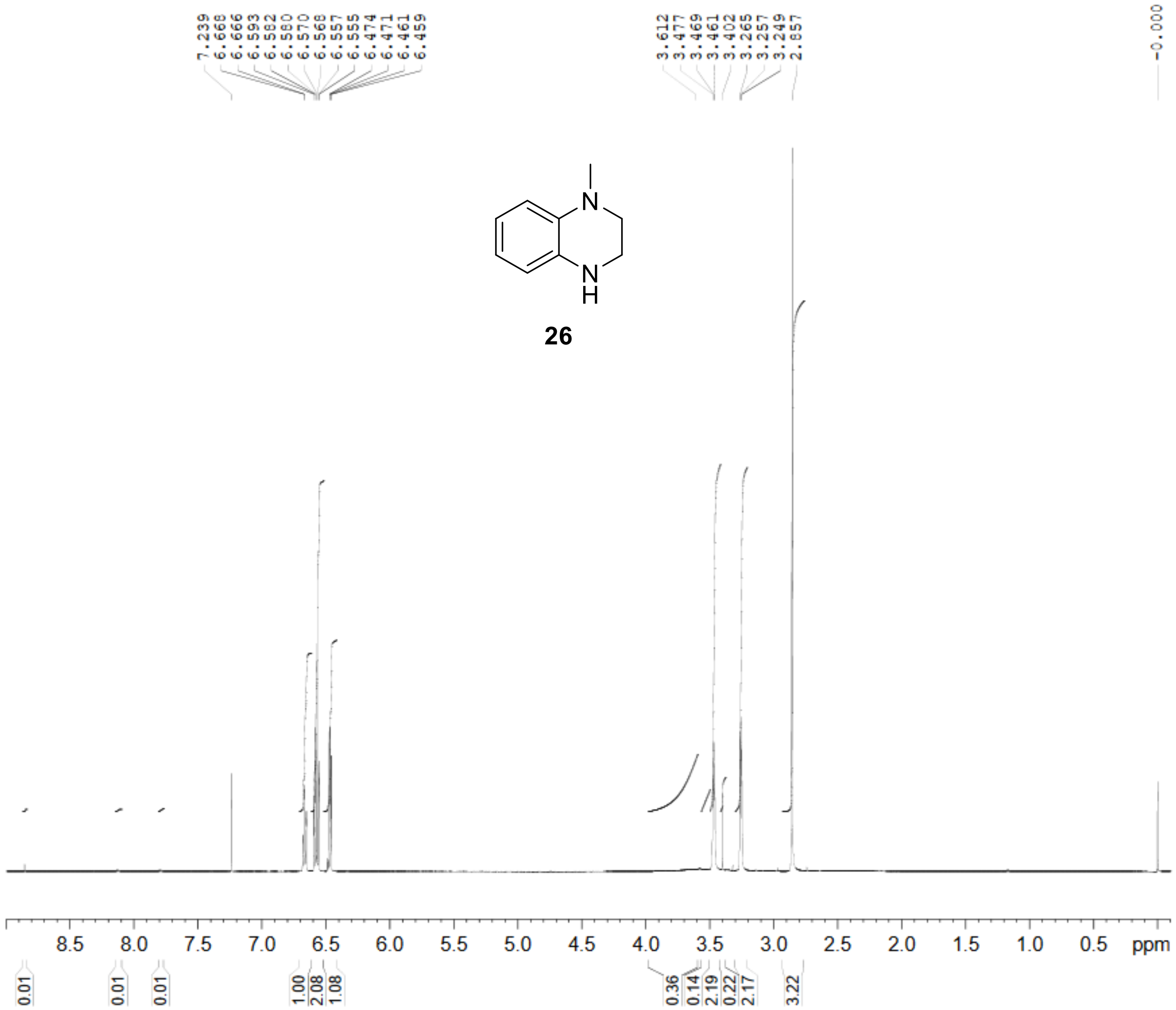


```
Standard 1H
121210
MIM0343_1
Milen Matyas
2017.02.22. (DA)

Current Data Parameters
NAME              121210
EXPNO                 11
PROCNO                 1

F2 - Acquisition Parameters
Date_           20170223
Time                1.59
INSTRUM            spect
PROBHD   5 mm CPPBBO BB
PULPROG             zg30
TD                 65536
SOLVENT            CDCl3
NS                    16
DS                     4
SWH           12626.263 Hz
FIDRES         0.192661 Hz
AQ            2.5952256 sec
RG                 89.22
DW                39.600 usec
DE                 25.00 usec
TE                 295.0 K
D1           1.00000000 sec
TD0                    1

======== CHANNEL f1 ========
SFO1        600.1642011 MHz
NUC1                  1H
P1                 11.50 usec
PLW1         28.00000000 W

F2 - Processing parameters
SI                 65536
SF          600.1600263 MHz
WDW                   no
SSB      0
LB       0 Hz
GB       0
PC                  1.00
```

BRUKER

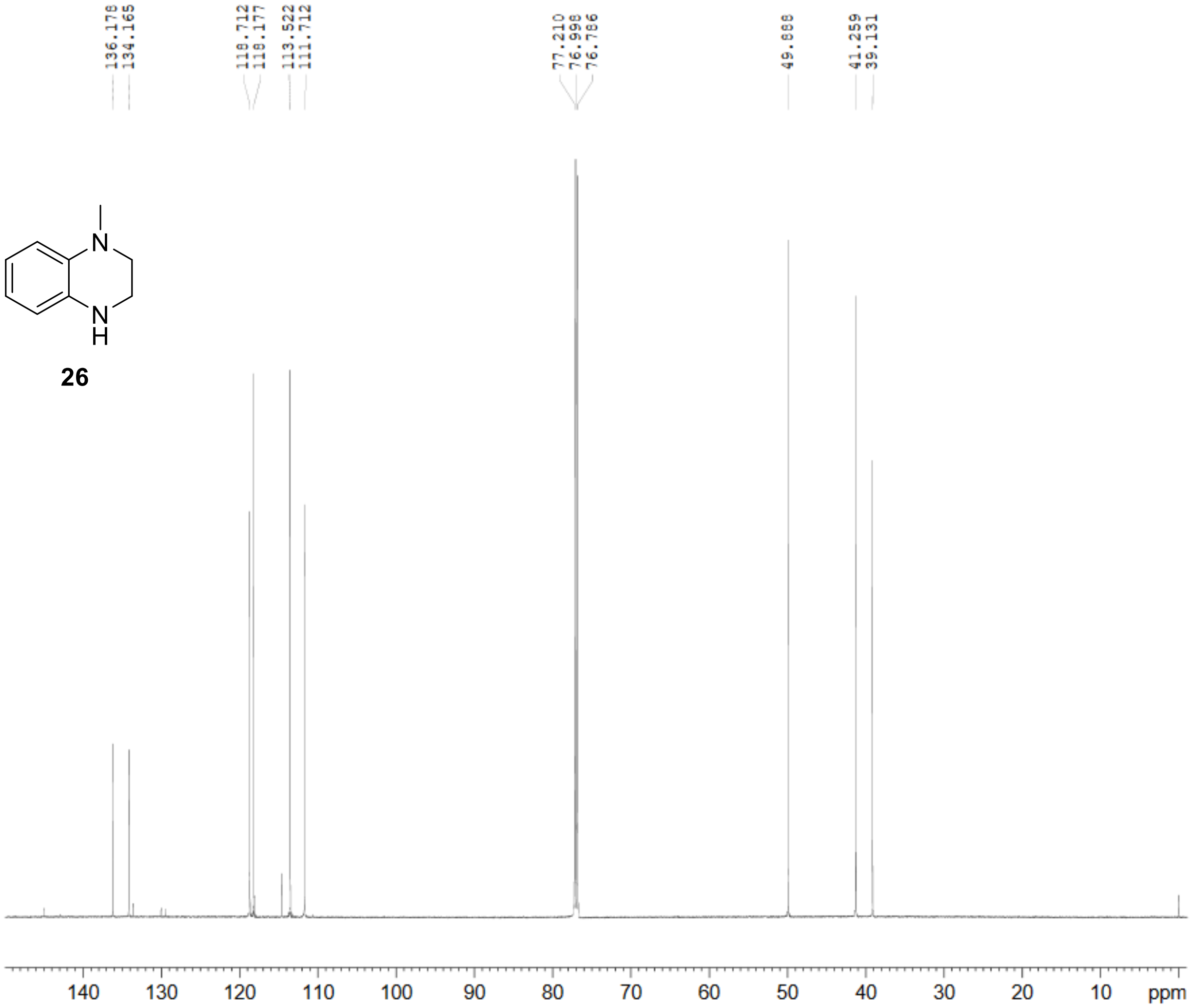

26

Standard 13C
121210
MIM0343_1
Milen Matyas
2017.02.22. (DA)

```
Current Data Parameters
NAME              121210
EXPNO                 12
PROCNO                 1

F2 - Acquisition Parameters
Date_           20170223
Time                2.43
INSTRUM            spect
PROBHD   5 mm CPPBBO BB
PULPROG           zgpg30
TD                 65536
SOLVENT            CDCl3
NS                  1024
DS                     4
SWH            36057.691 Hz
FIDRES          0.550197 Hz
AQ             0.9087659 sec
RG                196.07
DW                13.867 usec
DE                 18.00 usec
TE                 295.0 K
D1            1.50000000 sec
D11           0.03000000 sec
TD0                    1

======== CHANNEL f1 ========
SFO1         150.9254424 MHz
NUC1                 13C
P1                 10.00 usec
PLW1         65.00000000 W

======== CHANNEL f2 ========
SFO2         600.1624006 MHz
NUC2                  1H
CPDPRG[2         waltz16
PCPD2              70.00 usec
PLW2         28.00000000 W
PLW12         0.75571001 W
PLW13         0.37029999 W

F2 - Processing parameters
SI                 65536
SF           150.9103658 MHz
WDW                   EM
SSB       0
LB                  1.00 Hz
GB        0
PC                  1.40
```

**135068**	Milen Matyas	KP
**MIM0353_1**	Film	2020.10.28.

BRUKER Alpha
Resolution: 2 cm-1
Number of Scans: 16

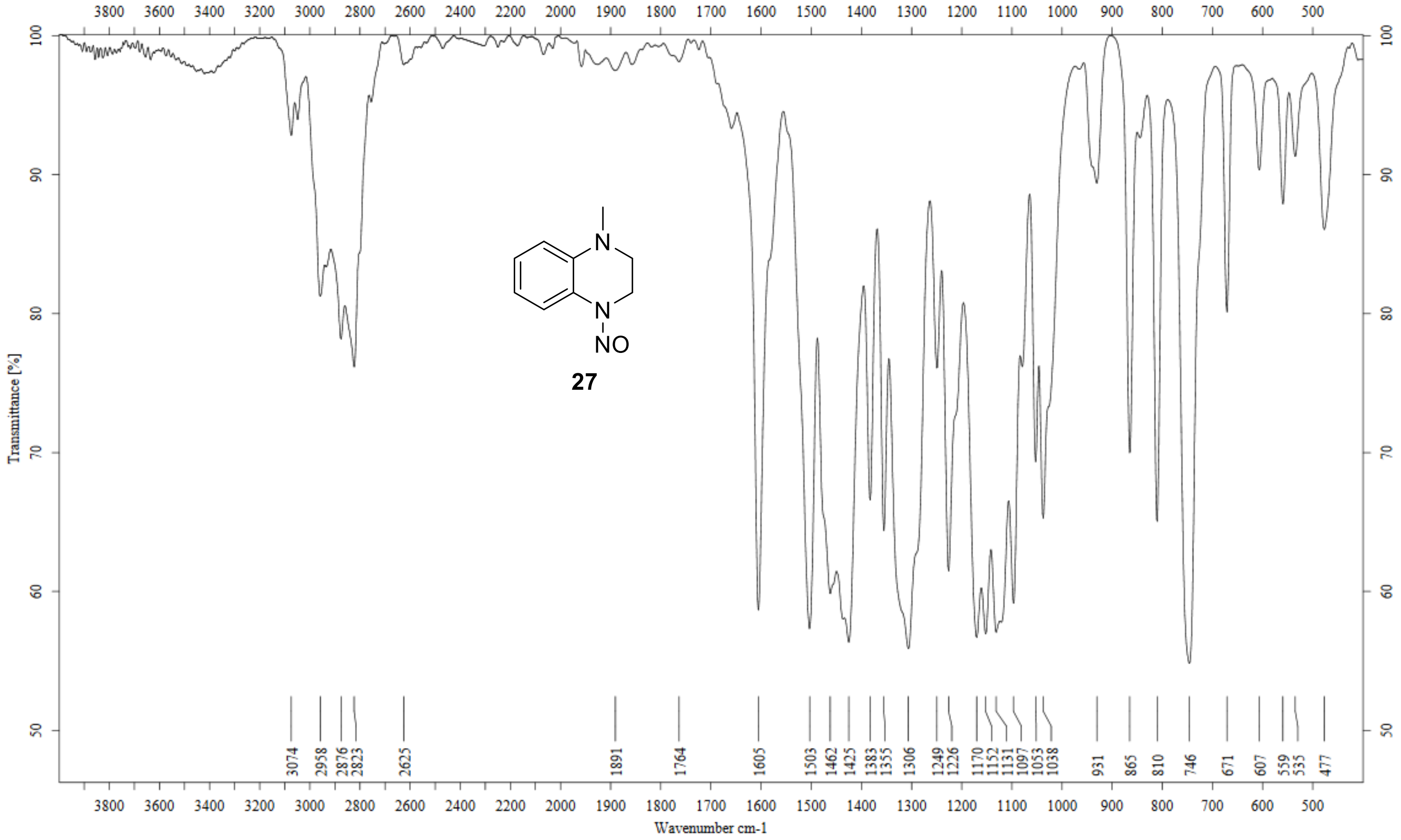

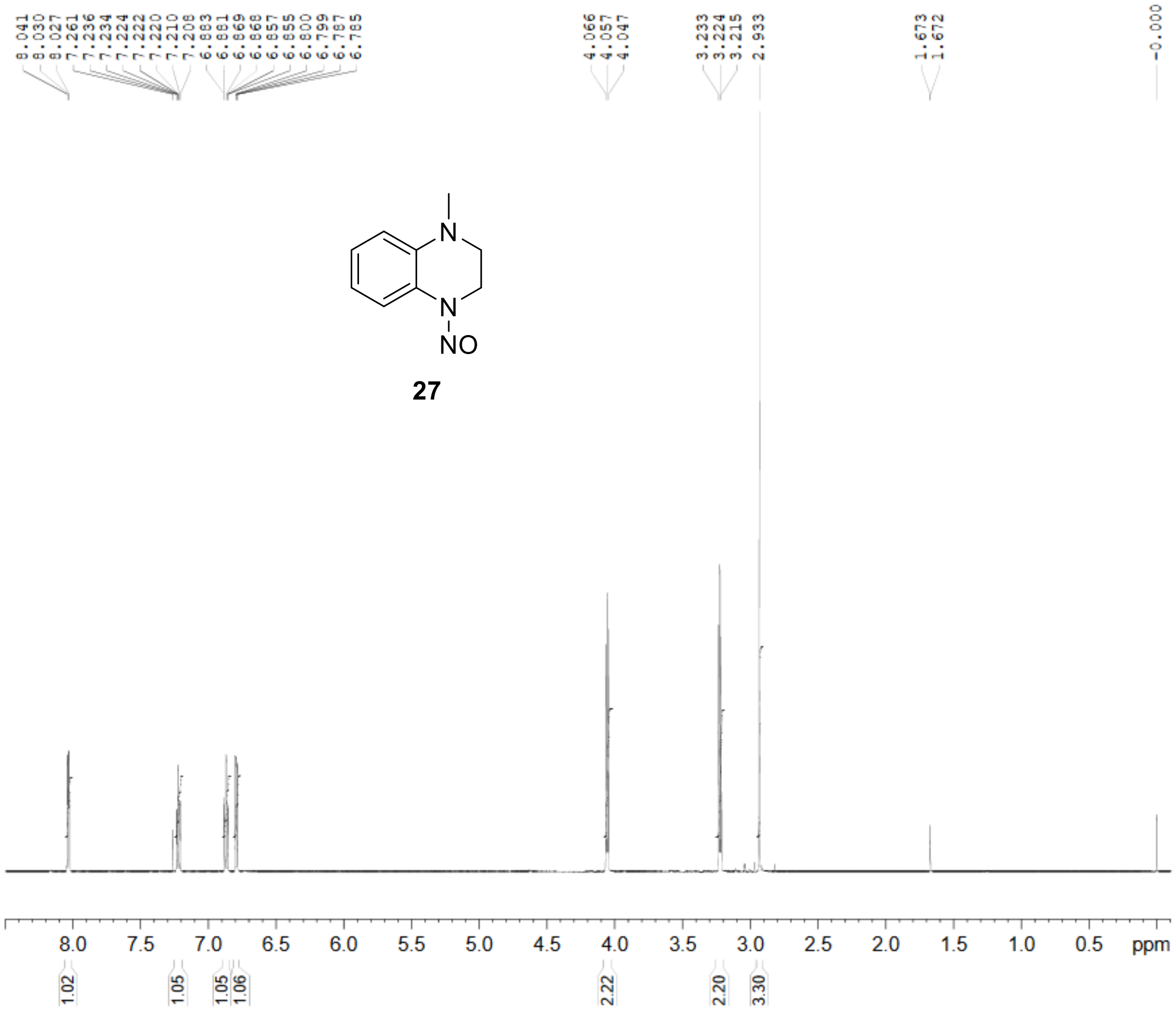

27

Standard 1H
135068
MIM0353_1
Milen Matyas
2020.10.28.(KP)

Current Data Parameters
NAME 135068
EXPNO 11
PROCNO 1

F2 - Acquisition Parameters
Date_ 20201028
Time_ 19.38 h
INSTRUM spect
PROBHD Z145856_0002 (
PULPROG zg30
TD 65536
SOLVENT CDCl3
NS 16
DS 2
SWH 12019.230 Hz
FIDRES 0.366798 Hz
AQ 2.7262976 sec
RG 89.22
DW 41.600 usec
DE 25.00 usec
TE 295.0 K
D1 1.00000000 sec
TD0 1
SFO1 600.1037056 MHz
NUC1 1H
P1 11.50 usec
PLW1 28.00000000 W

F2 - Processing parameters
SI 65536
SF 600.1000142 MHz
WDW no
SSB 0
LB 0 Hz
GB 0
PC 1.00

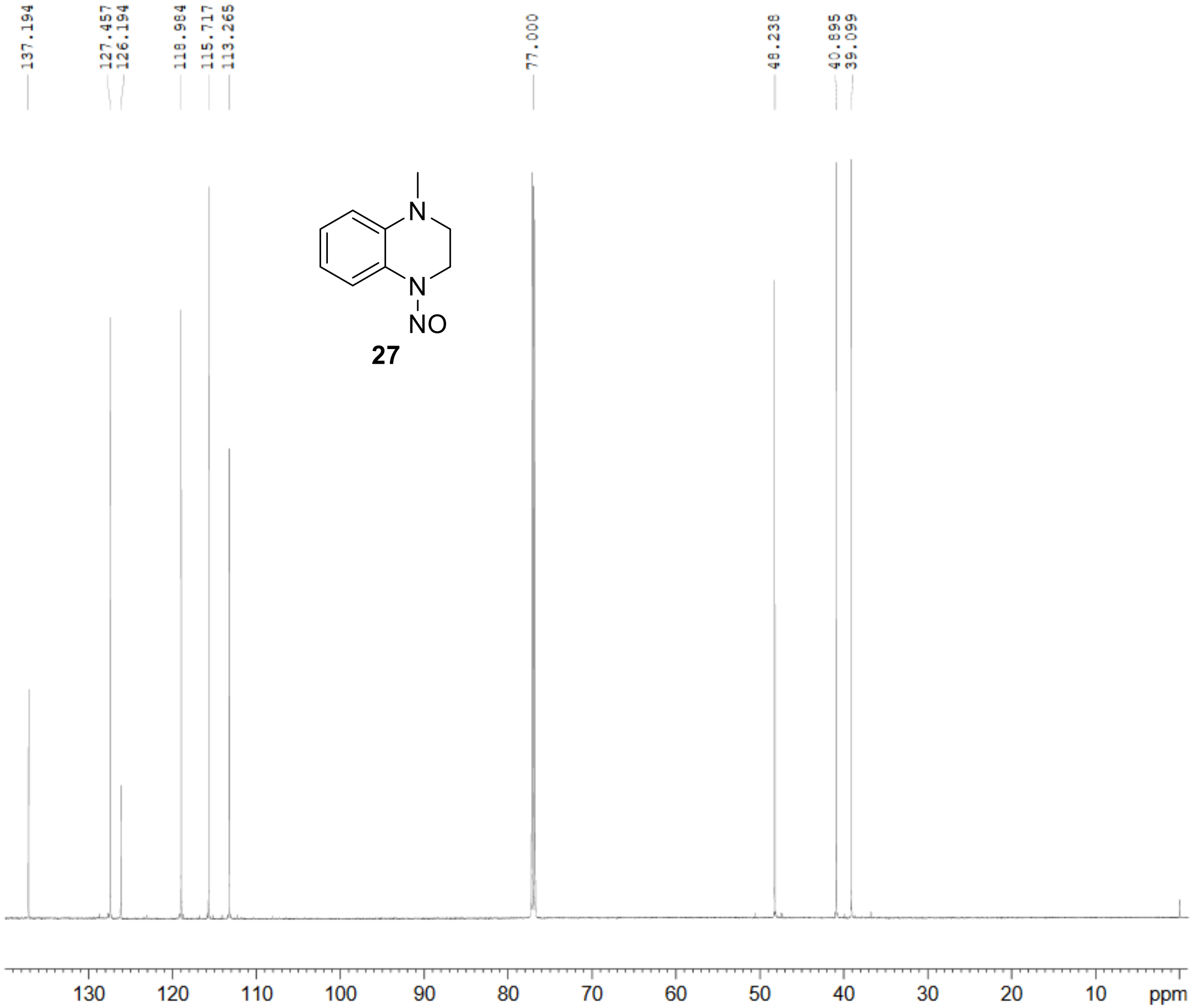

27

Standard 13C
135068
MIM0353_1
Milen Matyas
2020.10.28.(KP)

```
Current Data Parameters
NAME              135068
EXPNO                 12
PROCNO                 1

F2 - Acquisition Parameters
Date_           20201028
Time               20.48 h
INSTRUM            spect
PROBHD   Z145856_0002 (
PULPROG          zgpg30
TD                 65536
SOLVENT            CDCl3
NS                  2048
DS                     4
SWH            36231.883 Hz
FIDRES          1.105709 Hz
AQ             0.9043968 sec
RG                196.07
DW                13.800 usec
DE                 18.00 usec
TE                 295.0 K
D1            1.00000000 sec
D11           0.03000000 sec
TD0                    1
SFO1         150.9103545 MHz
NUC1                 13C
P1                 10.00 usec
PLW1         63.09999847 W
SFO2         600.1024004 MHz
NUC2                  1H
CPDPRG[2         waltz16
PCPD2              80.00 usec
PLW2         30.48800087 W
PLW12         0.63000000 W
PLW13         0.31637999 W

F2 - Processing parameters
SI                 32768
SF           150.8952779 MHz
WDW                   EM
SSB       0
LB                  1.00 Hz
GB        0
PC                  1.40
```

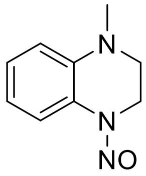

27

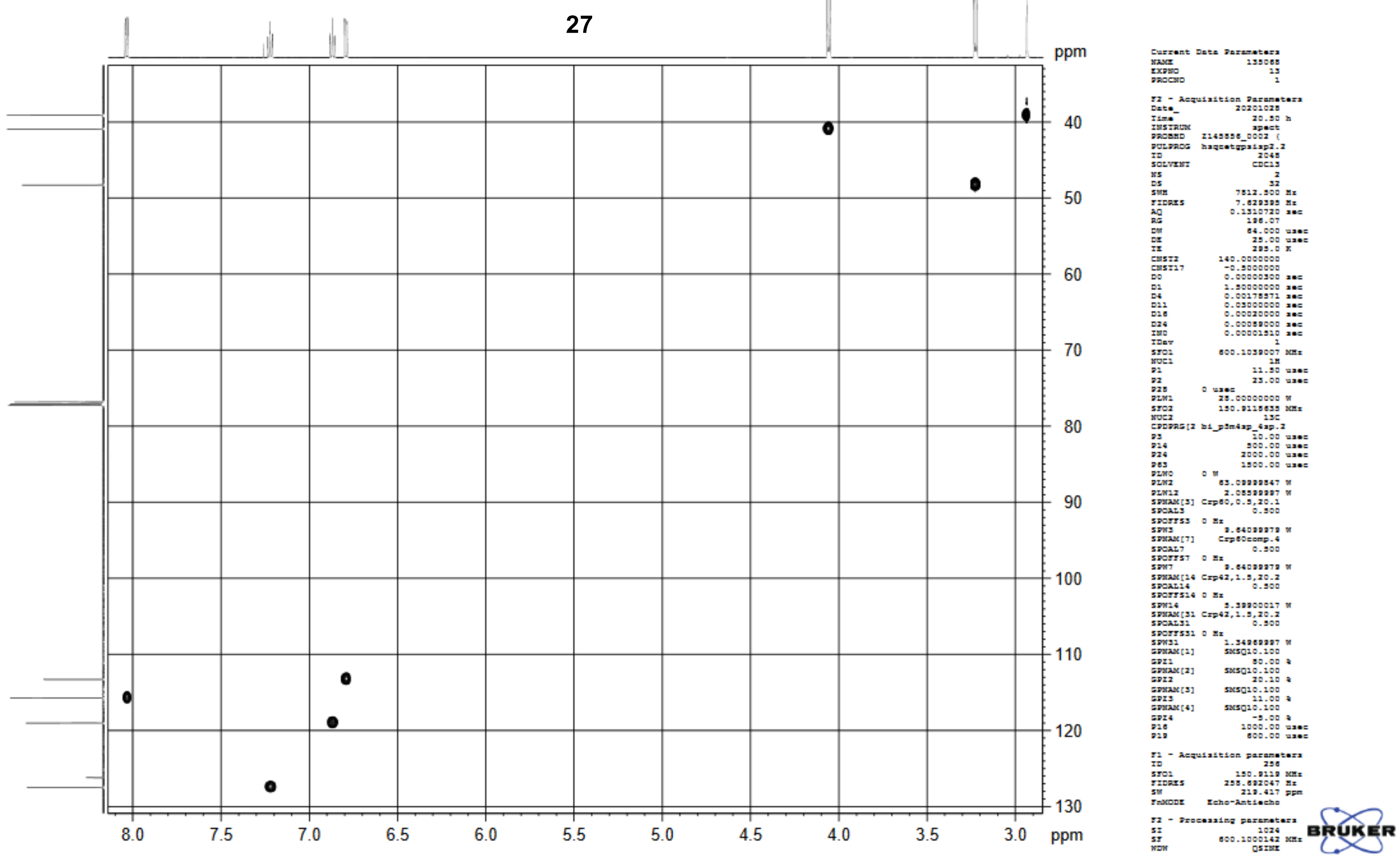
HSQC (140Hz)
135068
MIM0353_1
Milen Matyas
2020.10.28.(KP)
ppm
40
50
60
70
80
90
100
110
120
130
8.0
7.5
7.0
6.5
6.0
5.5
5.0
4.5
4.0
3.5
3.0
ppm
BRUKER

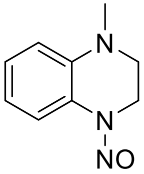
N
N
NO

27

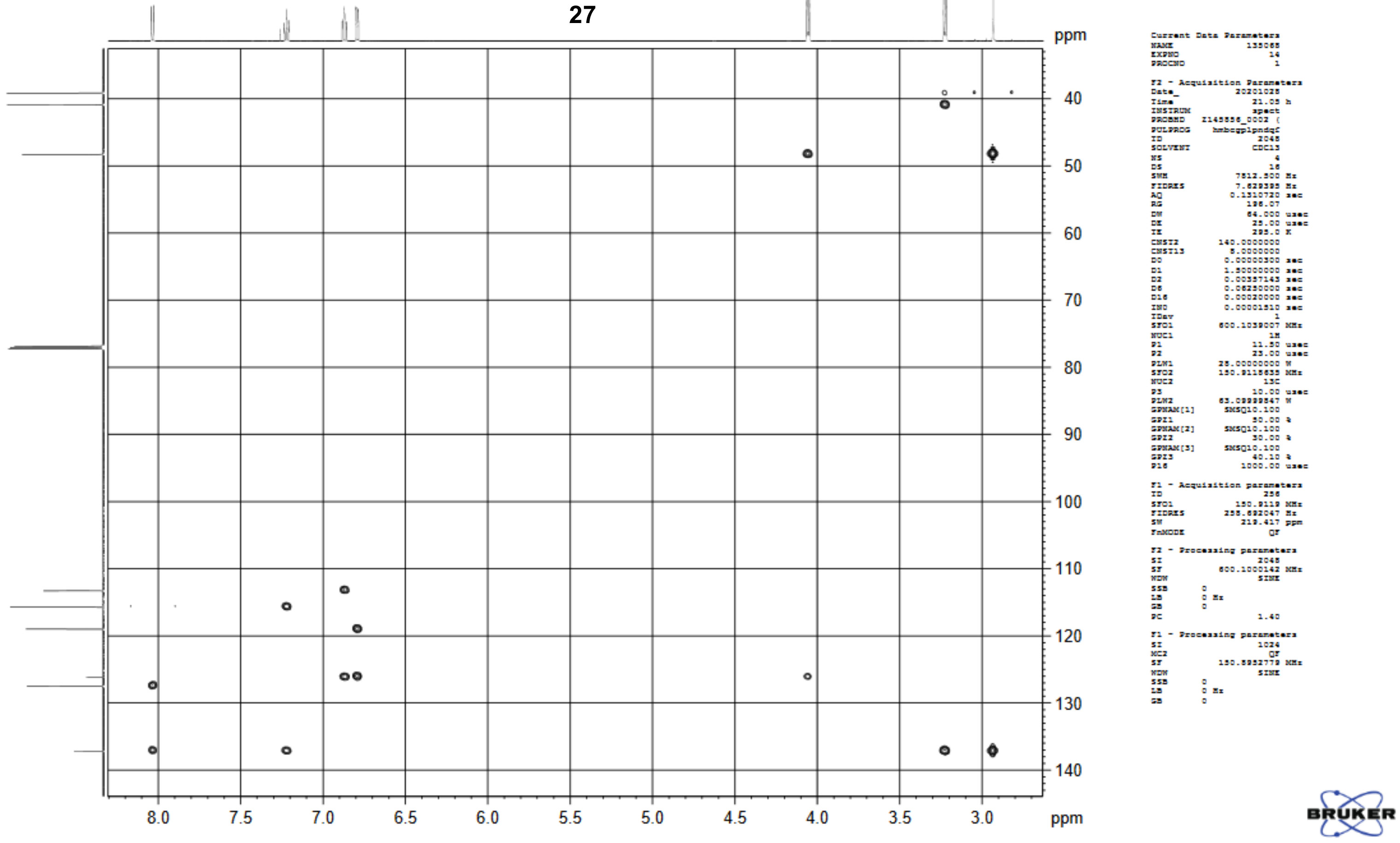
HMBC (8 Hz, 140Hz)
135068
MIM0353_1
Milen Matyas
2020.10.28.(KP)
ppm
40
50
60
70
80
90
100
110
120
130
140
8.0
7.5
7.0
6.5
6.0
5.5
5.0
4.5
4.0
3.5
3.0
ppm

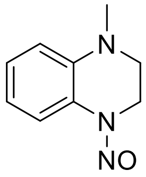
N
N
NO

27

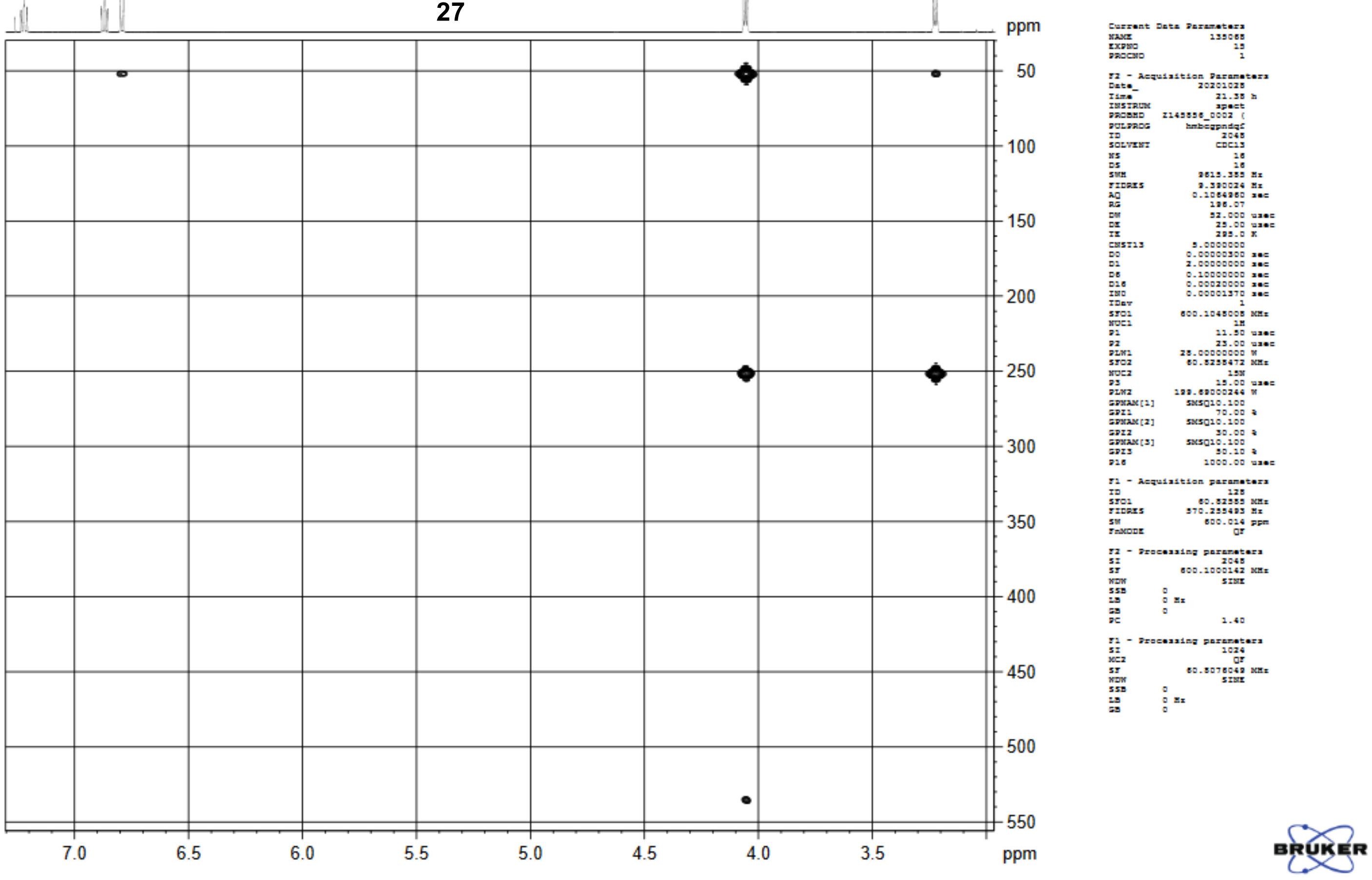
NHMBC (5 Hz)
135068
MIM0353_1
Milen Matyas
2020.10.28.(KP)
ppm
50
100
150
200
250
300
350
400
450
500
550
7.0
6.5
6.0
5.5
5.0
4.5
4.0
3.5
ppm
BRUKER

**125082**	Milen Matyas	KP	BRUKER Alpha
			Resolution: 2 cm-1
**JET0039_1**	KBr	2018.03.08.	Number of Scans: 16

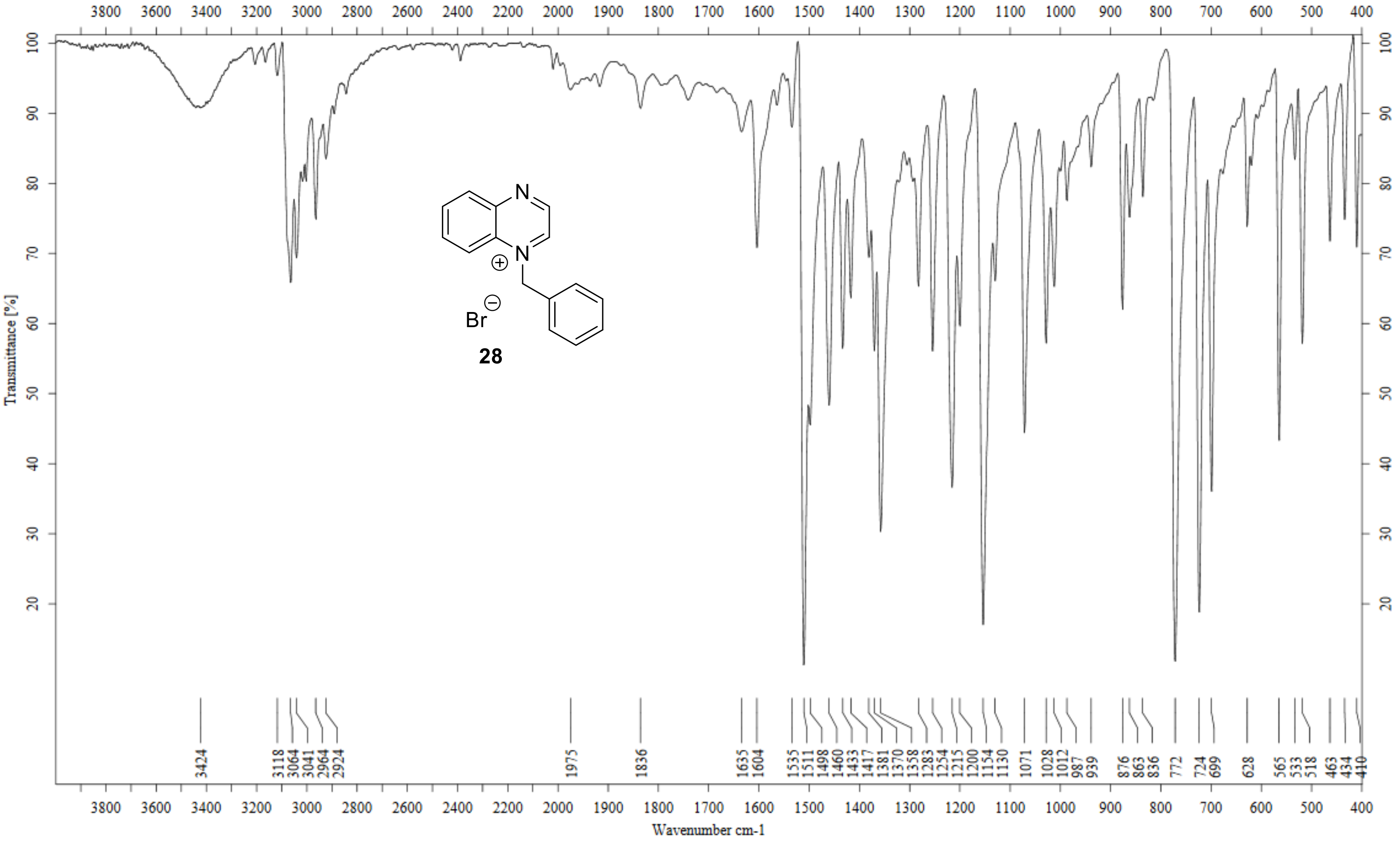

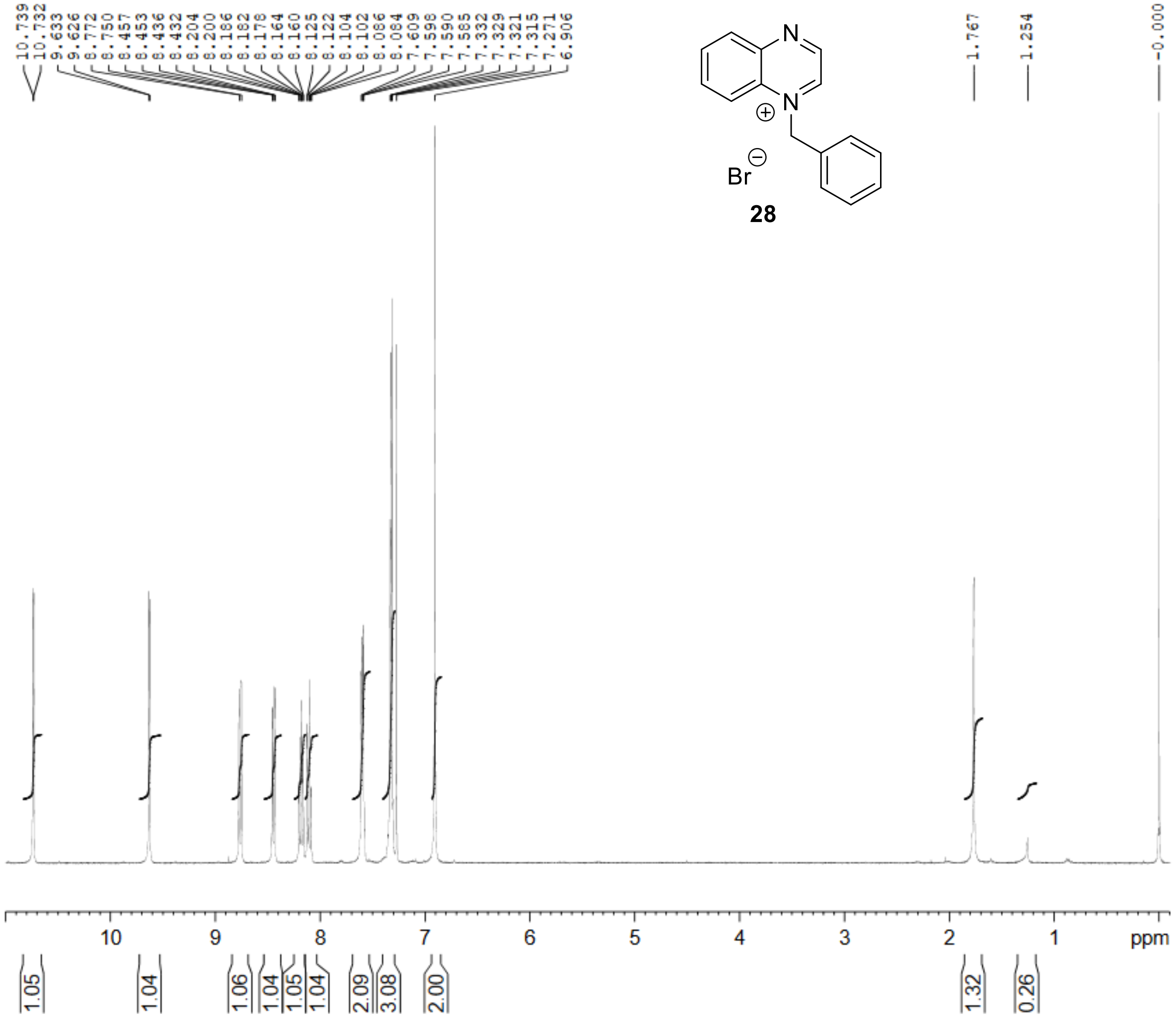


```
Standard 1H
125082
JET0039_1
Milen Matyas
2018.03.08. (DA)

Current Data Parameters
NAME              125082
EXPNO                  1
PROCNO                 1

F2 - Acquisition Parameters
Date_           20180308
Time               16.55 h
INSTRUM            spect
PROBHD   Z105190_0010 (
PULPROG             zg30
TD                 65536
SOLVENT            CDCl3
NS                    64
DS                     2
SWH             8223.685 Hz
FIDRES          0.250967 Hz
AQ             3.9845889 sec
RG                   287
DW                60.800 usec
DE                  6.50 usec
TE                 293.7 K
D1            1.00000000 sec
TD0                    1
SFO1         400.1324710 MHz
NUC1                  1H
P1                 12.70 usec
PLW1         11.39999962 W

F2 - Processing parameters
SI                 32768
SF           400.1300054 MHz
WDW                   no
SSB      0
LB       0 Hz
GB       0
PC                  1.00
```

BRUKER

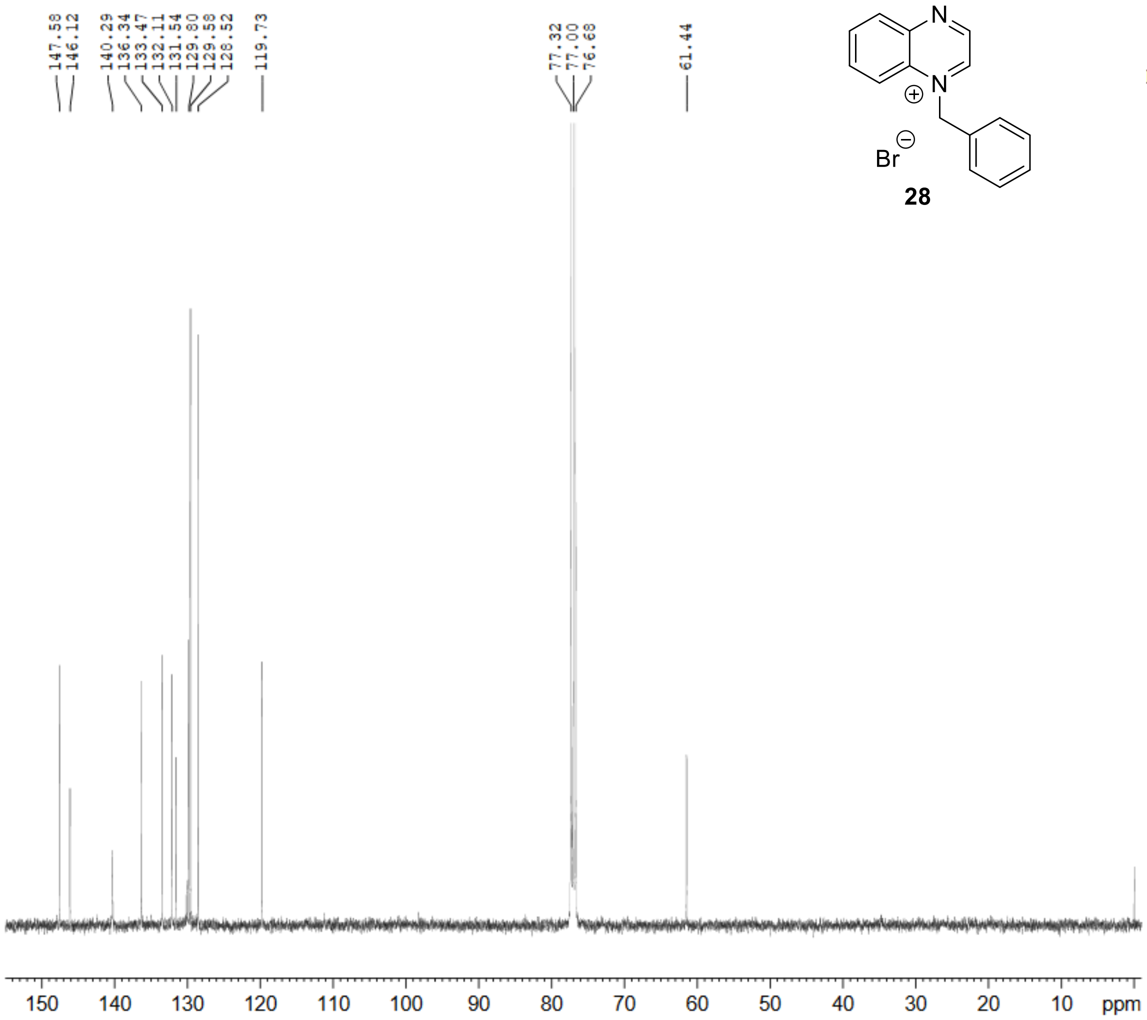

28

Standard 13C
125082
JET0039_1
Milen Matyas
2018.03.08. (DA)

```
Current Data Parameters
NAME              125082
EXPNO                  2
PROCNO                 1

F2 - Acquisition Parameters
Date_           20180308
Time               21.05 h
INSTRUM            spect
PROBHD   Z105190_0010 (
PULPROG          zgpg30
TD                 65536
SOLVENT            CDCl3
NS                  6144
DS                     4
SWH            24038.461 Hz
FIDRES          0.733596 Hz
AQ             1.3631488 sec
RG                  2050
DW                20.800 usec
DE                  6.50 usec
TE                 295.1 K
D1            1.00000000 sec
D11           0.03000000 sec
TD0                    1
SFO1         100.6228298 MHz
NUC1                 13C
P1                  8.80 usec
PLW1         46.20000076 W
SFO2         400.1316005 MHz
NUC2                  1H
CPDPRG[2         waltz16
PCPD2              90.00 usec
PLW2         11.39999962 W
PLW12         0.22700000 W
PLW13         0.11418000 W

F2 - Processing parameters
SI                 32768
SF           100.6127728 MHz
WDW                   EM
SSB      0
LB                  1.00 Hz
GB       0
PC                  1.40
```

**125191**	
**JET0041_1**	

Milen Matyas	KP
KBr	2018.03.14.

BRUKER Alpha
Resolution: 2 cm-1
Number of Scans: 16

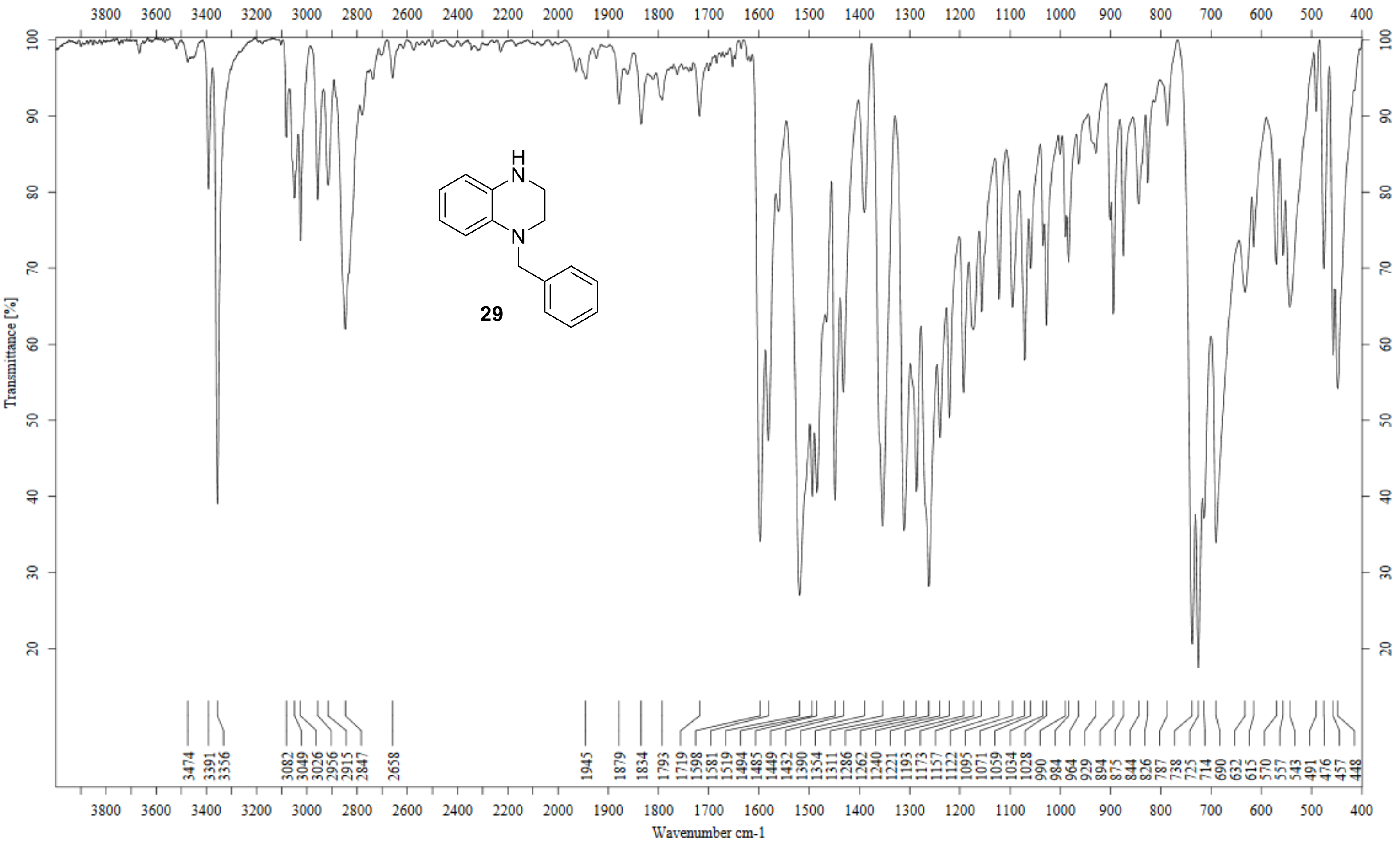

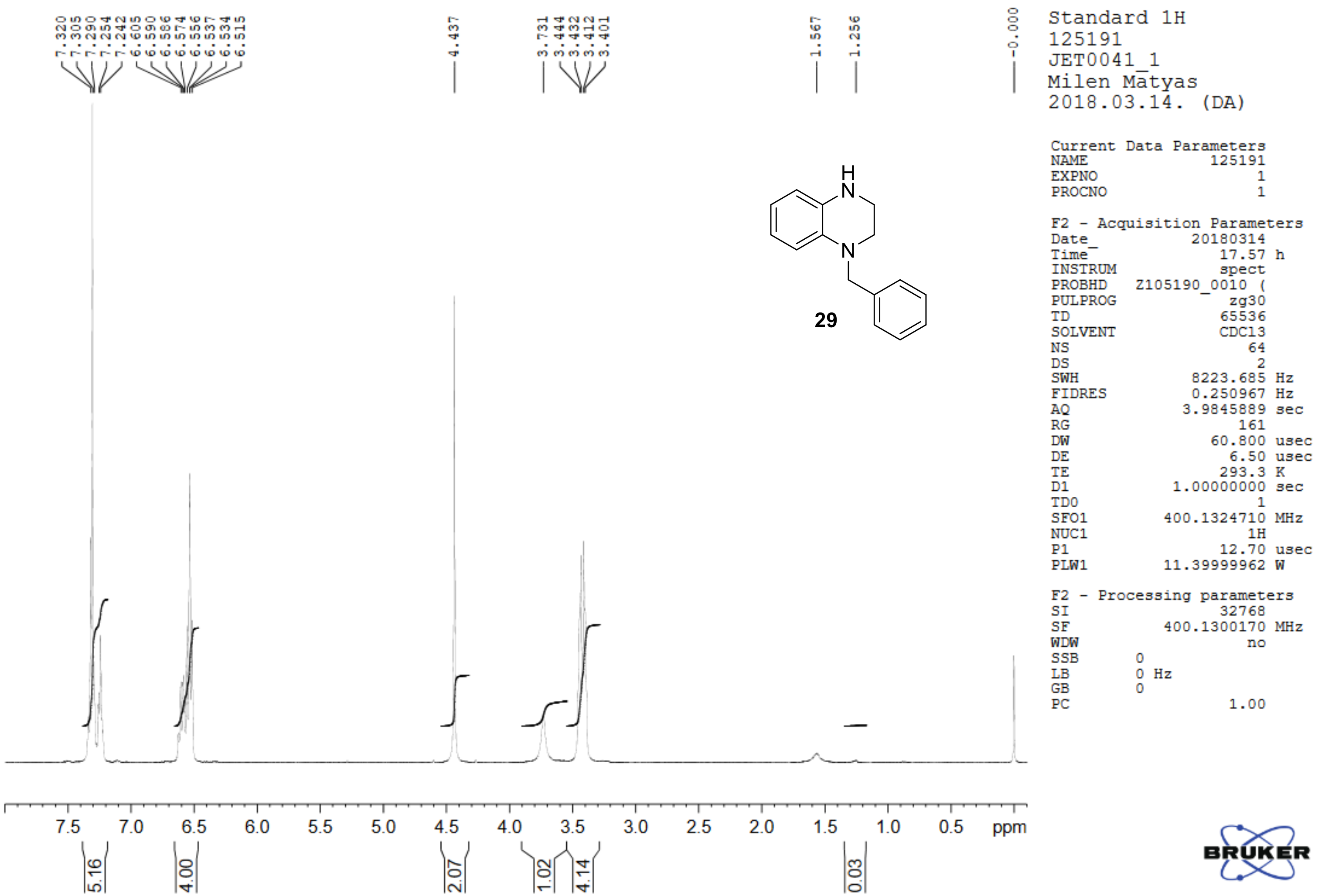
7.320
7.305
7.290
7.254
7.242
6.605
6.590
6.586
6.574
6.556
6.537
6.534
6.515
4.437
3.731
3.444
3.432
3.412
3.401
1.567
1.256
-0.000
29
7.5 7.0 6.5 6.0 5.5 5.0 4.5 4.0 3.5 3.0 2.5 2.0 1.5 1.0 0.5 ppm
5.16
4.00
2.07
1.02
4.14
0.03
Standard 1H
125191
JET0041_1
Milen Matyas
2018.03.14. (DA)
Current Data Parameters
NAME 125191
EXPNO 1
PROCNO 1
F2 - Acquisition Parameters
Date_ 20180314
Time 17.57 h
INSTRUM spect
PROBHD Z105190_0010 (
PULPROG zg30
TD 65536
SOLVENT CDCl3
NS 64
DS 2
SWH 8223.685 Hz
FIDRES 0.250967 Hz
AQ 3.9845889 sec
RG 161
DW 60.800 usec
DE 6.50 usec
TE 293.3 K
D1 1.00000000 sec
TD0 1
SFO1 400.1324710 MHz
NUC1 1H
P1 12.70 usec
PLW1 11.39999962 W
F2 - Processing parameters
SI 32768
SF 400.1300170 MHz
WDW no
SSB 0
LB 0 Hz
GB 0
PC 1.00

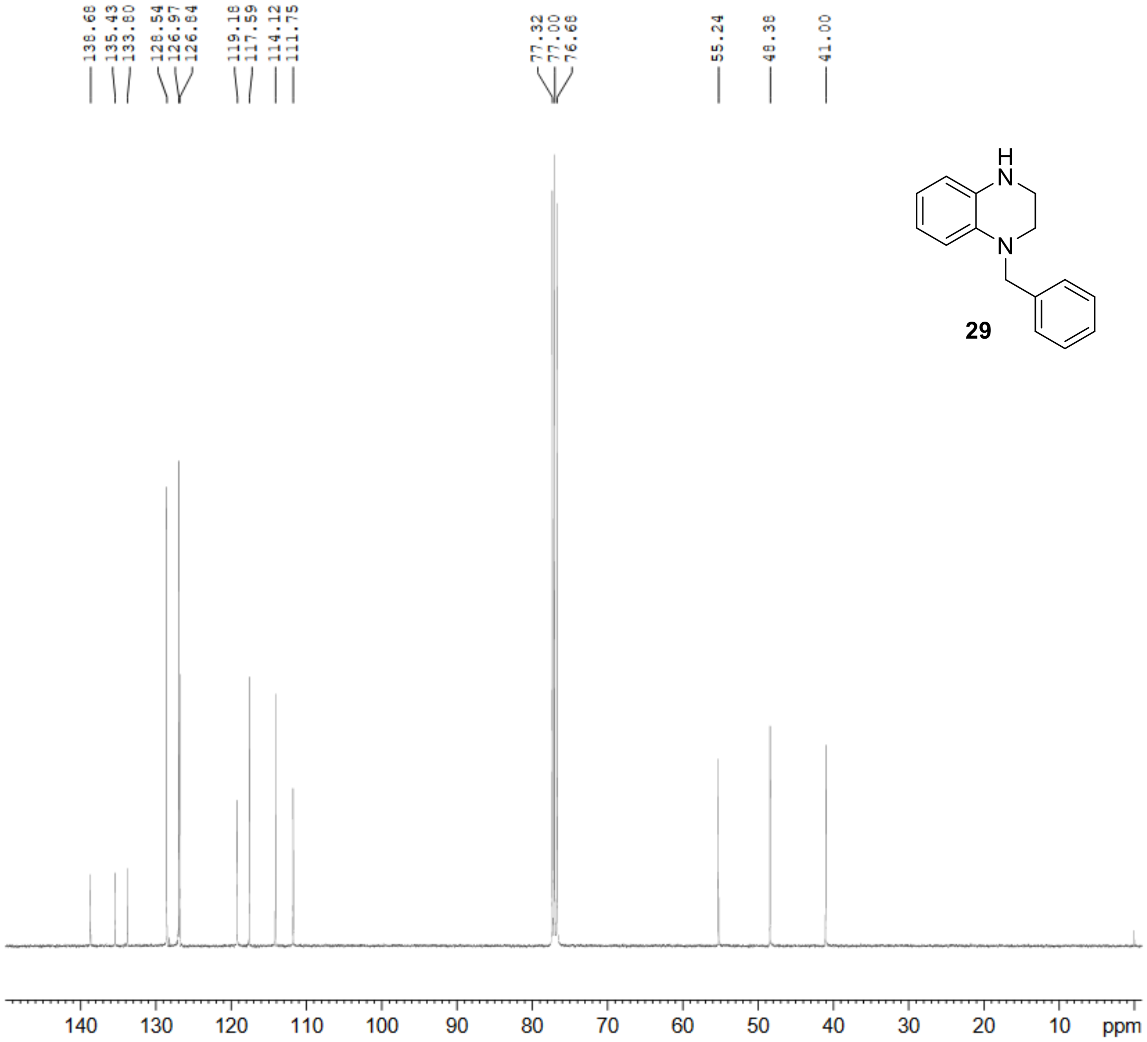

29

Standard 13C
125191
JET0041_1
Milen Mātyas
2018.03.14. (DA)

Current Data Parameters		
NAME	125191	
EXPNO	2	
PROCNO	1	
F2 - Acquisition Parameters		
Date_	20180314	
Time	23.30	h
INSTRUM	spect	
PROBHD	Z105190_0010 (	
PULPROG	zgpg30	
TD	65536	
SOLVENT	CDCl3	
NS	8192	
DS	4	
SWH	24038.461	Hz
FIDRES	0.733596	Hz
AQ	1.3631488	sec
RG	2050	
DW	20.800	usec
DE	6.50	usec
TE	294.1	K
D1	1.00000000	sec
D11	0.03000000	sec
TD0	1	
SFO1	100.6228298	MHz
NUC1	13C	
P1	8.80	usec
PLW1	46.20000076	W
SFO2	400.1316005	MHz
NUC2	1H	
CPDPRG[2	waltz16	
PCPD2	90.00	usec
PLW2	11.39999962	W
PLW12	0.22700000	W
PLW13	0.11418000	W
F2 - Processing parameters		
SI	32768	
SF	100.6127744	MHz
WDW	EM	
SSB	0	
LB	1.00	Hz
GB	0	
PC	1.40	

BRUKER

125121	Milen Matyas	KP	BRUKER Alpha
JET0040_1	KBr	2018.03.09.	Resolution: 2 cm-1
			Number of Scans: 16

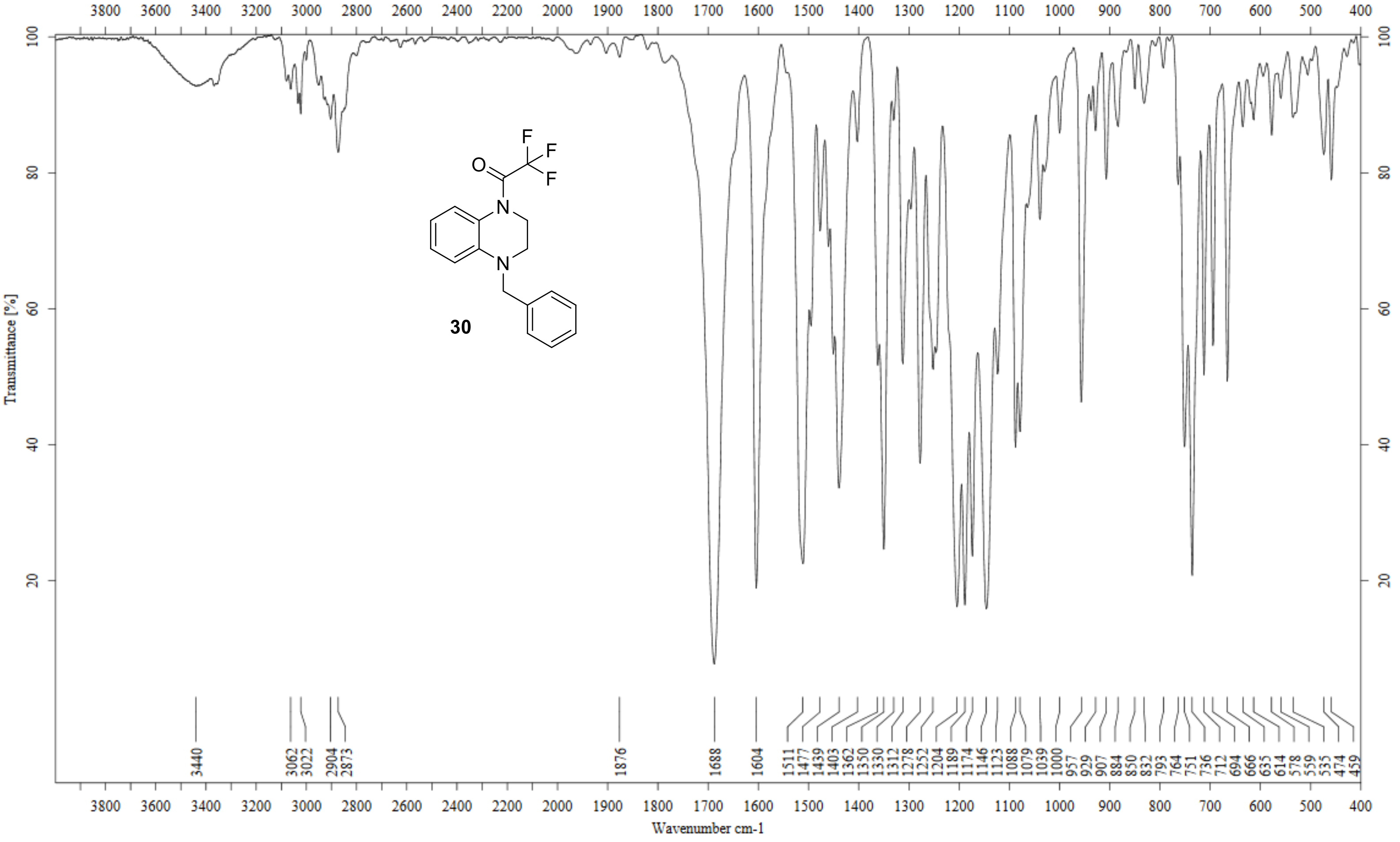

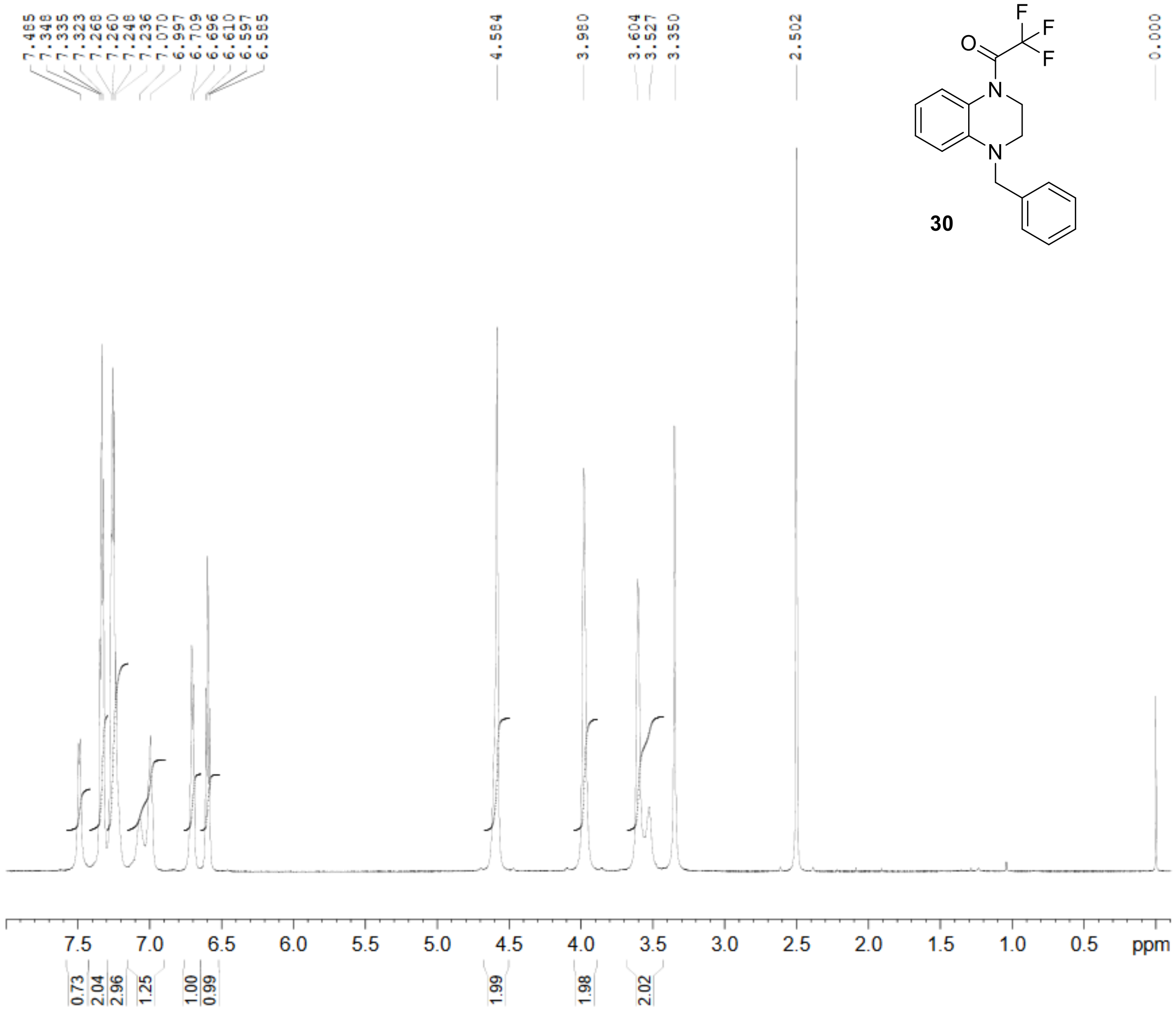


```
Standard 1H
125121
JET0040_1
Milen Matyas
2018.03.10. (HJ)

Current Data Parameters
NAME             125121
EXPNO                21
PROCNO                1

F2 - Acquisition Parameters
Date_          20180310
Time              16.40 h
INSTRUM           spect
PROBHD   Z145856_0002 (
PULPROG            zg30
TD                65536
SOLVENT            DMSO
NS                   16
DS                    4
SWH           12626.263 Hz
FIDRES         0.385323 Hz
AQ            2.5952256 sec
RG               133.88
DW               39.600 usec
DE                25.00 usec
TE                295.0 K
D1           1.00000000 sec
TD0                   1
SFO1        600.1642011 MHz
NUC1                 1H
P1                11.50 usec
PLW1        28.00000000 W

F2 - Processing parameters
SI                65536
SF          600.1600039 MHz
WDW                  no
SSB      0
LB       0 Hz
GB       0
PC                 1.00
```

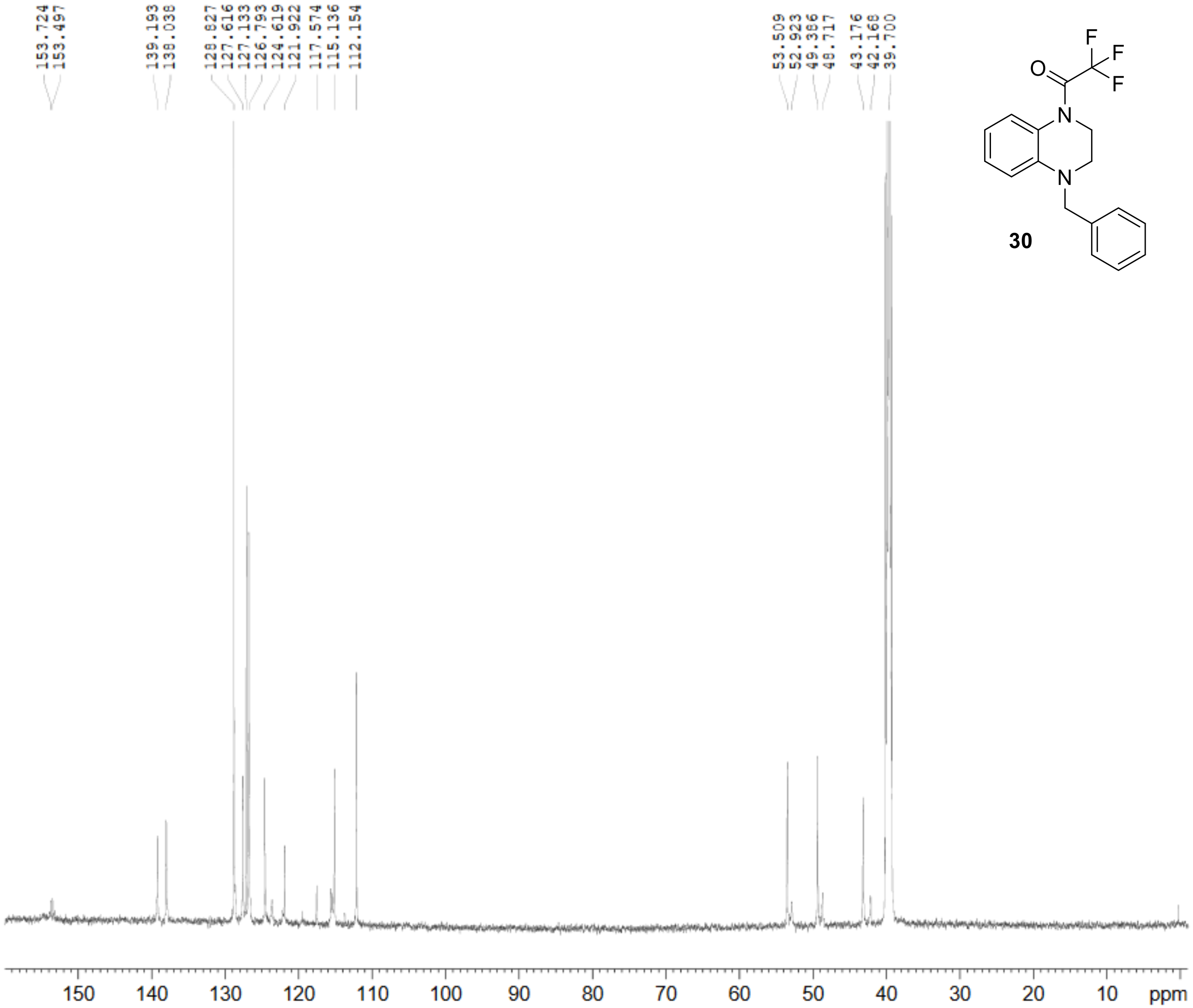

30

Standard 13C
125121
JET0040_1
Milen Matyas
2018.03.10. (HJ)

Current Data Parameters	
NAME	125121
EXPNO	22
PROCNO	1

F2 - Acquisition Parameters	
Date_	20180310
Time	19.30 h
INSTRUM	spect
PROBHD	Z145856_0002 (
PULPROG	zgpg30
TD	65536
SOLVENT	DMSO
NS	4096
DS	4
SWH	36057.691 Hz
FIDRES	1.100393 Hz
AQ	0.9087659 sec
RG	196.07
DW	13.867 usec
DE	18.00 usec
TE	295.0 K
D1	1.50000000 sec
D11	0.03000000 sec
TD0	1
SFO1	150.9254424 MHz
NUC1	13C
P1	10.00 usec
PLW1	60.36299896 W
SFO2	600.1624006 MHz
NUC2	1H
CPDPRG[2	waltz16
PCPD2	80.00 usec
PLW2	26.42600060 W
PLW12	0.59460002 W
PLW13	0.29861000 W

F2 - Processing parameters	
SI	65536
SF	150.9103920 MHz
WDW	EM
SSB	0
LB	3.00 Hz
GB	0
PC	1.40

**125128**	
**MIM0445_1**	

Milen Matyas	KP
KBr	2018.03.10.

BRUKER Alpha
Resolution: 2 cm-1
Number of Scans: 16

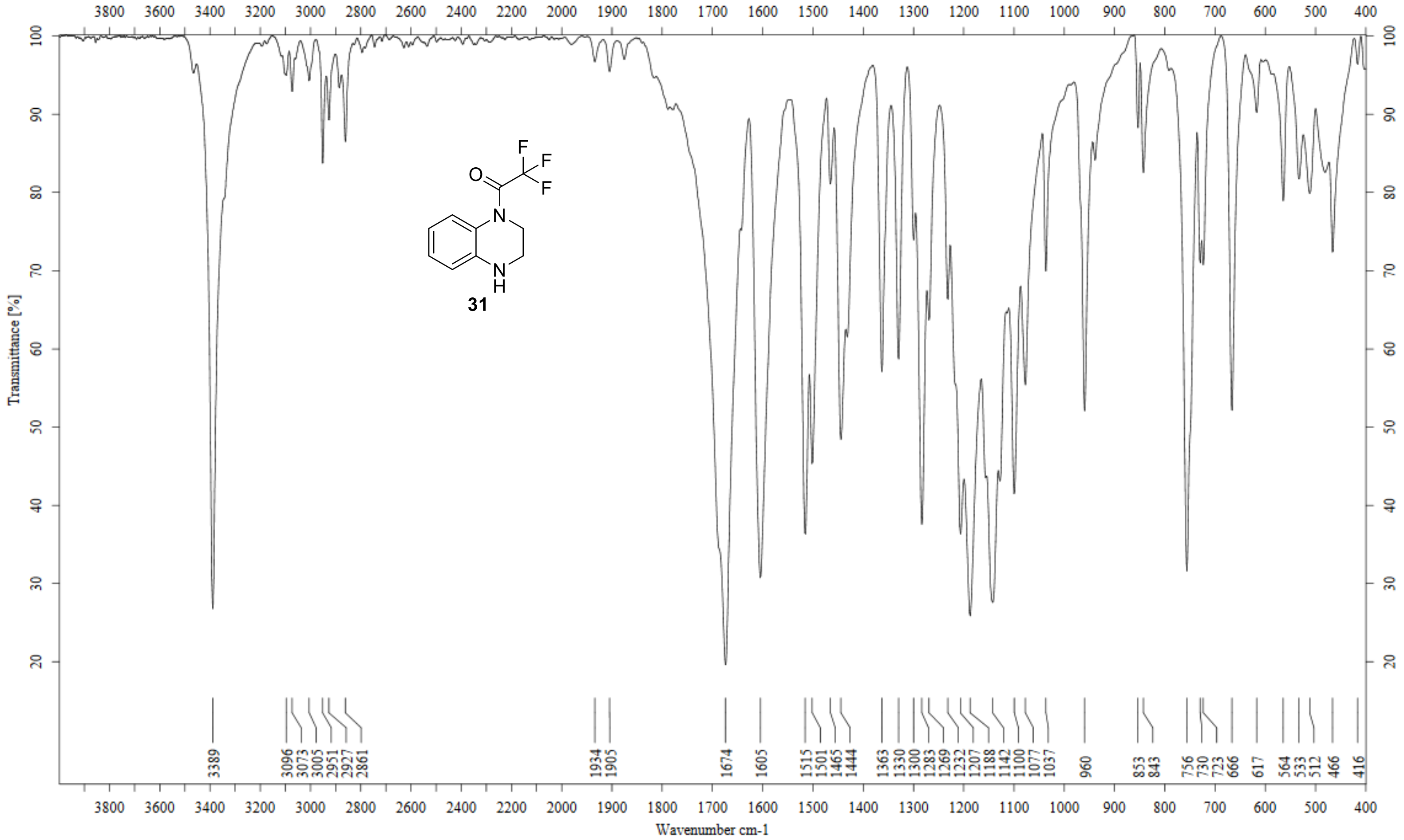

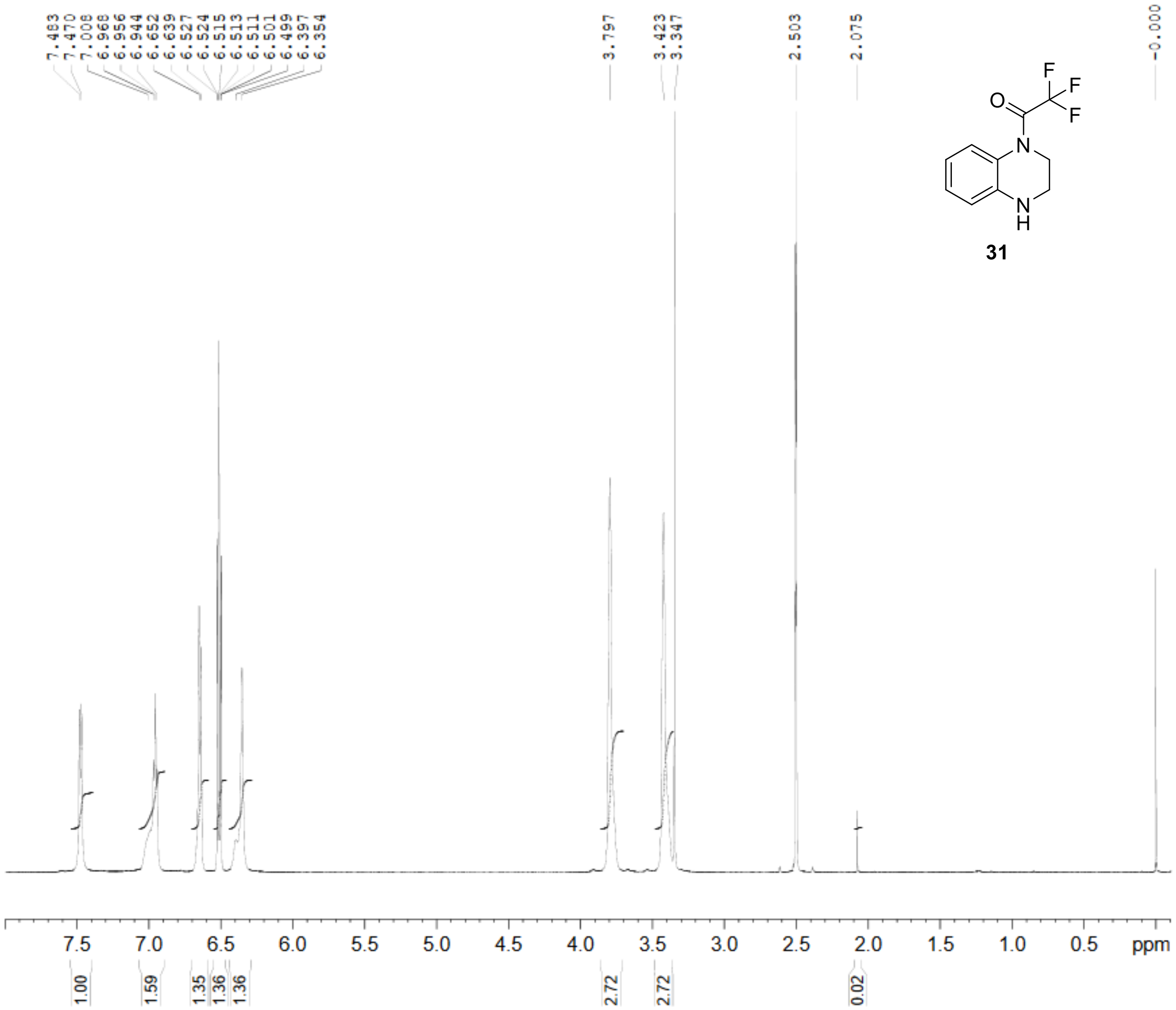

31

Standard 1H
125128
MIM0445_1
Milen Matyas
2018.03.10. (HJ)

Current Data Parameters

NAME	125128
EXPNO	11
PROCNO	1

F2 - Acquisition Parameters

Date_	20180310
Time_	19.36 h
INSTRUM	spect
PROBHD	Z145856_0002 (
PULPROG	zg30
TD	65536
SOLVENT	DMSO
NS	16
DS	4
SWH	12626.263 Hz
FIDRES	0.385323 Hz
AQ	2.5952256 sec
RG	174.19
DW	39.600 usec
DE	25.00 usec
TE	295.0 K
D1	1.00000000 sec
TD0	1
SFO1	600.1642011 MHz
NUC1	1H
P1	11.50 usec
PLW1	28.00000000 W

F2 - Processing parameters

SI	65536
SF	600.1600030 MHz
WDW	EM
SSB	0
LB	0.10 Hz
GB	0
PC	1.00

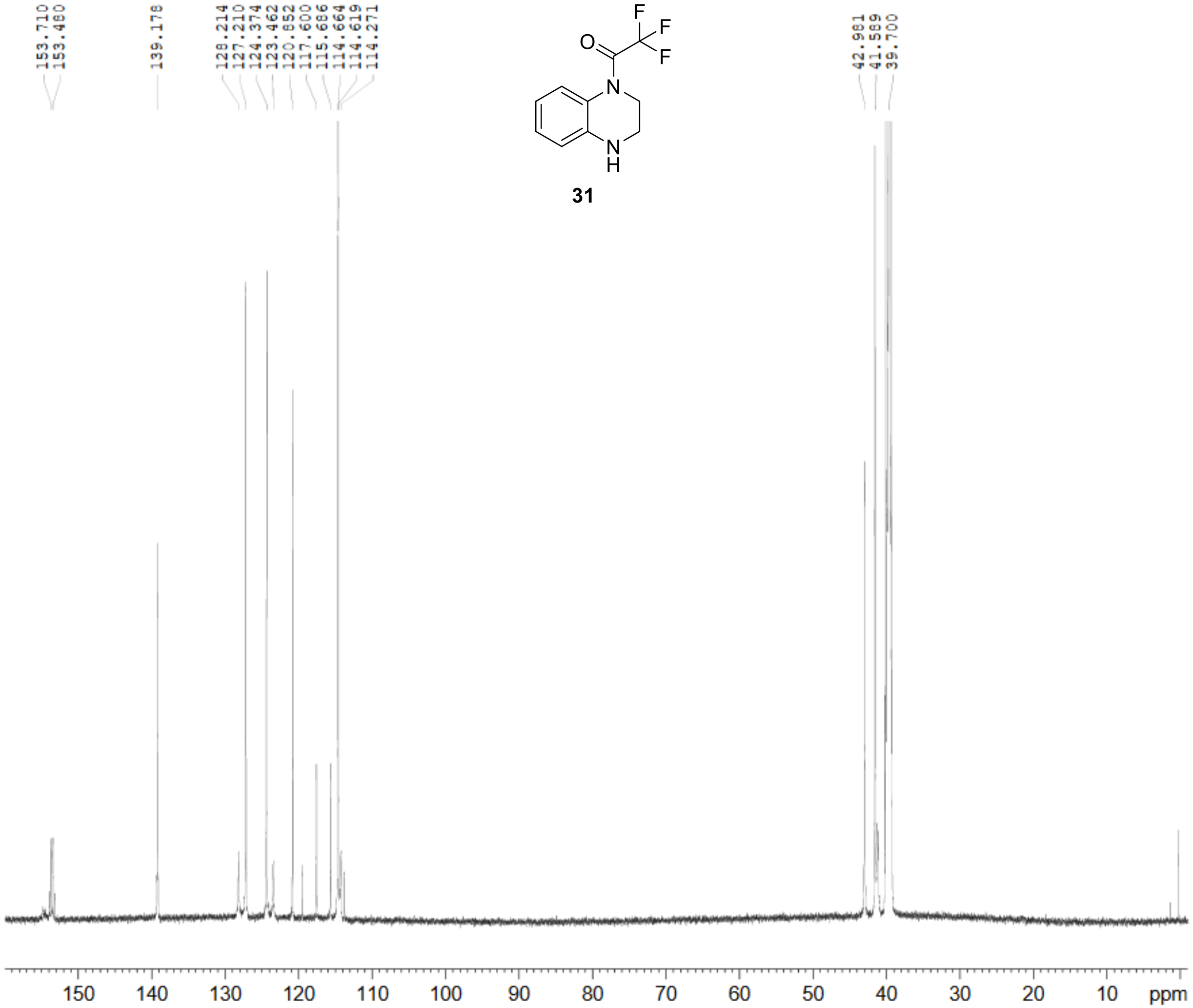

31

Standard 13C
125128
MIM0445_1
Milen Matyas
2018.03.10. (HJ)

Current Data Parameters
NAME 125128
EXPNO 12
PROCNO 1

F2 - Acquisition Parameters
Date_ 20180310
Time 22.26 h
INSTRUM spect
PROBHD Z145856_0002 (
PULPROG zgpg30
TD 65536
SOLVENT DMSO
NS 4096
DS 4
SWH 36057.691 Hz
FIDRES 1.100393 Hz
AQ 0.9087659 sec
RG 196.07
DW 13.867 usec
DE 18.00 usec
TE 295.0 K
D1 1.50000000 sec
D11 0.03000000 sec
TD0 1
SFO1 150.9254424 MHz
NUC1 13C
P1 10.00 usec
PLW1 60.36299896 W
SFO2 600.1624006 MHz
NUC2 1H
CPDPRG[2 waltz16
PCPD2 80.00 usec
PLW2 26.42600060 W
PLW12 0.59460002 W
PLW13 0.29861000 W

F2 - Processing parameters
SI 65536
SF 150.9103911 MHz
WDW EM
SSB 0
LB 1.00 Hz
GB 0
PC 1.40

**137810**	Milen Matyas	KP	BRUKER Alpha
**MIM0517_1**	KBr	2021.12.17.	Resolution: 2 cm-1
			Number of Scans: 16

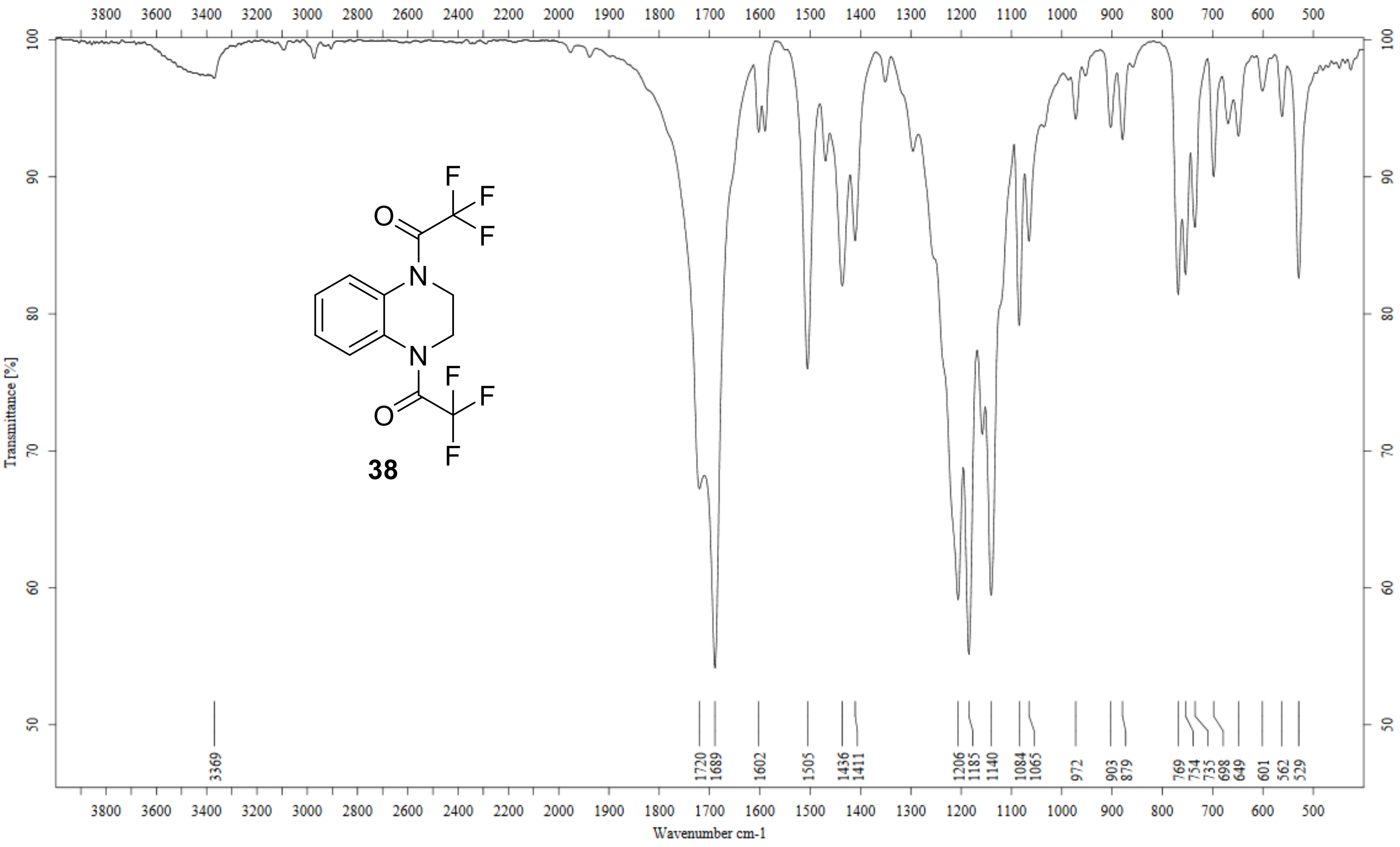

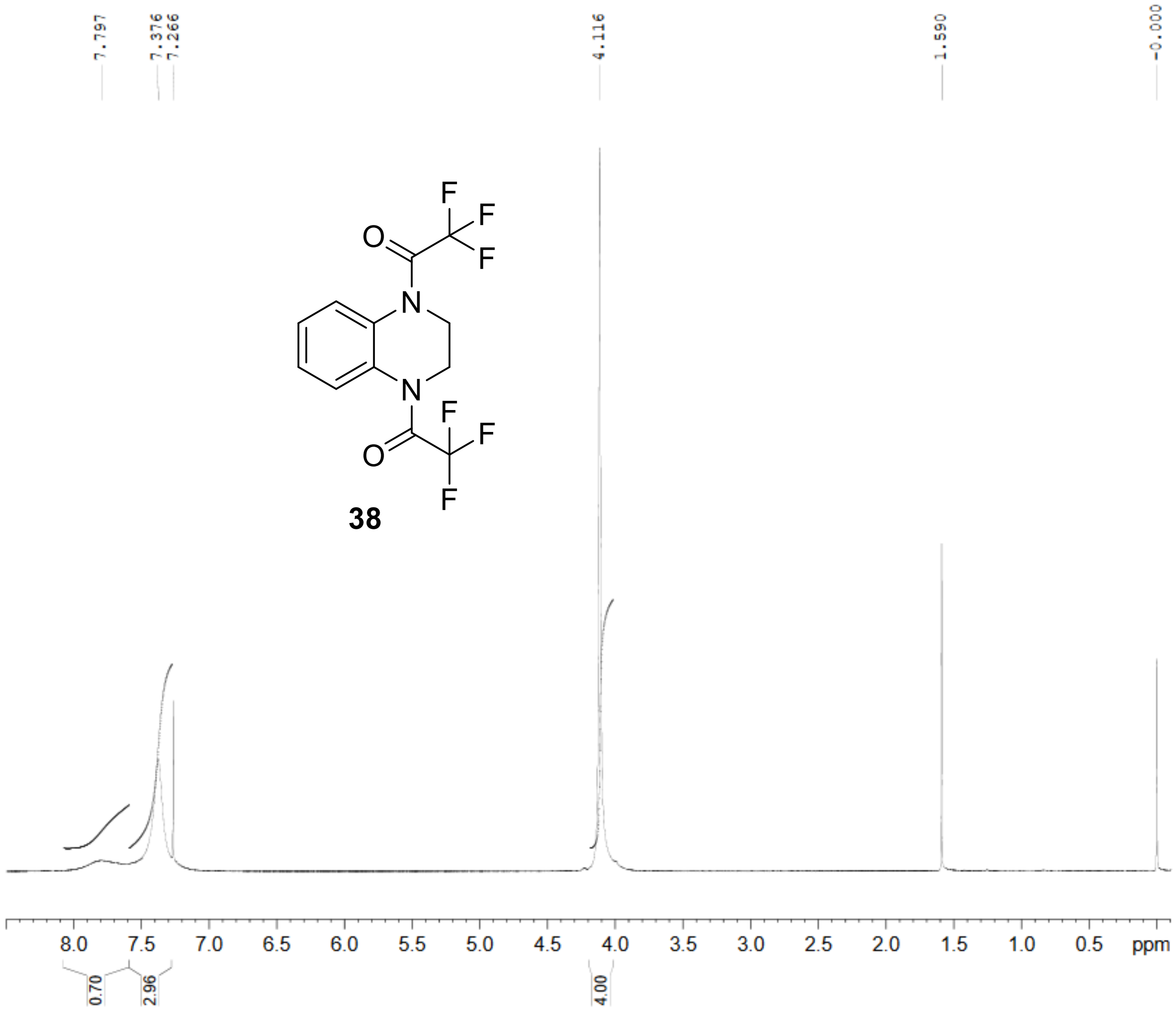

Standard 1H
137810
MIM0517_1
Milen Matyas
2021.12.17. (KP)

Current Data Parameters

NAME	137810
EXPNO	13
PROCNO	1

F2 - Acquisition Parameters

Date_	20211217
Time_	12.33 h
INSTRUM	spect
PROBHD	Z145856_0002 (
PULPROG	zg30
TD	65536
SOLVENT	CDCl3
NS	16
DS	2
SWH	12019.230 Hz
FIDRES	0.366798 Hz
AQ	2.7262976 sec
RG	196.07
DW	41.600 usec
DE	25.00 usec
TE	295.0 K
D1	1.00000000 sec
TD0	1
SFO1	600.0537053 MHz
NUC1	1H
P1	11.50 usec
PLW1	28.00000000 W

F2 - Processing parameters

SI	65536
SF	600.0500117 MHz
WDW	no
SSB	0
LB	0 Hz
GB	0
PC	1.00

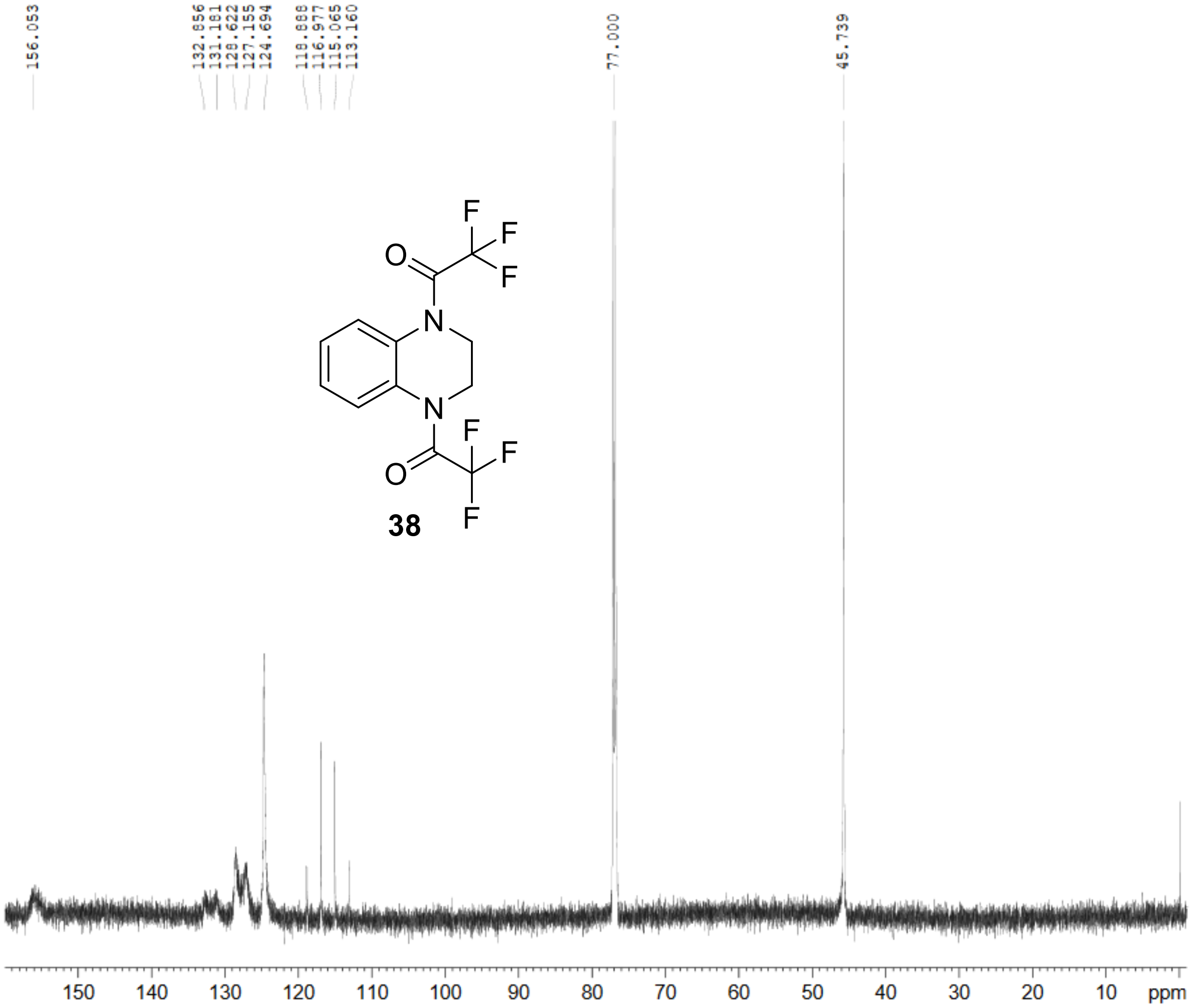

Standard 13C
137810
MIM0517_1
Milen Matyas
2021.12.16. (KP)

```
Current Data Parameters
NAME               137810
EXPNO                  12
PROCNO                  1

F2 - Acquisition Parameters
Date_            20211216
Time                17.28 h
INSTRUM             spect
PROBHD   Z145856_0002 (
PULPROG           zgpg30
TD                  65536
SOLVENT             CDCl3
NS                   2048
DS                      4
SWH             36231.883 Hz
FIDRES           1.105709 Hz
AQ              0.9043968 sec
RG                 196.07
DW                 13.800 usec
DE                  18.00 usec
TE                  295.0 K
D1             1.00000000 sec
D11            0.03000000 sec
TD0                     1
SFO1          150.8977808 MHz
NUC1                  13C
P1                  10.00 usec
PLW1          64.59899902 W
SFO2          600.0524002 MHz
NUC2                   1H
CPDPRG[2          waltz16
PCPD2               80.00 usec
PLW2          31.00000000 W
PLW12          0.69749999 W
PLW13          0.35029000 W

F2 - Processing parameters
SI                  32768
SF            150.8826990 MHz
WDW                    no
SSB      0
LB       0 Hz
GB       0
PC                   1.40
```

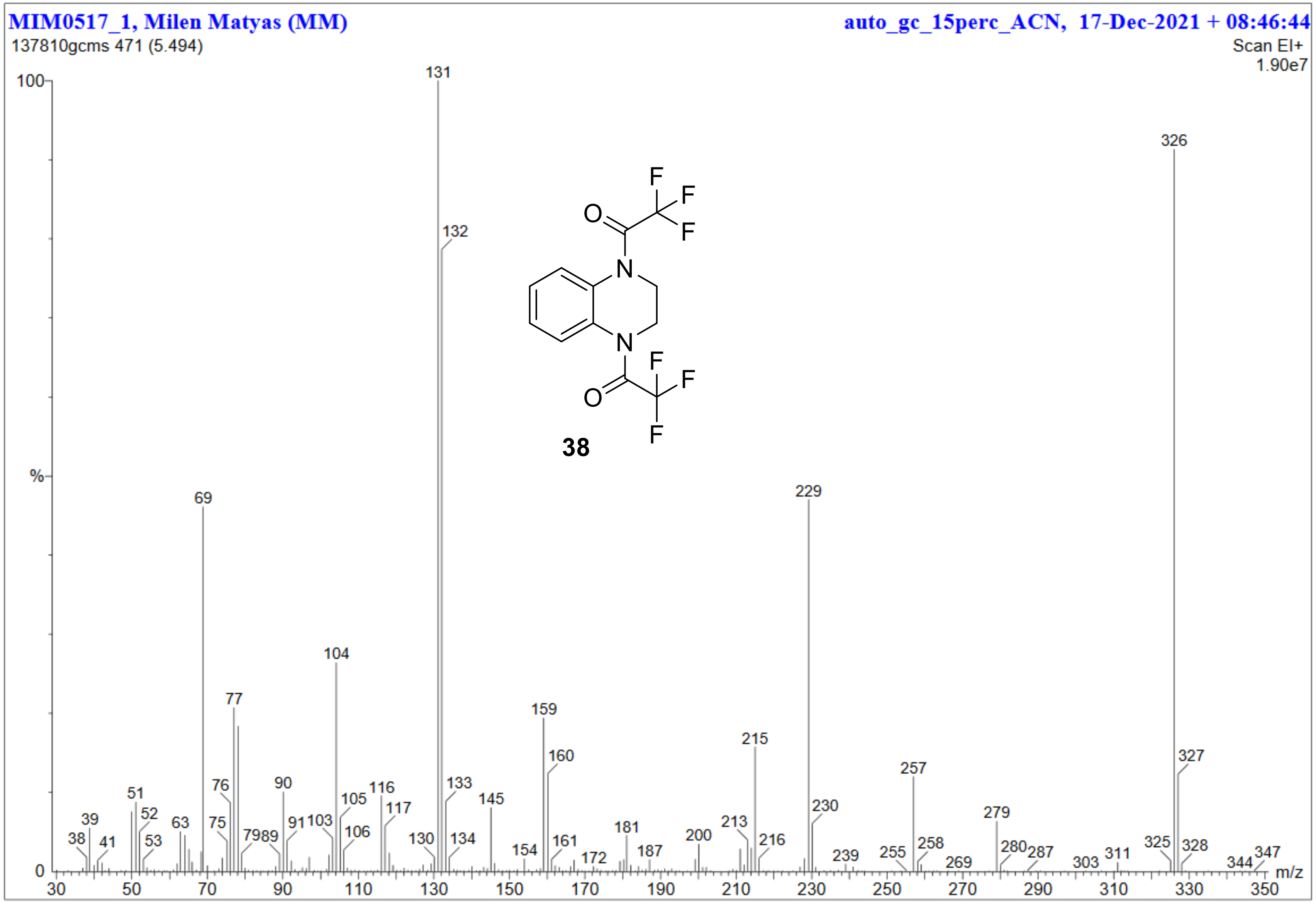
MIM0517_1, Milen Matyas (MM)
auto_gc_15perc_ACN, 17-Dec-2021 + 08:46:44
137810gcms 471 (5.494)
Scan EI+
1.90e7
100
%
0
m/z
30 50 70 90 110 130 150 170 190 210 230 250 270 290 310 330 350
131
326
132
69
229
104
77
159
215
160
327
257
133
145
230
279
38
39
41
51
52
53
63
75
76
79
89
90
91
103
105
106
116
117
130
134
154
161
172
181
187
200
213
216
239
255
258
269
280
287
303
311
325
328
344
347
F
F
F
O
N
N
O
F
F
F
38

**125192**	Milen Matyas	KP	BRUKER Alpha
**JET0042_1**	KBr	2018.03.14.	Resolution: 2 cm-1
			Number of Scans: 16

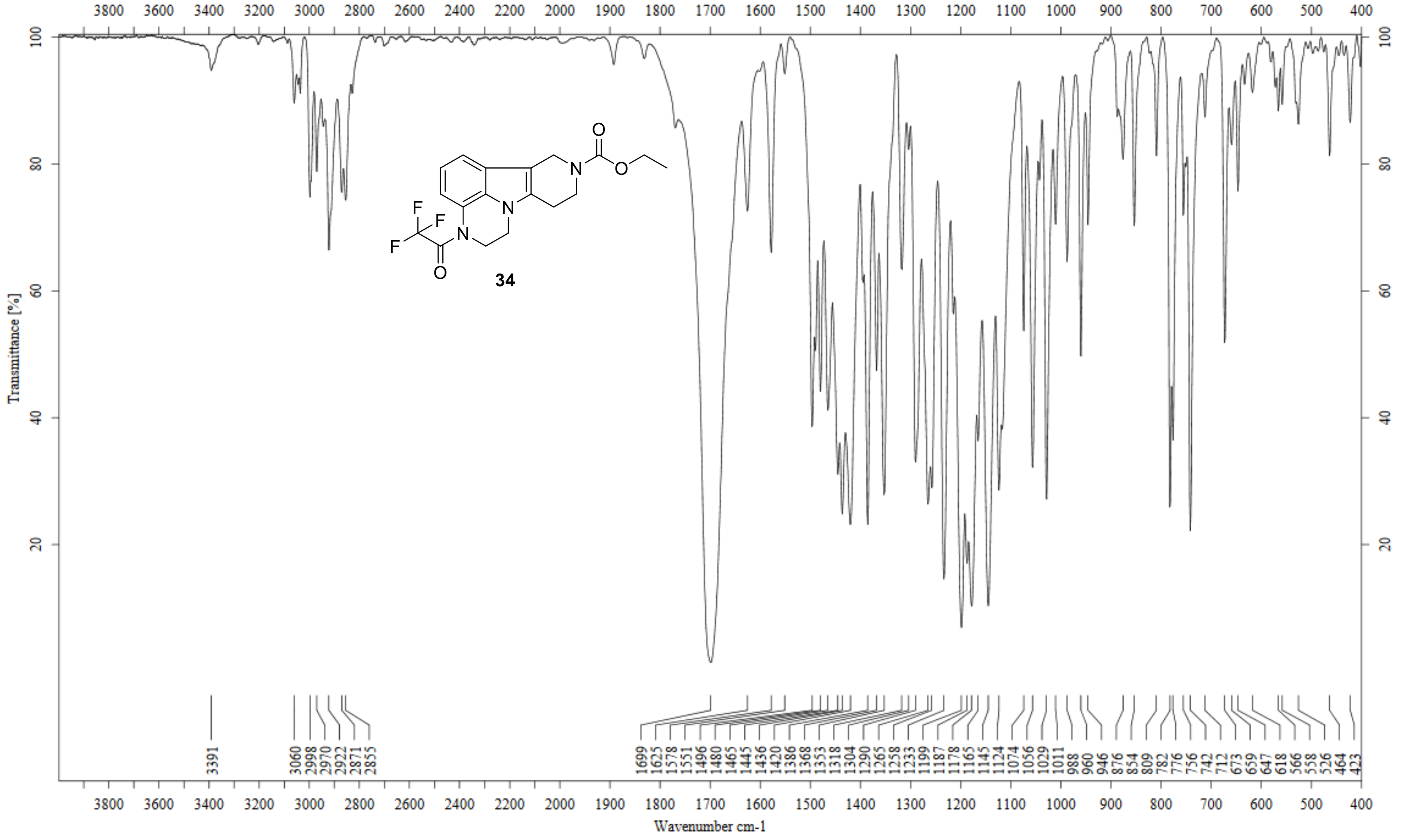

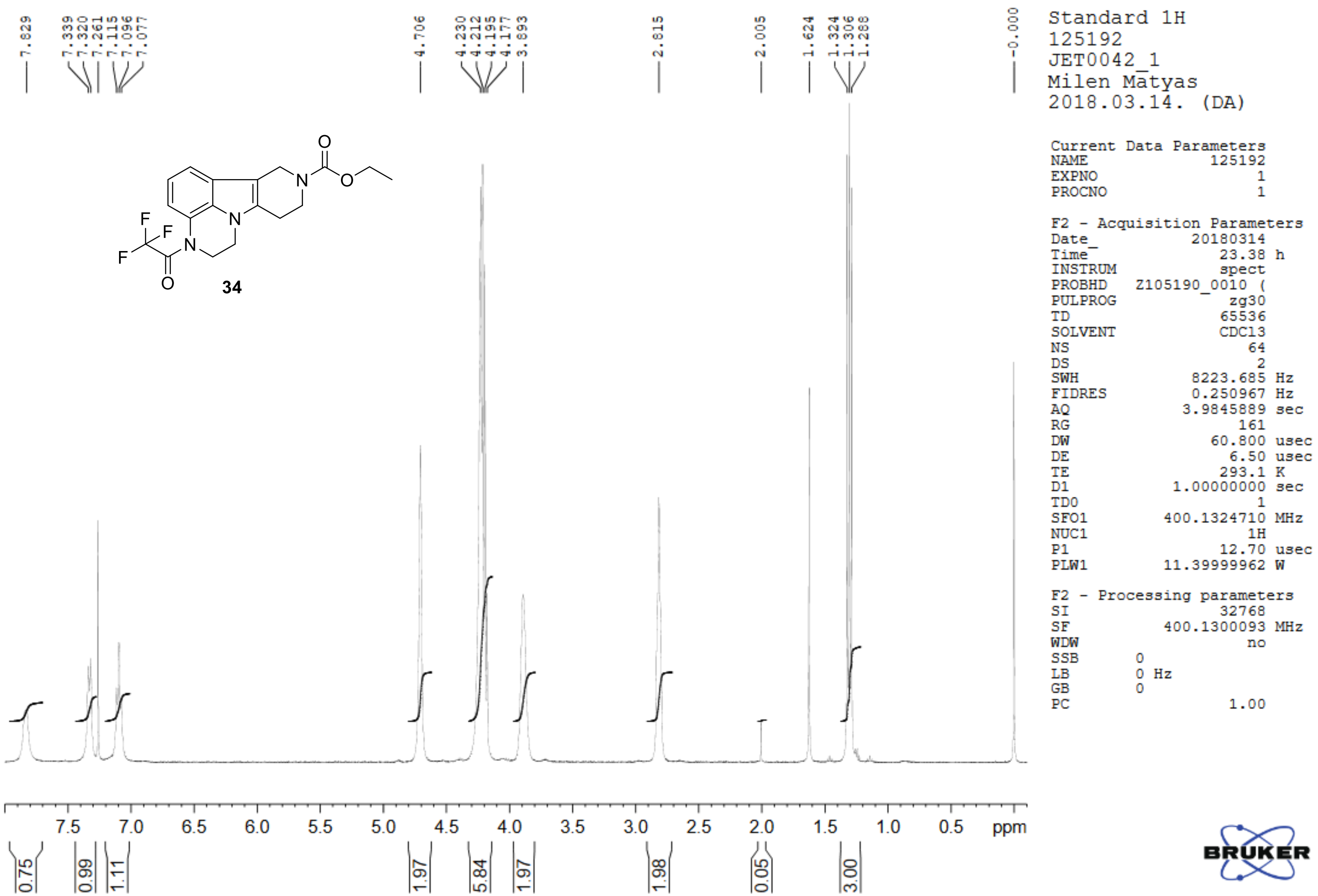

7.829
7.339
7.320
7.261
7.115
7.096
7.077
4.706
4.230
4.212
4.195
4.177
3.893
2.815
2.005
1.624
1.324
1.306
1.288
-0.000
Standard 1H
125192
JET0042_1
Milen Matyas
2018.03.14. (DA)
Current Data Parameters
NAME 125192
EXPNO 1
PROCNO 1
F2 - Acquisition Parameters
Date_ 20180314
Time 23.38 h
INSTRUM spect
PROBHD Z105190_0010 (
PULPROG zg30
TD 65536
SOLVENT CDCl3
NS 64
DS 2
SWH 8223.685 Hz
FIDRES 0.250967 Hz
AQ 3.9845889 sec
RG 161
DW 60.800 usec
DE 6.50 usec
TE 293.1 K
D1 1.00000000 sec
TD0 1
SFO1 400.1324710 MHz
NUC1 1H
P1 12.70 usec
PLW1 11.39999962 W
F2 - Processing parameters
SI 32768
SF 400.1300093 MHz
WDW no
SSB 0
LB 0 Hz
GB 0
PC 1.00
34
7.5 7.0 6.5 6.0 5.5 5.0 4.5 4.0 3.5 3.0 2.5 2.0 1.5 1.0 0.5 ppm
0.75 0.99 1.11 1.97 5.84 1.97 1.98 0.05 3.00

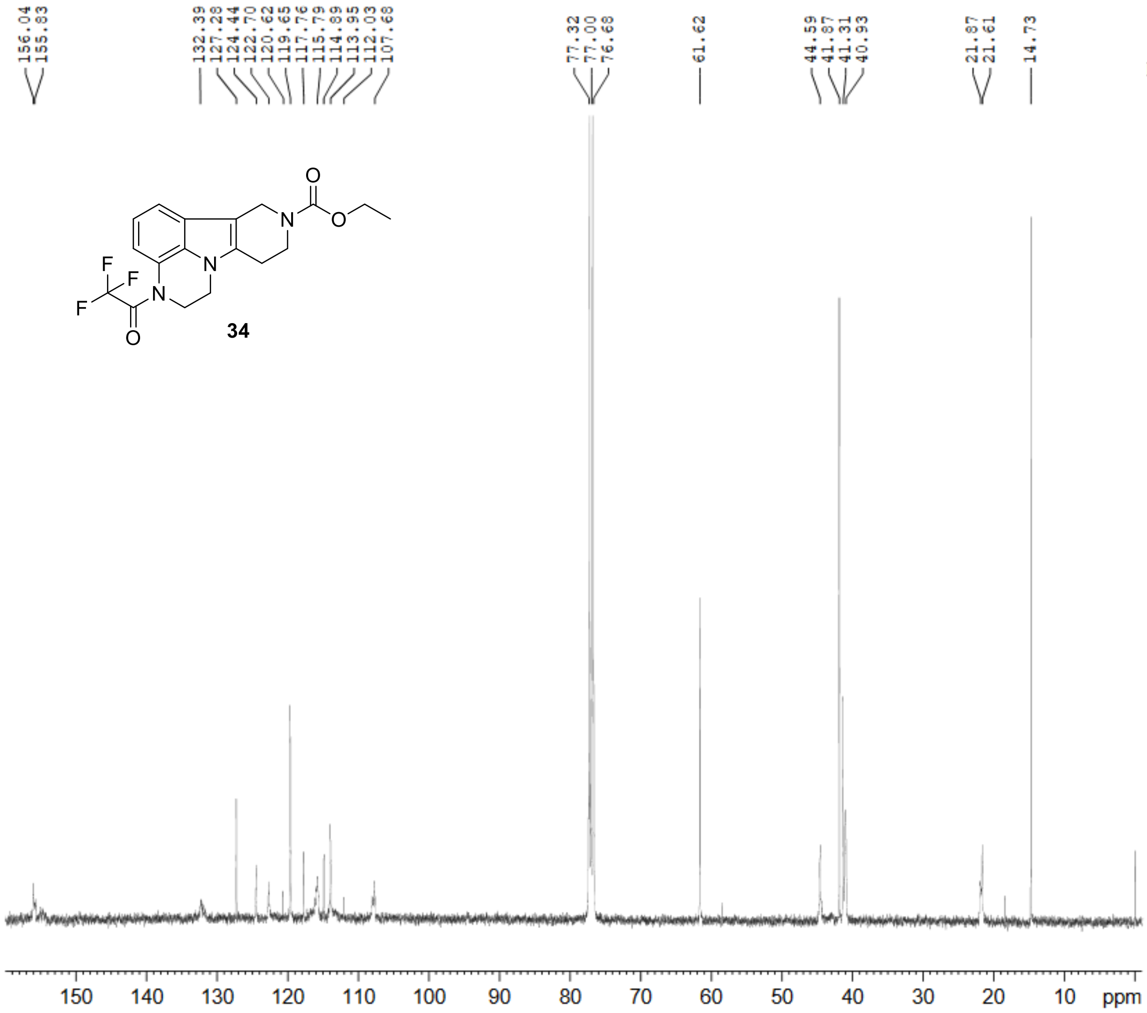


```
Standard 13C
125192
JET0042_1
Milen Matyas
2018.03.14. (DA)

Current Data Parameters
NAME              125192
EXPNO                  2
PROCNO                 1

F2 - Acquisition Parameters
Date_           20180315
Time               10.42 h
INSTRUM            spect
PROBHD   Z105190_0010 (
PULPROG          zgpg30
TD                65536
SOLVENT           CDCl3
NS                16384
DS                    4
SWH           24038.461 Hz
FIDRES         0.733596 Hz
AQ            1.3631488 sec
RG                 2050
DW               20.800 usec
DE                 6.50 usec
TE                293.9 K
D1           1.00000000 sec
D11          0.03000000 sec
TD0                   1
SFO1        100.6228298 MHz
NUC1                13C
P1                 8.80 usec
PLW1        46.20000076 W
SFO2        400.1316005 MHz
NUC2                 1H
CPDPRG[2        waltz16
PCPD2             90.00 usec
PLW2        11.39999962 W
PLW12        0.22700000 W
PLW13        0.11418000 W

F2 - Processing parameters
SI                32768
SF          100.6127722 MHz
WDW                  EM
SSB      0
LB                 1.00 Hz
GB       0
PC                 1.40
```

**122535**	Milen Matyas	MM		BRUKER Alpha
**LVE0031_1**	KBr	27/06/2017		Resolution: 2 cm-1
				Number of Scans: 16

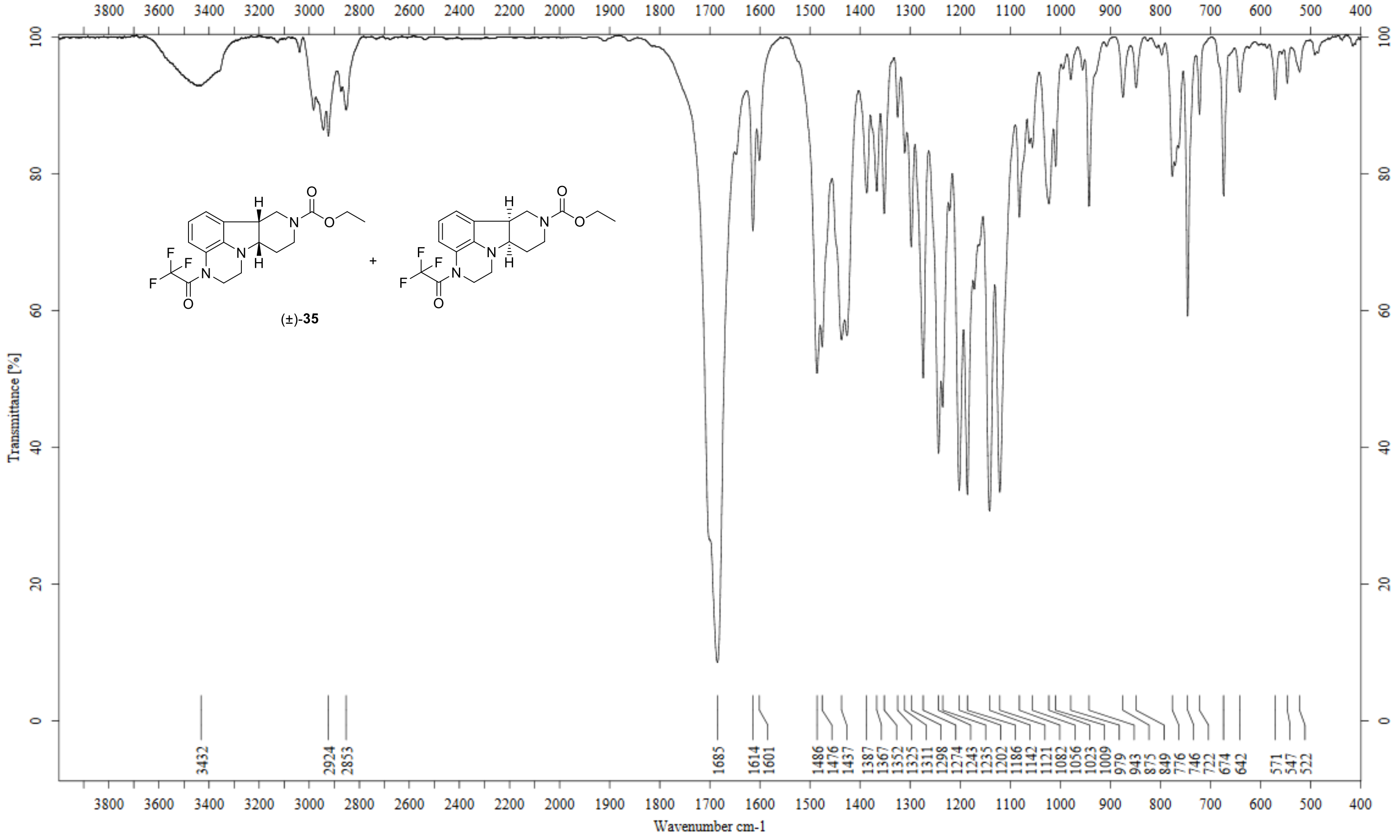

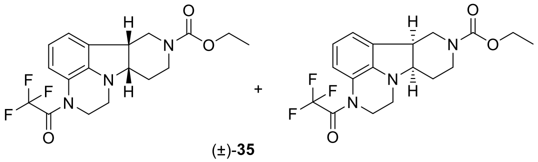

(±)-35

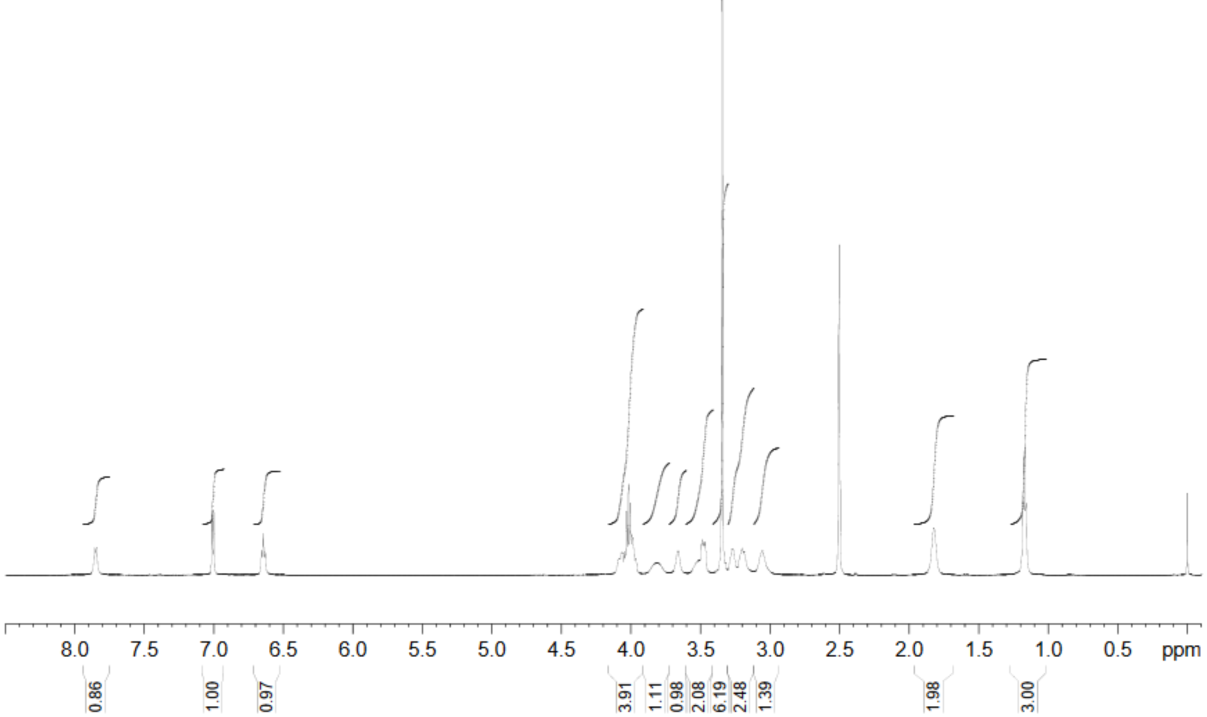

Standard 1H
122535
LVE0031_1
Milen Matyas
2017.06.28. (HJ)

Current Data Parameters

NAME	122535
EXPNO	11
PROCNO	1

F2 - Acquisition Parameters

Date_	20170628
Time	16.29
INSTRUM	spect
PROBHD	5 mm CPPBBO BB
PULPROG	zg30
TD	65536
SOLVENT	DMSO
NS	16
DS	4
SWH	12626.263 Hz
FIDRES	0.192661 Hz
AQ	2.5952256 sec
RG	196.07
DW	39.600 usec
DE	25.00 usec
TE	295.0 K
D1	1.00000000 sec
TD0	1

======== CHANNEL f1 ========

SFO1	600.1642011 MHz
NUC1	1H
P1	11.50 usec
PLW1	28.00000000 W

F2 - Processing parameters

SI	65536
SF	600.1600028 MHz
WDW	EM
SSB	0
LB	0.10 Hz
GB	0
PC	1.00

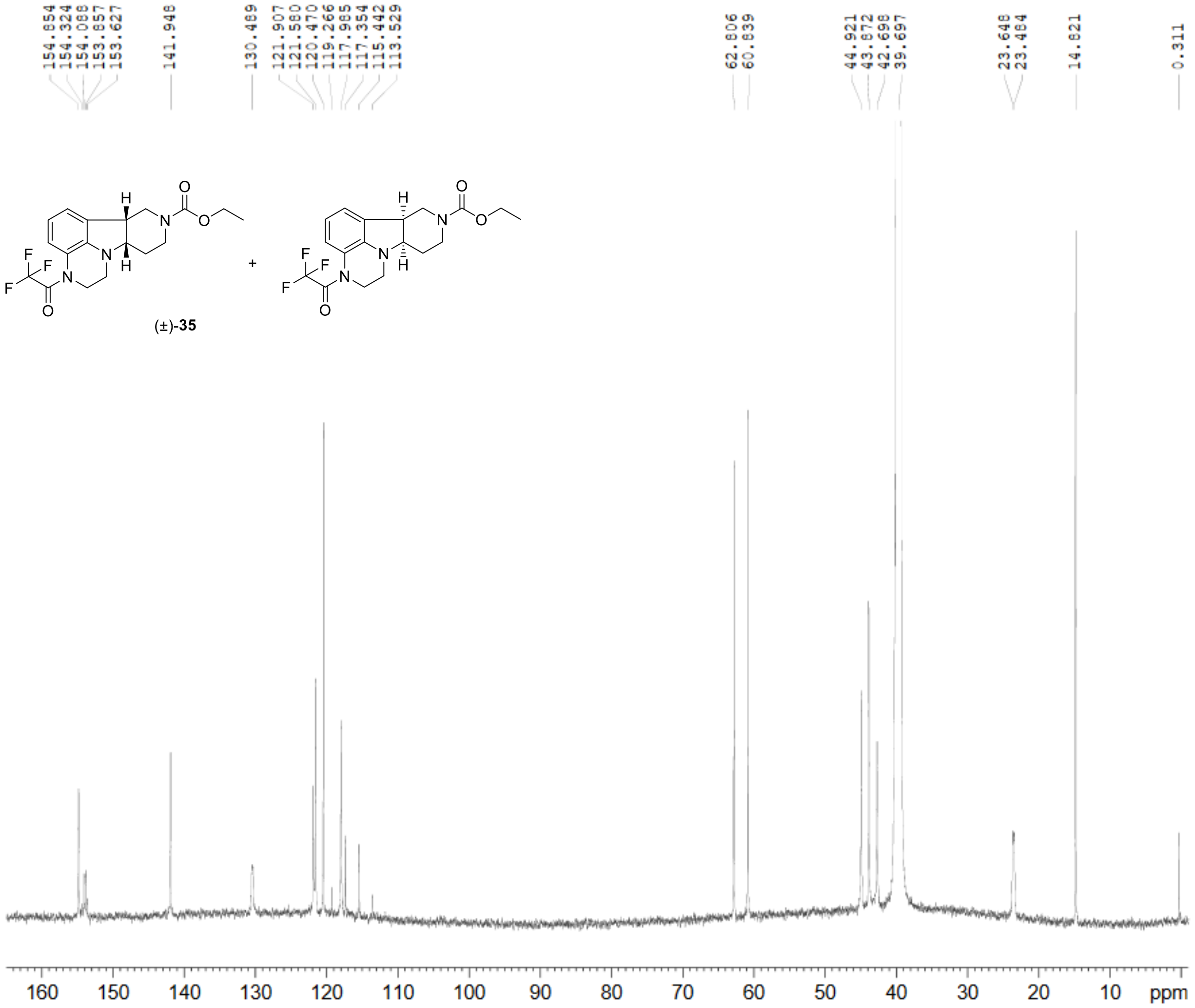

Standard 13C
122535
LVE0031_1
Milen Matyas
2017.06.28. (HJ)

Current Data Parameters

NAME	122535
EXPNO	12
PROCNO	1

F2 - Acquisition Parameters

Date_	20170628
Time	12.40
INSTRUM	spect
PROBHD	5 mm CPPBBO BB
PULPROG	zgpg30
TD	65536
SOLVENT	DMSO
NS	4096
DS	4
SWH	36057.691 Hz
FIDRES	0.550197 Hz
AQ	0.9087659 sec
RG	196.07
DW	13.867 usec
DE	18.00 usec
TE	295.0 K
D1	1.50000000 sec
D11	0.03000000 sec
TD0	1

======== CHANNEL f1 ========

SFO1	150.9254424 MHz
NUC1	13C
P1	10.00 usec
PLW1	65.00000000 W

======== CHANNEL f2 ========

SFO2	600.1624006 MHz
NUC2	1H
CPDPRG[2	waltz16
PCPD2	70.00 usec
PLW2	28.00000000 W
PLW12	0.75571001 W
PLW13	0.37029999 W

F2 - Processing parameters

SI	65536
SF	150.9103921 MHz
WDW	EM
SSB	0
LB	3.00 Hz
GB	0
PC	1.20

125261	Milen Matyas	KP	BRUKER Alpha
			Resolution: 2 cm-1
JET0044_1	KBr	2018.03.21.	Number of Scans: 16

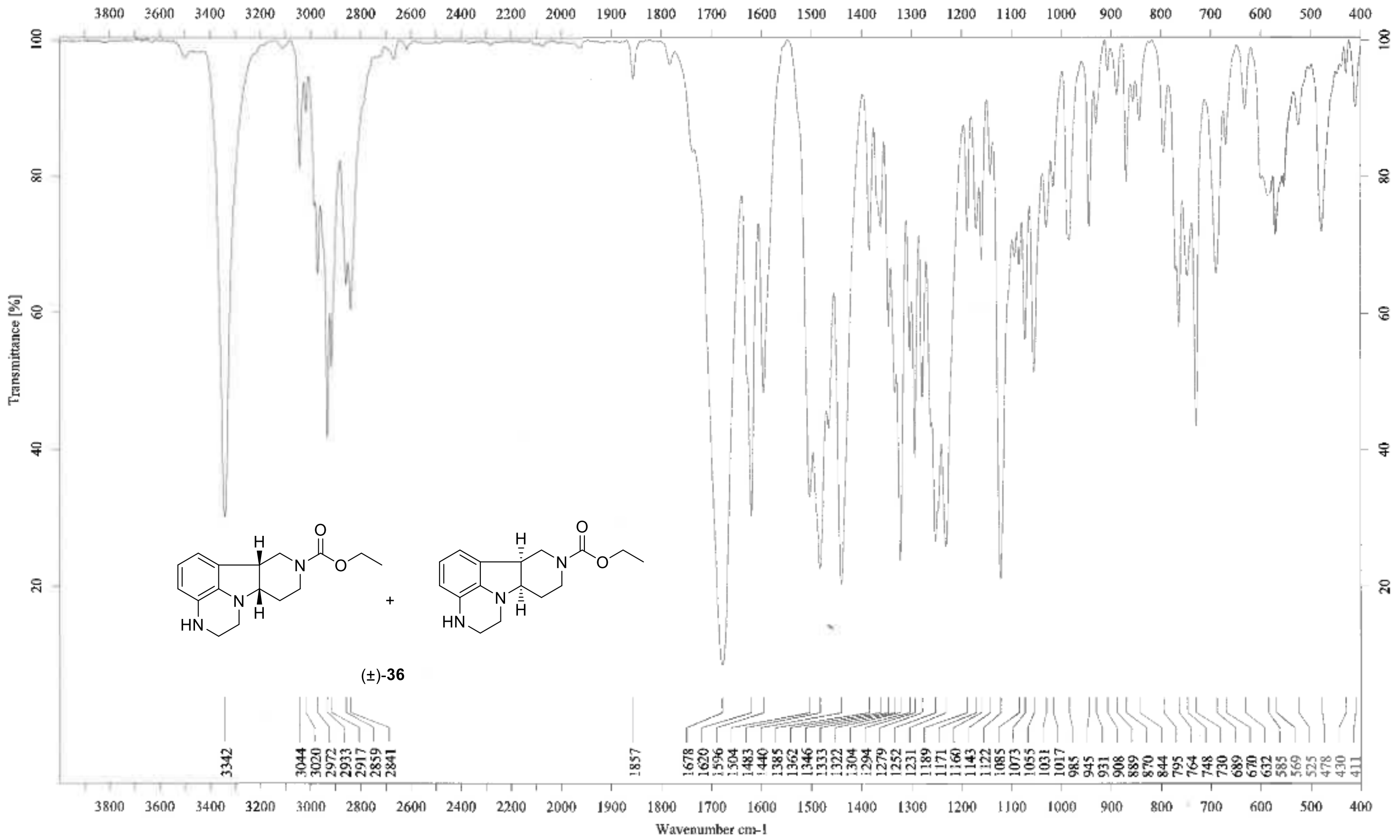

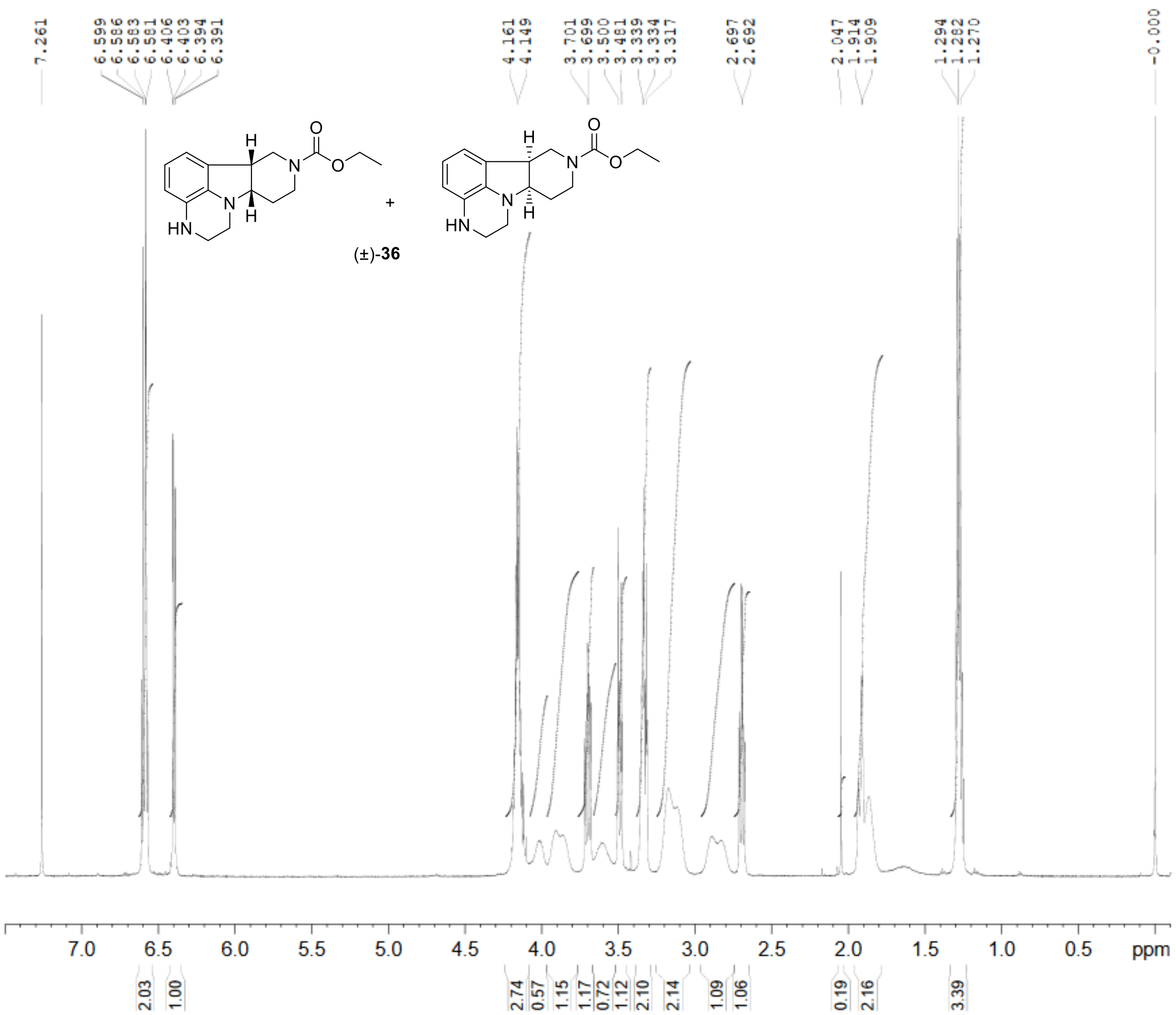


```
Standard 1H
125261
JET0044_1
Milen Matyas
2018.03.21. (DA)

Current Data Parameters
NAME              125261
EXPNO                 11
PROCNO                 1

F2 - Acquisition Parameters
Date_           20180321
Time               23.06 h
INSTRUM            spect
PROBHD   Z145856_0002 (
PULPROG             zg30
TD                 65536
SOLVENT            CDCl3
NS                    16
DS                     4
SWH            12626.263 Hz
FIDRES          0.385323 Hz
AQ             2.5952256 sec
RG                155.77
DW                39.600 usec
DE                 25.00 usec
TE                 295.0 K
D1            1.00000000 sec
TD0                    1
SFO1         600.1642011 MHz
NUC1                  1H
P1                 11.50 usec
PLW1         28.00000000 W

F2 - Processing parameters
SI                 65536
SF           600.1600144 MHz
WDW                   no
SSB       0
LB        0 Hz
GB        0
PC                  1.00
```

(±)-**36**

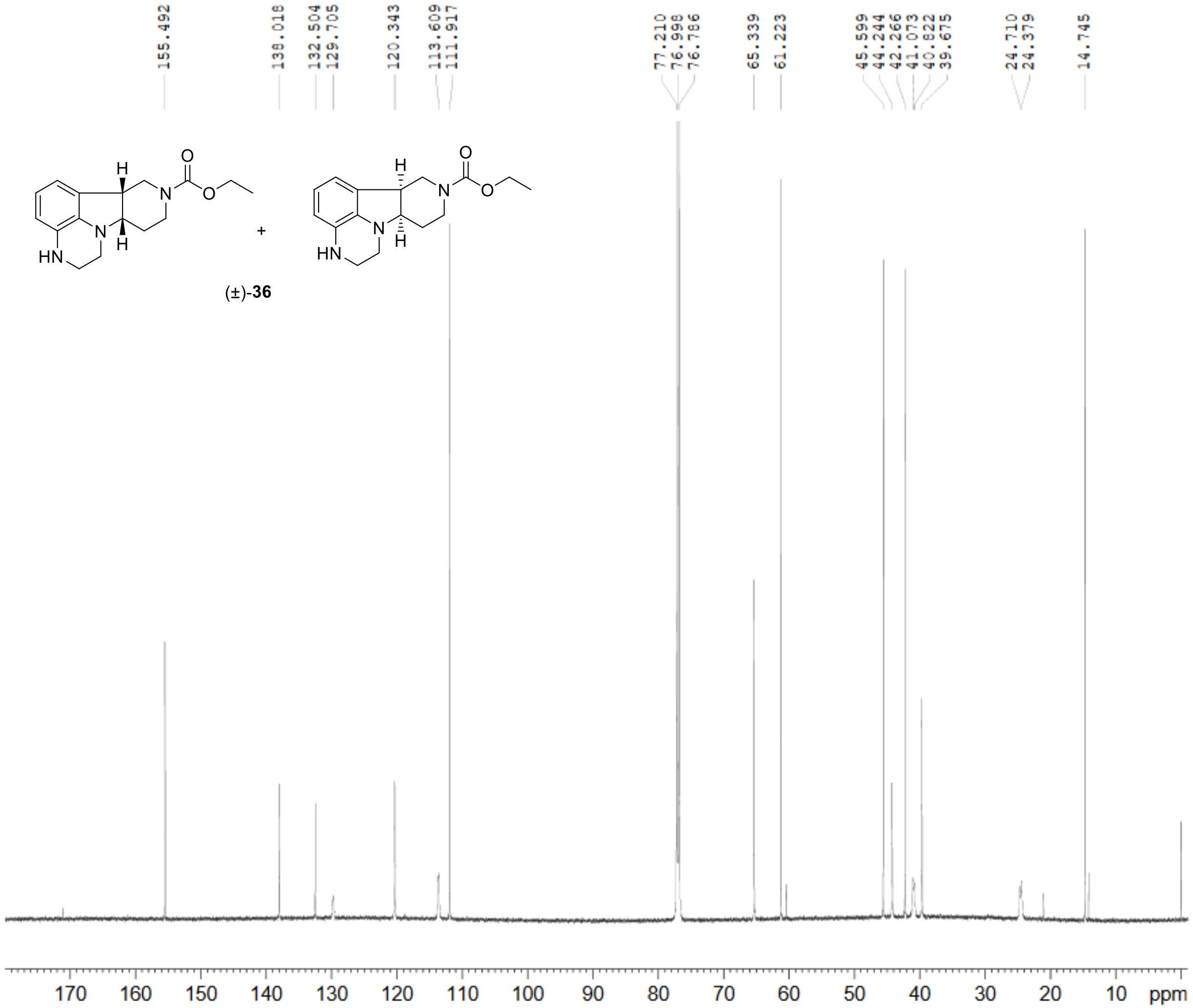

Standard 13C
125261
JET0044_1
Milen Matyas
2018.03.21. (DA)

Current Data Parameters

NAME	125261
EXPNO	12
PROCNO	1

F2 - Acquisition Parameters

Date_	20180322
Time	1.56 h
INSTRUM	spect
PROBHD	Z145856_0002 (
PULPROG	zgpg30
TD	65536
SOLVENT	CDCl3
NS	4096
DS	4
SWH	36057.691 Hz
FIDRES	1.100393 Hz
AQ	0.9087659 sec
RG	196.07
DW	13.867 usec
DE	18.00 usec
TE	295.0 K
D1	1.50000000 sec
D11	0.03000000 sec
TD0	1
SFO1	150.9254424 MHz
NUC1	13C
P1	10.00 usec
PLW1	60.36299896 W
SFO2	600.1624006 MHz
NUC2	1H
CPDPRG[2	waltz16
PCPD2	80.00 usec
PLW2	26.42600060 W
PLW12	0.59460002 W
PLW13	0.29861000 W

F2 - Processing parameters

SI	65536
SF	150.9103602 MHz
WDW	EM
SSB	0
LB	1.00 Hz
GB	0
PC	1.40

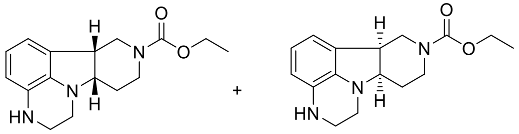

(±)-**36**

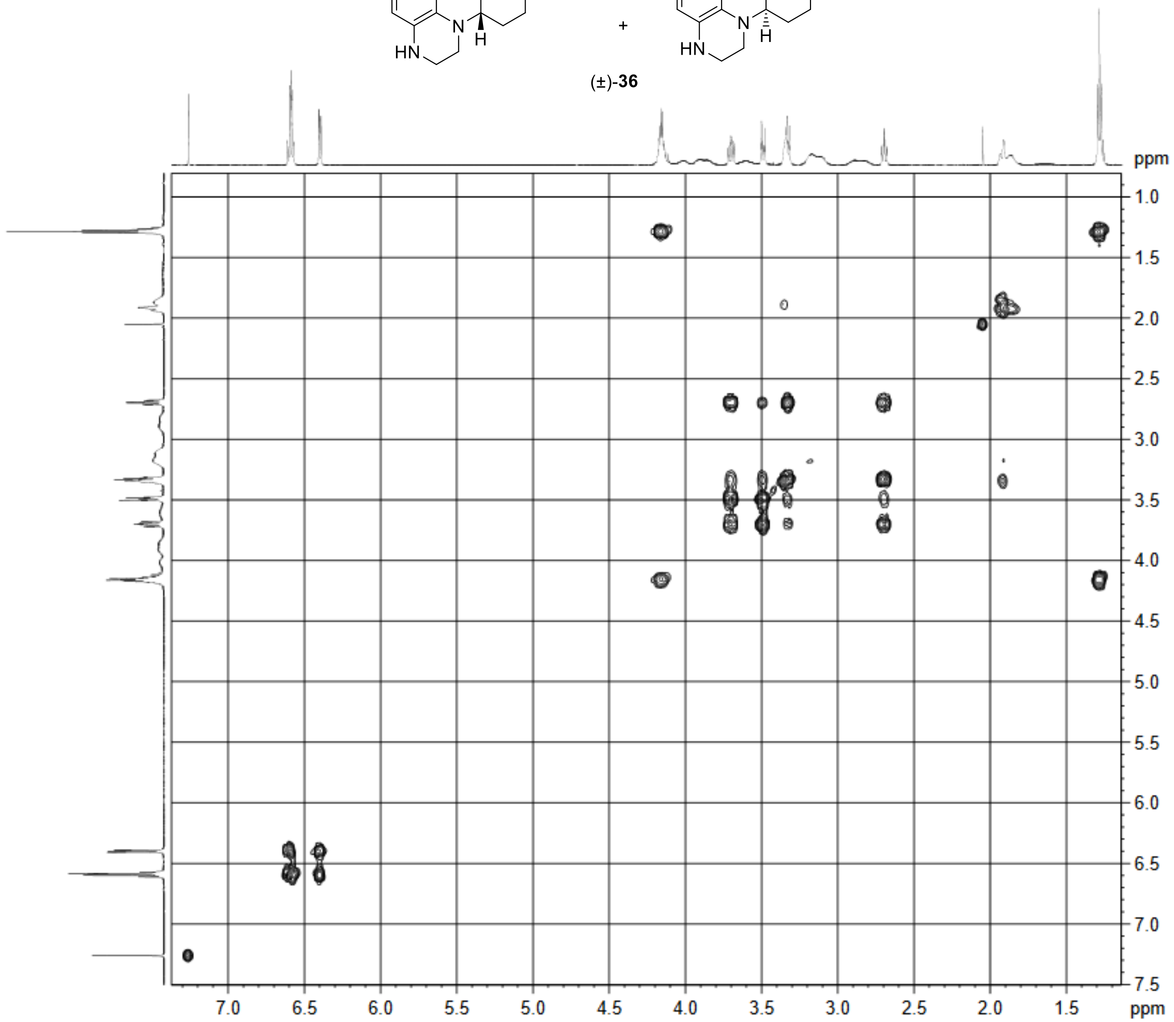

COSY
125261
JET0044_1
Milen Matyas
2018.03.21. (DA)

Current Data Parameters
NAME 125261
EXPNO 13
PROCNO 1

F2 - Acquisition Parameters
Date_ 20180322
Time 1.57 h
INSTRUM spect
PROBHD Z145856_0002 (
PULPROG cosygpppqf
TD 2048
SOLVENT CDCl3
NS 2
DS 8
SWH 8012.820 Hz
FIDRES 7.825020 Hz
AQ 0.1277952 sec
RG 196.07
DW 62.400 usec
DE 25.00 usec
TE 295.0 K
D0 0.00000300 sec
D1 2.00000000 sec
D11 0.03000000 sec
D12 0.00002000 sec
D13 0.00000400 sec
D16 0.00020000 sec
IN0 0.00012480 sec
TDav 1
SFO1 600.1636083 MHz
NUC1 1H
P0 11.50 usec
P1 11.50 usec
P17 2500.00 usec
PLW1 28.00000000 W
PLW10 5.92479992 W
GPNAM[1] SMSQ10.100
GPZ1 10.00 %
P16 1000.00 usec

F1 - Acquisition parameters
TD 256
SFO1 600.1636 MHz
FIDRES 62.600159 Hz
SW 13.351 ppm
FnMODE QF

F2 - Processing parameters
SI 1024
SF 600.1600145 MHz
WDW QSINE
SSB 0
LB 0 Hz
GB 0
PC 1.40

F1 - Processing parameters
SI 1024
MC2 QF
SF 600.1600120 MHz
WDW QSINE
SSB 0
LB 0 Hz
GB 0

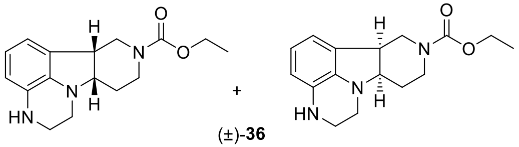

(±)-**36**

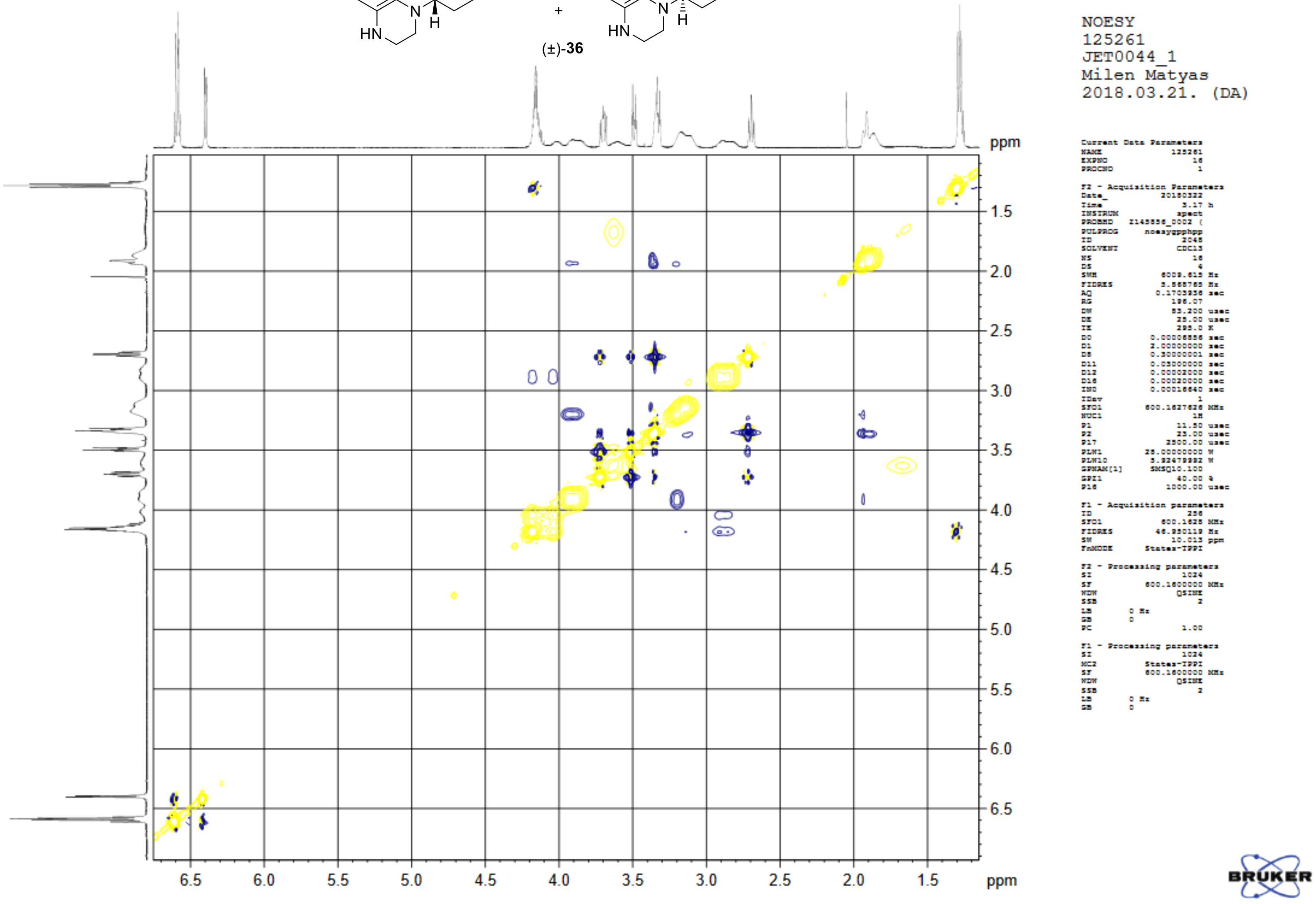
NOESY
125261
JET0044_1
Milen Matyas
2018.03.21. (DA)
ppm
6.5
6.0
5.5
5.0
4.5
4.0
3.5
3.0
2.5
2.0
1.5
ppm
1.5
2.0
2.5
3.0
3.5
4.0
4.5
5.0
5.5
6.0
6.5
BRUKER

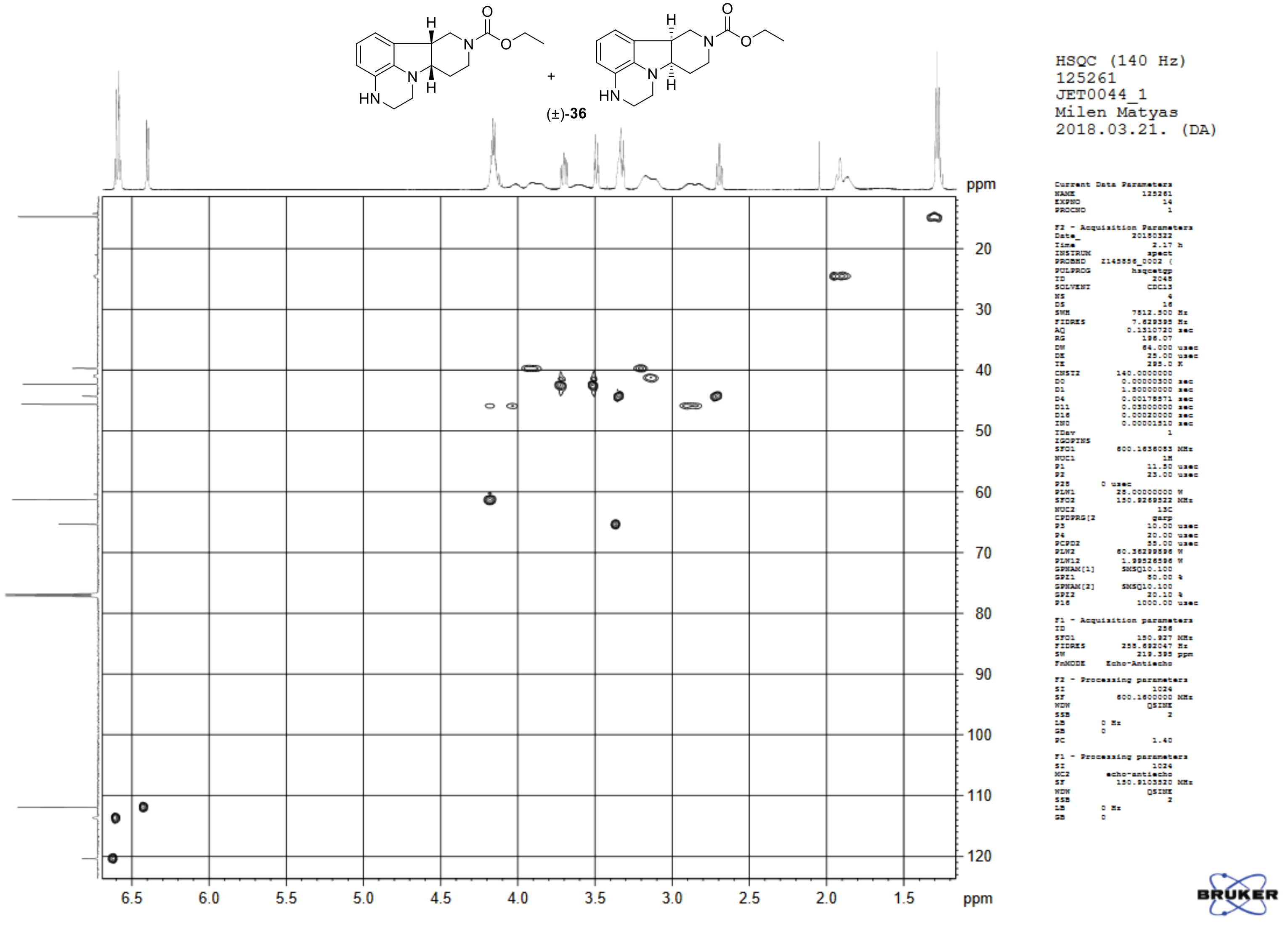
(±)-36
HSQC (140 Hz)
125261
JET0044_1
Milen Matyas
2018.03.21. (DA)
ppm
BRUKER

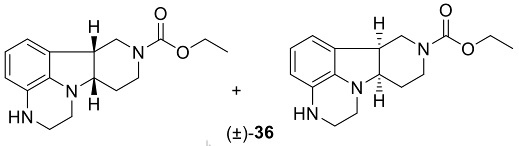

(±)-36

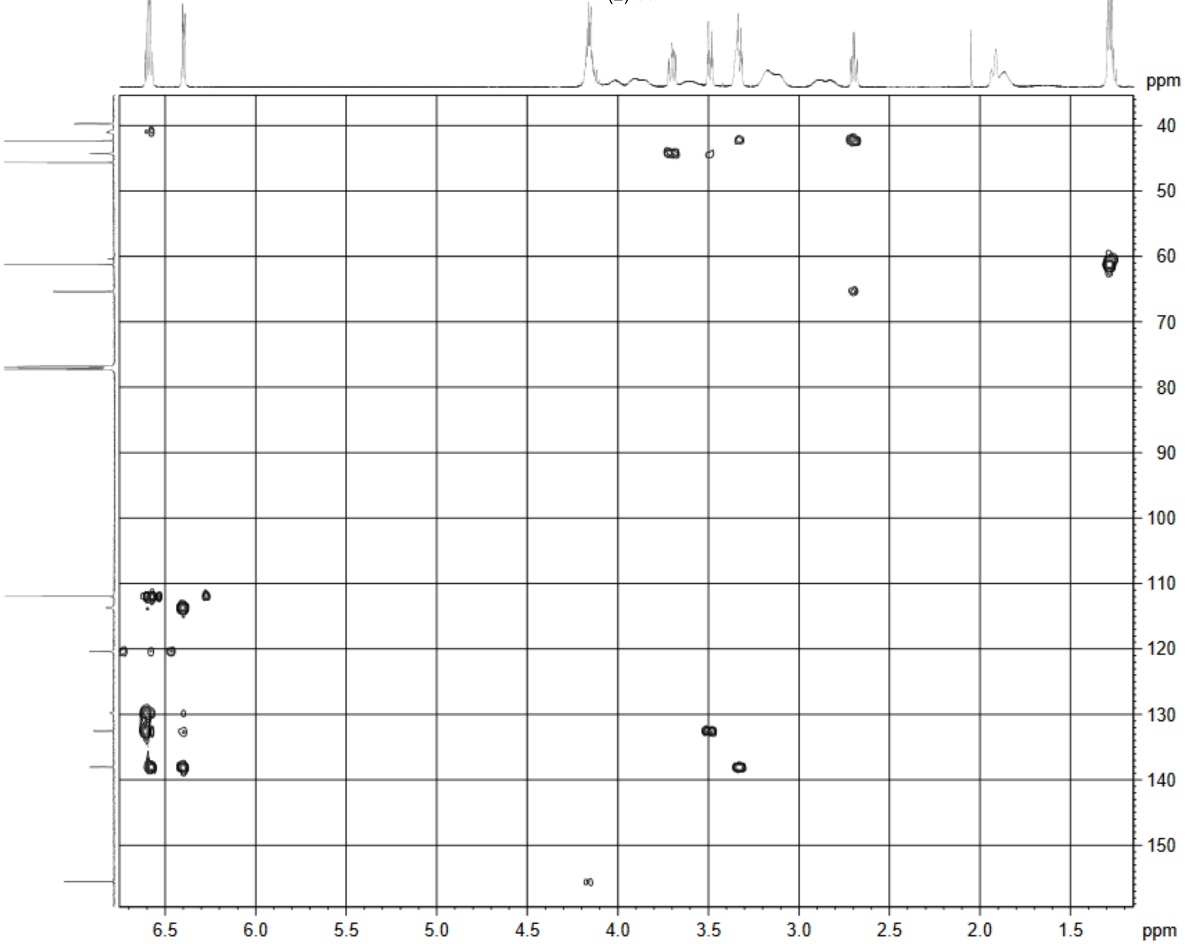

HMBC (8 Hz, 140 Hz)
125261
JET0044_1
Milen Matyas
2018.03.21. (DA)

Current Data Parameters
NAME 125261
EXPNO 15
PROCNO 1

F2 - Acquisition Parameters
Date_ 20180322
Time 2.47 h
INSTRUM spect
PROBHD Z145556_0002 (
PULPROG hmbcgplpndqf
TD 2048
SOLVENT CDCl3
NS 4
DS 16
SWH 7812.500 Hz
FIDRES 7.629395 Hz
AQ 0.1310720 sec
RG 196.07
DW 64.000 usec
DE 25.00 usec
TE 295.0 K
CNST2 140.0000000
CNST13 8.0000000
D0 0.00000300 sec
D1 1.50000000 sec
D2 0.00357143 sec
D6 0.06250000 sec
D16 0.00020000 sec
IN0 0.00001510 sec
TDav 1
SFO1 600.1639010 MHz
NUC1 1H
P1 11.50 usec
P2 23.00 usec
PLW1 25.00000000 W
SFO2 150.9269522 MHz
NUC2 13C
P3 10.00 usec
PLW2 60.36299896 W
GPNAM[1] SMSQ10.100
GPZ1 50.00 %
GPNAM[2] SMSQ10.100
GPZ2 30.00 %
GPNAM[3] SMSQ10.100
GPZ3 40.10 %
P16 1000.00 usec

F1 - Acquisition parameters
TD 256
SFO1 150.927 MHz
FIDRES 255.692047 Hz
SW 219.395 ppm
FnMODE QF

F2 - Processing parameters
SI 2048
SF 600.1600136 MHz
WDW SINE
SSB 0
LB 0 Hz
GB 0
PC 1.40

F1 - Processing parameters
SI 512
MC2 QF
SF 150.9103569 MHz
WDW SINE
SSB 0
LB 0 Hz
GB 0

BRUKER

**125274**	Milen Matyas	KP	BRUKER Alpha
			Resolution: 2 cm-1
**JET0043_1**	Film	2018.03.23.	Number of Scans: 16

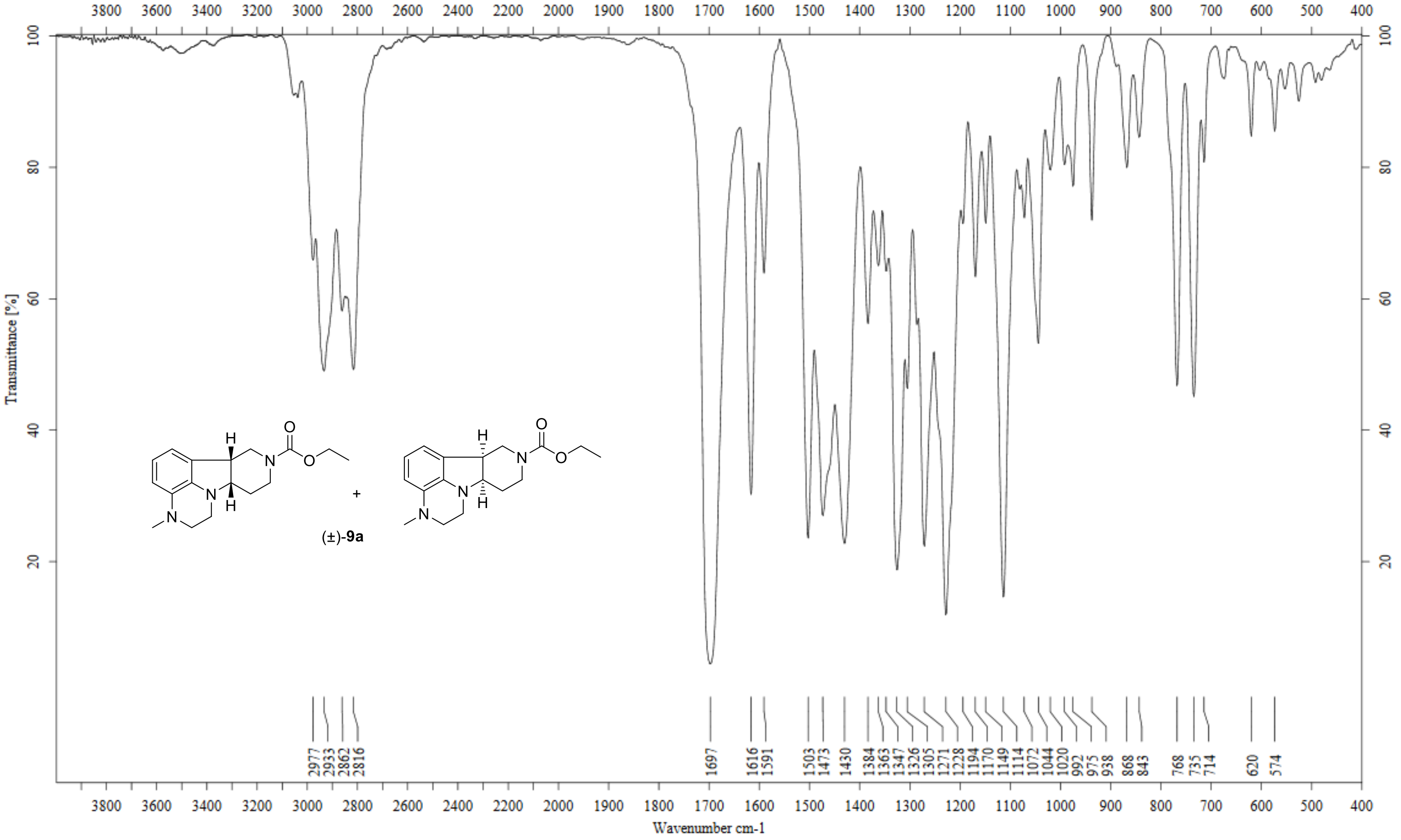

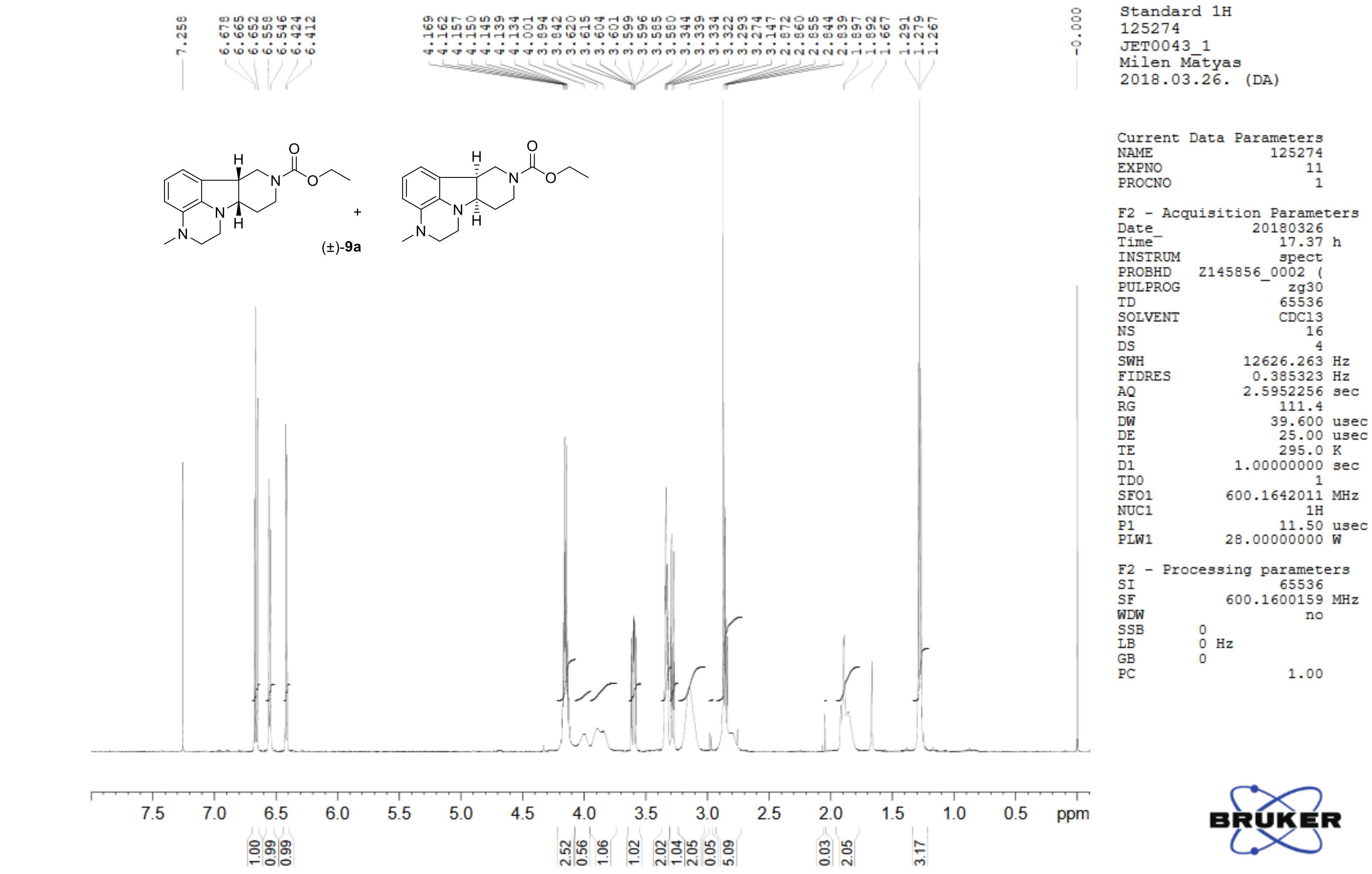

(±)-9a
Standard 1H
125274
JET0043_1
Milen Matyas
2018.03.26. (DA)
Current Data Parameters
NAME 125274
EXPNO 11
PROCNO 1
F2 - Acquisition Parameters
Date_ 20180326
Time_ 17.37 h
INSTRUM spect
PROBHD Z145856_0002 (
PULPROG zg30
TD 65536
SOLVENT CDCl3
NS 16
DS 4
SWH 12626.263 Hz
FIDRES 0.385323 Hz
AQ 2.5952256 sec
RG 111.4
DW 39.600 usec
DE 25.00 usec
TE 295.0 K
D1 1.00000000 sec
TD0 1
SFO1 600.1642011 MHz
NUC1 1H
P1 11.50 usec
PLW1 28.00000000 W
F2 - Processing parameters
SI 65536
SF 600.1600159 MHz
WDW no
SSB 0
LB 0 Hz
GB 0
PC 1.00
ppm

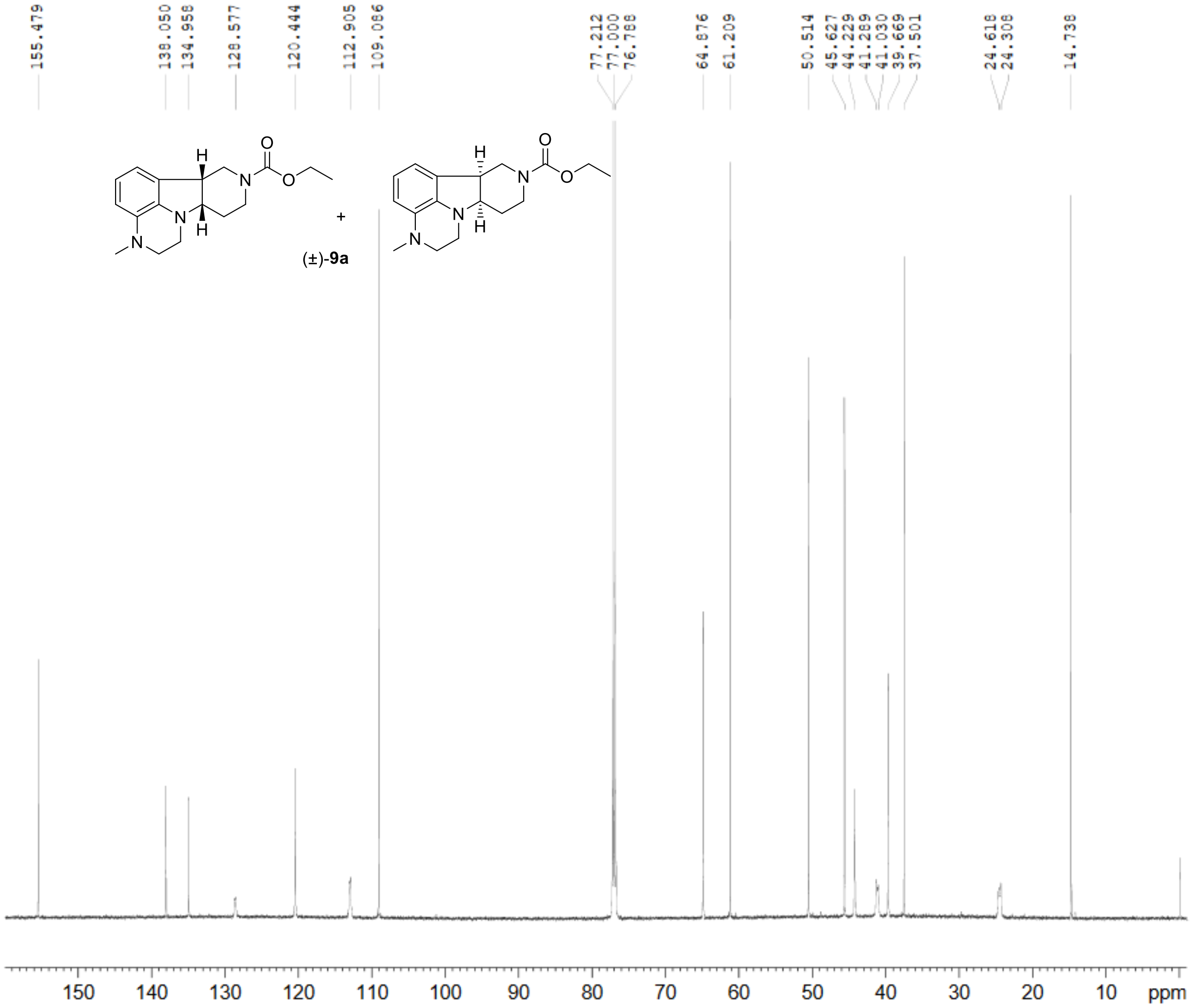

Standard 13C
125274
JET0043_1
Milen Matyas
2018.03.26. (DA)

Current Data Parameters
NAME 125274
EXPNO 12
PROCNO 1

F2 - Acquisition Parameters
Date_ 20180326
Time 19.03 h
INSTRUM spect
PROBHD Z145856_0002 (
PULPROG zgpg30
TD 65536
SOLVENT CDCl3
NS 2048
DS 4
SWH 36057.691 Hz
FIDRES 1.100393 Hz
AQ 0.9087659 sec
RG 196.07
DW 13.867 usec
DE 18.00 usec
TE 295.0 K
D1 1.50000000 sec
D11 0.03000000 sec
TD0 1
SFO1 150.9254424 MHz
NUC1 13C
P1 10.00 usec
PLW1 60.36299896 W
SFO2 600.1624006 MHz
NUC2 1H
CPDPRG[2 waltz16
PCPD2 80.00 usec
PLW2 26.42600060 W
PLW12 0.59460002 W
PLW13 0.29861000 W

F2 - Processing parameters
SI 65536
SF 150.9103614 MHz
WDW EM
SSB 0
LB 1.00 Hz
GB 0
PC 1.40
